# SESMic: A Reagent for the Synthesis of 5‑Membered Heteroaryl Masked Sulfinates

**DOI:** 10.1021/acs.orglett.6c02962

**Published:** 2026-08-05

**Authors:** Yumeng Chi, Geoffrey H. Chan, Matthew J. Moschitto

**Affiliations:** Department of Medicinal Chemistry, Ernest Mario School of Pharmacy, 242612 Rutgers, the State University of New Jersey, 163 Frelinghuysen Road, Piscataway, New Jersey 08901-8554, United States

## Abstract

Sulfur­(VI) functionalities are found in numerous value-added products; however, methods for the synthesis of heterocyclic sulfonyl fluorides are limited. 4-Substituted sulfonyl fluoride azoles could be useful synthons in drug discovery and biochemical applications due to their enhanced stability derived from their electron-rich nature. In this study, we develop an isocyanide reagent, SESMic, that contains a sulfinate protecting group allowing for synthesis of a variety of heterocycles followed by sulfinate unmasking and further functionalization. SESMic specifically allows access to 4-substituted sulfonyl fluorides, which were shown to be more stable than their aryl congeners.

## Graphical abstract

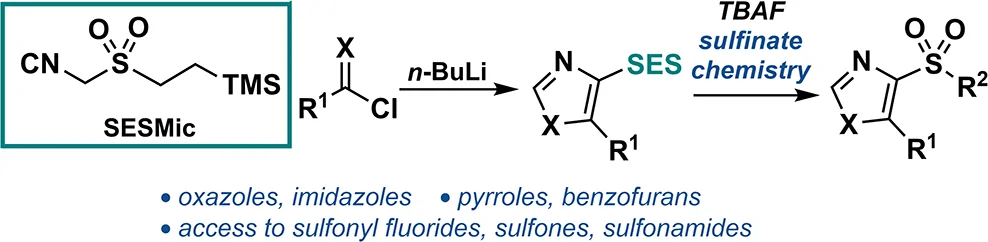

Sulfur­(VI)-containing compounds are important motifs found in many biologically active compounds.^1–5^ Sulfonyl fluorides (SFs), in particular, have seen increased use due to their click-like reactivity through sulfur-fluoride exchange (SuFEx) chemistry.^6,7^ From a synthetic standpoint, SFs are often installed at the end of a synthetic route, thus limiting the structural diversity. Often, sulfur is incorporated into the core scaffold from either the commercial sulfonyl chloride or thiol. Our group is particularly interested in electron-rich SFs as the increase in electron density affords a more stable species, thus improving their utility in drug discovery applications (Figure 1a).^8–10^ The strategic placement of an SF at electron-rich positions of five-membered heterocycles could therefore lead to privileged covalent warheads that afford improved stability and favorable physicochemical properties (Figure 1a).^11,12^ In this context, 4-sulfonyl azoles, including oxazoles and imidazoles, represent promising covalent scaffolds. Consistent with this hypothesis, enhanced stability has previously been observed for pyrrole-3-sulfonyl fluorides.^8^

**Figure 1:**
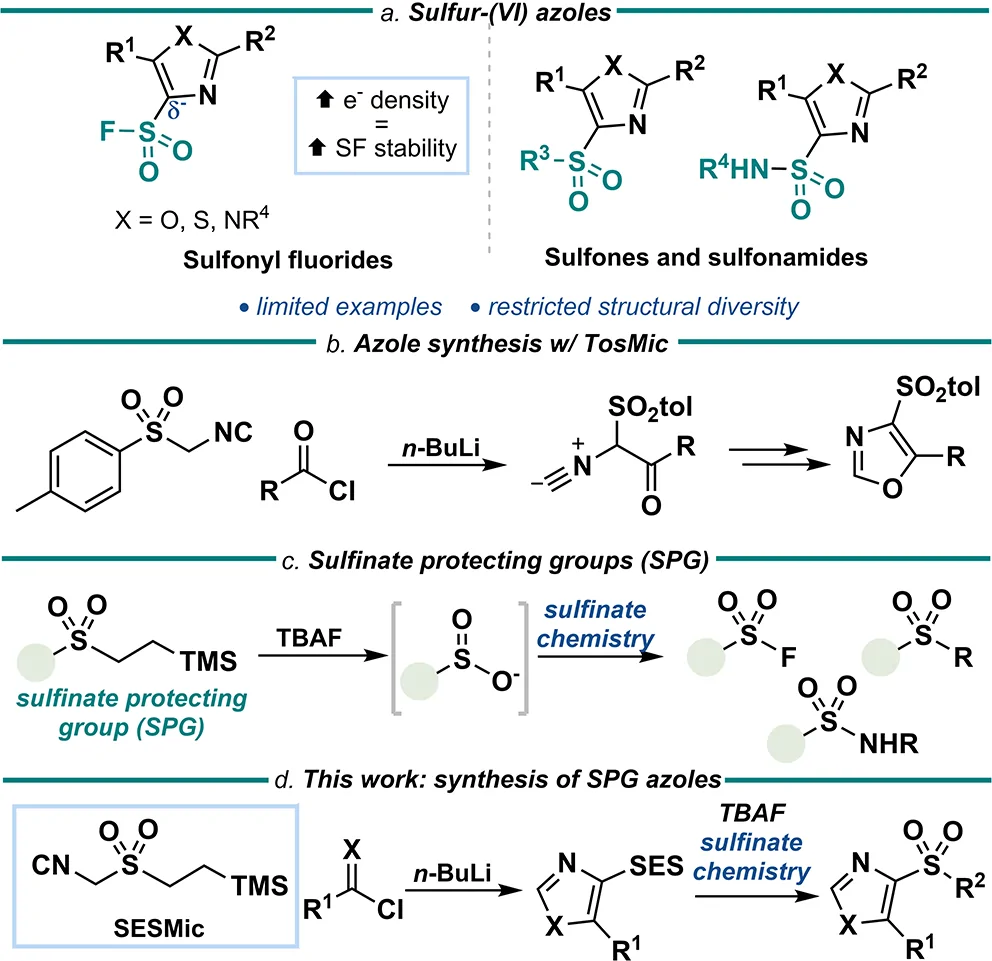
Masked sulfinate sulfur­(VI) azoles.

At the same time, the ability to diversify a single common intermediate into a variety of sulfur-(VI) functionalities such as sulfonamides and sulfones is equally important in compound diversification studies. One of the most common methods to synthesize azoles is with the reagent *p*-tolylsulfonylmethyl isocyanide (TosMic).^13^ As first described by Van Leusen, TosMic can react with activated carboxylates (such as acid chlorides) or aldehydes to form substituted oxazoles (Figure 1b).^14^ In the Van Leusen reaction, the sulfonyl group typically acts as a leaving group to complete the final aromatization; however, when other leaving groups are present (e.g., a chloride), the sulfonyl group can be retained.^14^ TosMic is also known in the synthesis of additional 5-membered heterocyclic species such as benzazole and pyrrole.^15,16^ While derivatives of TosMic exist, most contain only aryl functionality.^17^ Alternative approaches to 4-substituted azoles include the oxidation of the corresponding thioether,^18–20^ itself obtained from S_N_Ar, transition-metal-catalyzed reaction of diazosulfones with nitriles,^21,22^ and a variety of cycloaddition- and cross-coupling-based methodologies.^22–26^ Despite these advances, access to specific sulfonyl-substituted heterocycles generally requires the preparation of a tailored precursor, such as a functionalized isocyanide or diazosulfone, for each desired target. As a result, a modular strategy that enables the rapid synthesis and diversification of sulfonyl-containing heterocycles from a common intermediate is highly valuable. Furthermore, the ability to decouple the installation of sulfur-(VI) functionality from downstream diversification will further expand chemical space by allowing additional chemical transformations.

This modularity can be achieved with the synthesis of sulfinate protecting groups (SPGs), which allow for the installation of sulfur at any point in a synthetic endeavor (Figure 1c).^27–30^ These masked sulfinates can be deprotected and then converted into a variety of sulfur-(VI) functionalities. Our group recently evaluated the stability and utility of a variety of SPGs and found that the trimethylsilyl ethyl sulfone (SES) group possesses a high level of stability across numerous synthetic conditions including basic and nucleophilic conditions.^27^ A single example employing a diazo-SPG currently exists from the laboratory of Matsunaga for the synthesis of SPG-containing azoles employing a silyloxymethyl sulfone protecting group.^21^ Given the limited methods available to make 4-substituted sulfur­(VI) azoles, we sought to develop a general and modular method for the synthesis of a diverse array of 5-membered heterocycles from a common intermediate and reagent, with a functionality, SES, that has high stability across various reaction conditions. This high level of stability allows for the decoupling of sulfur installation from diversification. With its elevated stability, an isocyanide reagent containing the SES group (SESMic) would allow for the synthesis of SPG-containing heterocycles followed by deprotection and various sulfinate-based chemistries (Figure 1d). This would add to the toolbox available to a medicinal chemist in the synthesis of substituted 5-membered heterocycles.

SESMic is easily synthesized in two steps from the SES sulfinate salt (Figure 2). Purification of SESMic requires an alumina column to remove an unknown impurity (SESMic degrades on silica gel chromatography). Pure SESMic is isolated as an orange semisolid and is stable for at least 2 months at −20 °C. SESMic reacts with acid chlorides upon deprotonation with *n*-BuLi to afford the corresponding oxazole (Table 1). Yields are moderate and are in line with similar reactions using TosMic.^31–33^ Overall, the moderate yields generally result from unreacted acid chloride (and subsequent hydrolysis to the carboxylic acid) and/or degradation of SESMic. Additional equivalents of SESMic and/or *n*-BuLi do not improve yield. *n*-BuLi was the optimal base. This reaction can be operated on a larger scale with a slight reduction in yield. Overall, all yields are reported as two step yields from the carboxylic acid unless otherwise noted.

**Figure 2:**
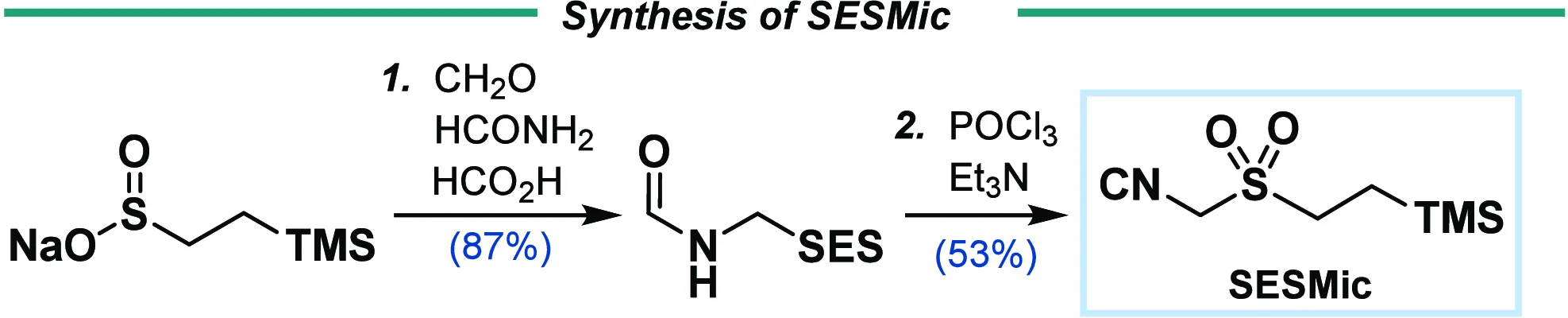
Synthesis of SESMic. Reagents and conditions: (1) CH_2_O (3 equiv), HCONH_2_ (10 equiv), HCO_2_H (3.5 equiv), H_2_O, 4 h, 90 °C, 87% yield; (2) POCl_3_ (2 equiv), Et_3_N (6 equiv), THF, 1 h, 0 °C to rt, 53% yield.

**Table 1:**
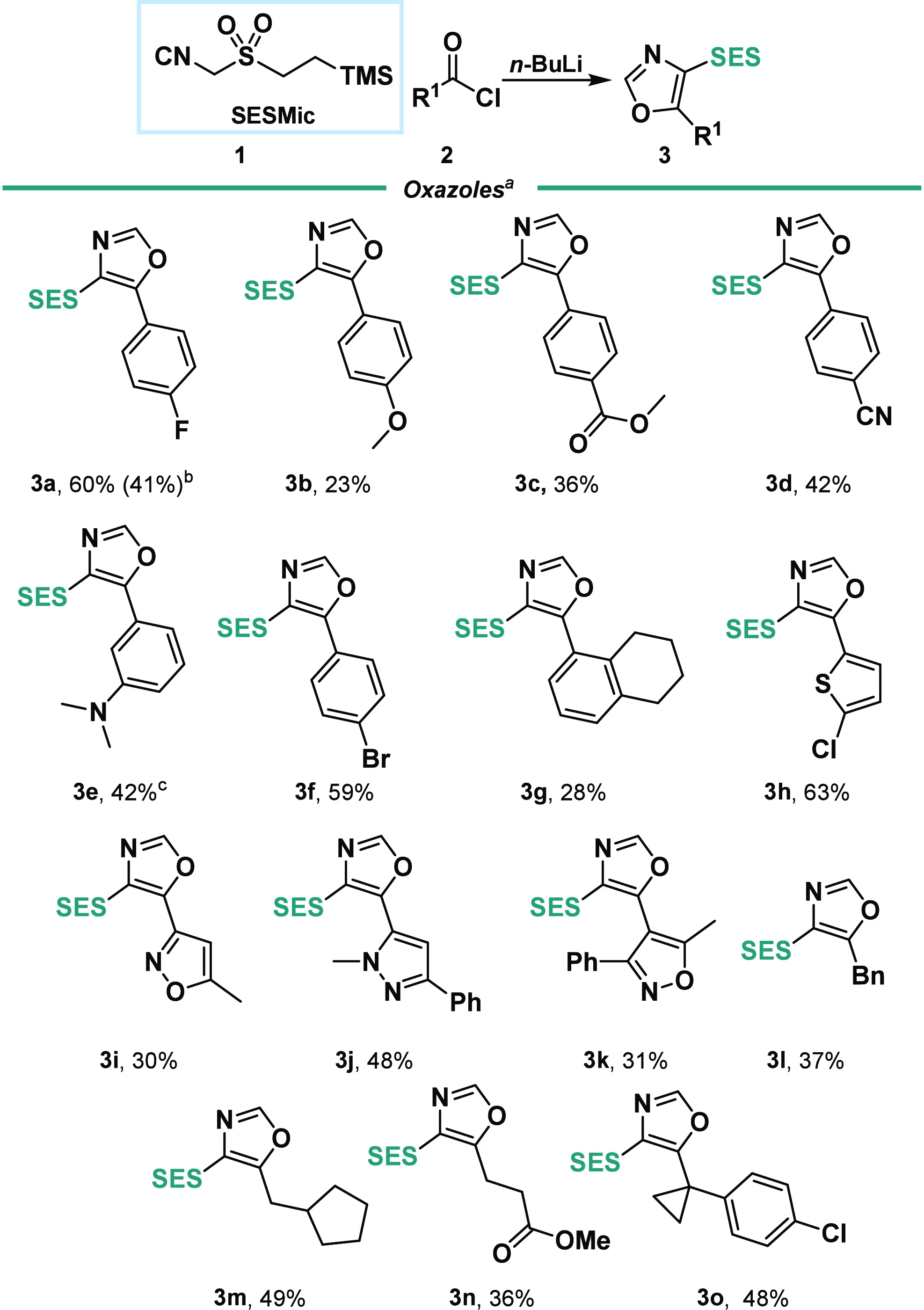
Synthesis of 4-Substituted SES Oxazoles^a^

a Isolated yields shown from the corresponding carboxylic acid on a 0.15–0.25 mmol scale unless otherwise noted. Reagents and conditions: (i) carboxylic acid (1 equiv), SOCl_2_ (1 M), 65 °C 2 h; (ii) SESMIC (1.1 equiv), *n*-BuLi (1.2 equiv), THF, 16 h, −78 °C to rt.

b 2.2 mmol scale from the commercial acid chloride.

c Acyl imidazole, made with CDI, was used instead of acid chloride.

We then investigated the scope. The electron density of the aryl ring did not have an overt effect on yield as both electron-rich (**3b**) and electron-deficient aryl groups (**3c**–**3d**) yielded products. Heterocycles (**3h**–**3k**) were well tolerated, so long as a neutral acid chloride was isolable. Alkyl (**3l**–**3n**) functionalities, including the benzyl (**3l**) group, which may compete for deprotonation, also produced products in moderate yield. Substrates with electrophilic groups (**3c** and **3d**) were competent, and nucleophilic addition to these groups was not observed. Halogen-containing arenes furnished the corresponding oxazoles with no observed loss of the halo group (**3f**, **3h**, **3o**). Sterically hindered aryl (**3g**) or cyclopropyl carboxylic acids (**3o**) also yielded products in modest yields. In most cases, starting carboxylic acids were converted to the corresponding acyl chlorides using thionyl chloride, and these were directly used in the subsequent reaction with SESMic to afford the desired products. In the case of dimethylaniline **3e**, the acyl imidazole, synthesized with CDI, was used. Overall, purity and quality of the acid chloride are important for higher yields. Acryloyl chlorides and pyridine-containing acid chlorides either did not yield product or produced incredibly complex reaction mixtures.

The reaction of SESMic with *N*-formylsaccharin (**4**) provided access to 2-H oxazole (Figure 3a). To further functionalize **5**, sequential cross-coupling with aryl halides was performed. Under ligand-controlled palladium catalysis,^34–37^ cross-coupling first occurs at the 2-position followed by coupling at the 5-position. This provides a sequential platform to build an array of substituted oxazoles, followed by unmasking of the sulfinate (*vide infra*). Previous methods have not provided this level of sequential functionalization, and the stability of the SES group provides a robustness for cross-coupling not seen with other SPGs.

**Figure 3:**
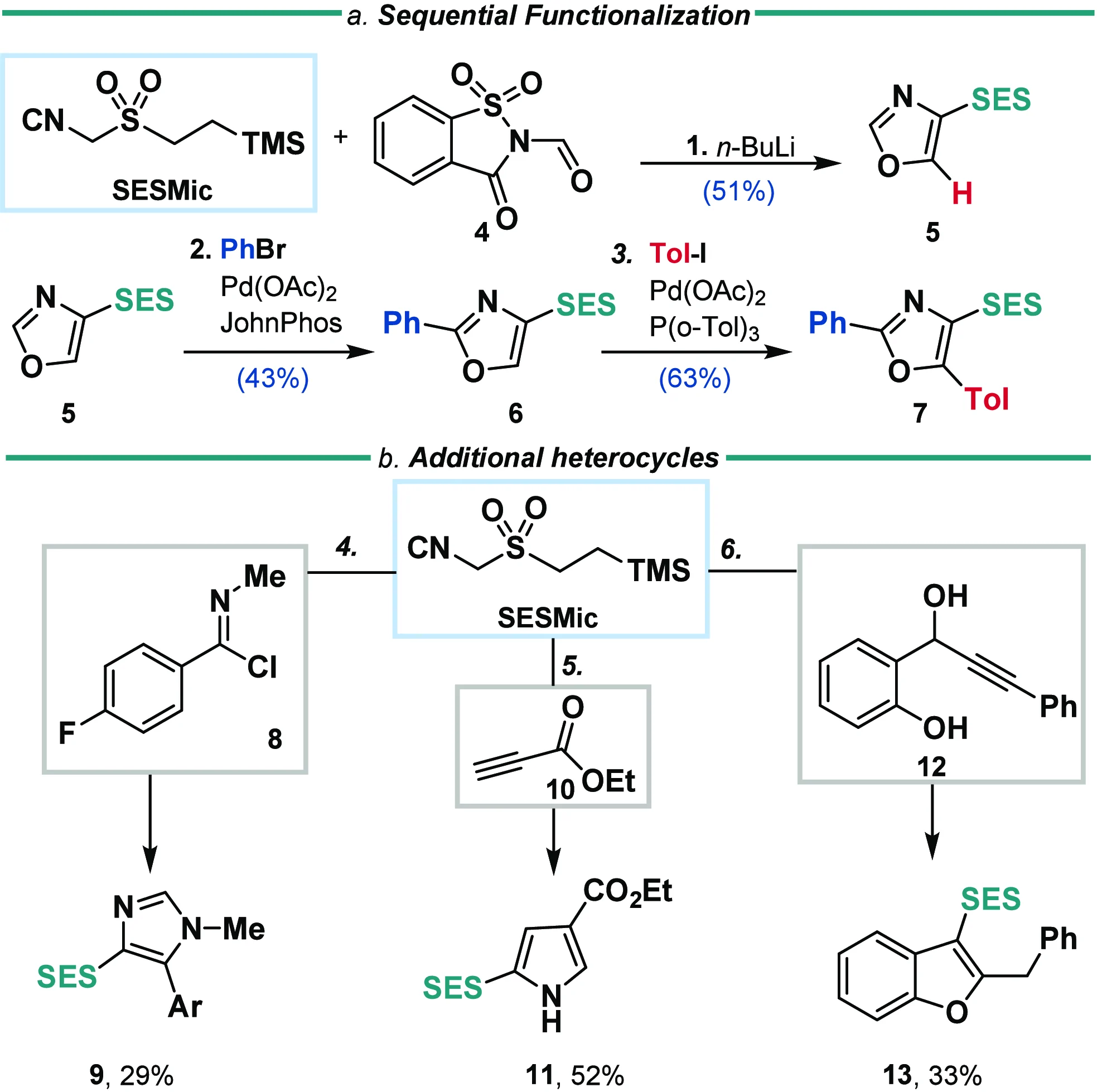
(a) Sequential functionalization of 4-SES-oxazole and (b). synthesis of additional heterocycles. Reagents and conditions: (1) SESMicI (1.1 equiv), *n*-BuLi (1.2 equiv), THF, HMPA, −78 °C to rt, 16 h, 51% yield; (2) PhBr (1 equiv), Pd­(OAc)_2_ (0.05 equiv), Cs_2_CO_3_ (2 equiv), JohnPhos (0.1 equiv), dioxane, 110 °C, 18 h, 43% yield; (3) 4-iodotoluene (1.2 equiv), Pd­(OAc)_2_ (0.2 equiv), Cs_2_CO_3_ (2 equiv), P­(o-Tol)_3_ (0.2 equiv), dioxane, 110 °C, 18 h, 63% yield; (4) (i) **8** (1 equiv), SOCl_2_ (1 M), 65 °C, 16 h, (ii) SESMic (1.5 equiv) *n*-BuLi (1.6 equiv), THF, 16 h, −78 °C to rt, 29% yield; (5) KO^t^Bu, (5.5 equiv), **10** (1 equiv), SESMic (1 equiv), THF, 1 h, rt, 52% yield; (6) **12** (1 equiv), H_2_O (2 equiv), SESMic (1.5 equiv), Ag_2_CO_3_ (0.1 equiv), 1,4-dioxane, 100 °C, 1 h, 33% yield.

SESMic can react with imidoyl chlorides (**8**) to yield imidazoles (**9**, see Figure 3b). SESMic also affords pyrroles (**11**) through the addition to ethyl propiolate (**10**) as well as benzofurans through a silver-mediated addition to hydroxy alkynes such as **12**. The SES functionality can be deprotected and converted to the SF (**15a, 15c, 15l**) with TBAF and subsequent addition of Selectfluor (Figure 4a). Yields for this transformation were moderate for both aryl and alkyl groups and were in line with our previous yields for this deprotection.^27^ The corresponding sulfinate intermediate can also be converted to alkyl sulfones through alkylation and can be elaborated into the corresponding sulfonamide in a one-pot oxidation-addition sequence.

**Figure 4:**
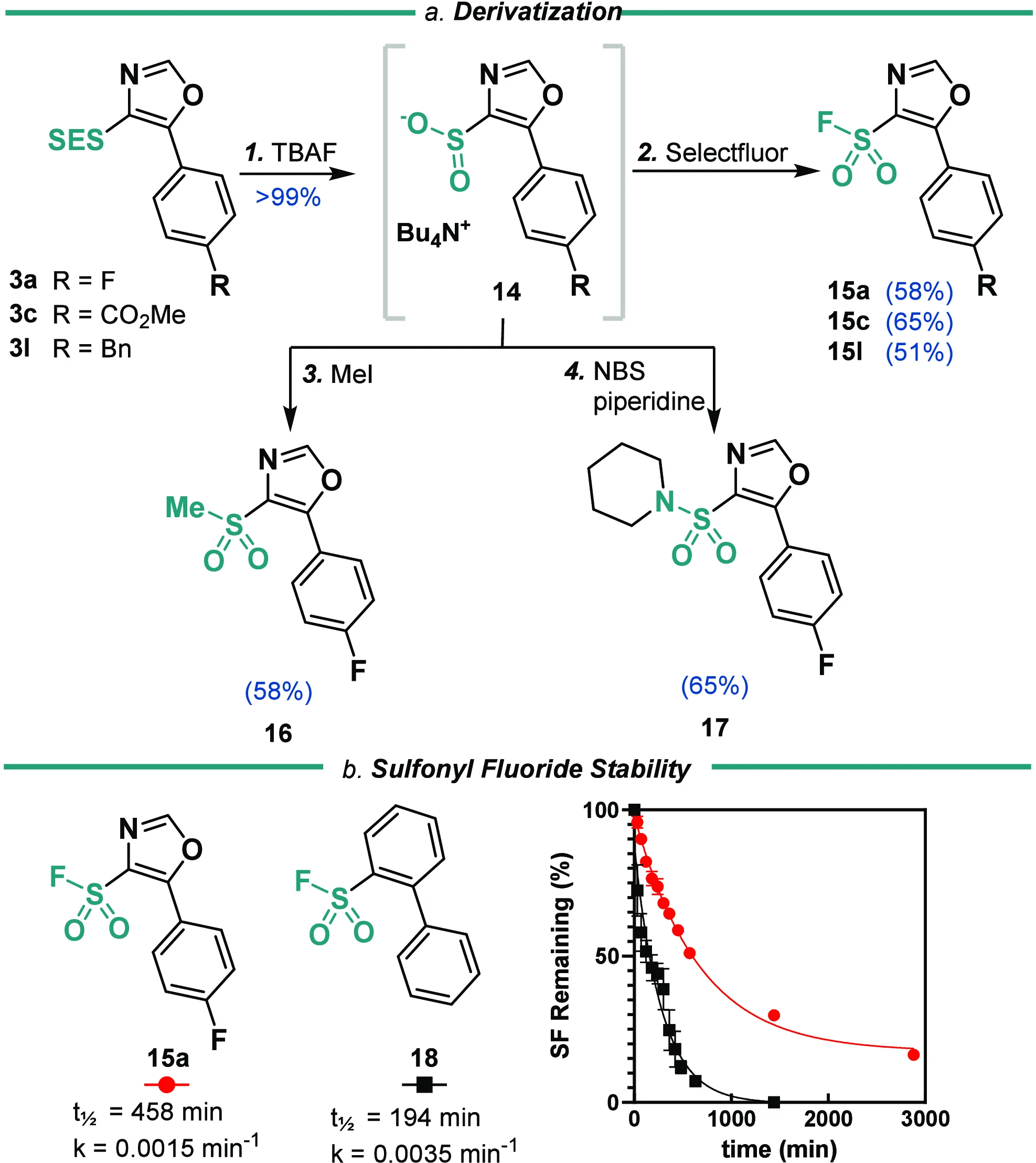
(a) Derivatization of SES-oxazoles. Reagents and conditions: (1) TBAF (2 equiv), CH_3_CN, 2 h, 70 °C >99% yield; (2) Selectfluor (2 equiv), CH_3_CN, 2 h, rt, 58% yield; (3) MeI (4 equiv), CH_3_CN, 2 h, rt, 58% yield; (4) NBS (1.5 equiv), piperidine (10 equiv) CH_3_CN, 16 h, 0 °C to rt, 65% yield. All yields shown over two steps from **3a**. (b) Aqueous sulfonyl fluoride stability testing: percent of **15a** (●) or **18** (■) remaining in pH 7.5 PBS (*n* = 3) at various time points as determined by ^19^F NMR with standard deviation shown. Half-life (*t*_1/2_) and rate of decay (*k*) were calculated by fitting to a one phase decay model.

Next, we proceeded to test the hydrolytic stability of **15a** in relation to its aromatic congener **18** (Figure 4b). SF **15a** proved to be 2.3 times more stable than **18** (*t*_1/2_ of 458 and 194 min for **15a** and **18**, respectively), thus confirming the initial expectation that 4-substituted SFs could be more useful in drug discovery applications. It is worth noting that in addition to the electron rich nature of the 4-position, the increased steric encumbrance of the now closer aryl group could also lead to the decreased hydrolytic reactivity compared to **18**. This is in line with reported reactivities of these types of heterocycles.^8,38^

In summary, we have developed a reagent, SESMic, that provides access to numerous heterocyclic sulfonyl-containing compounds. The SES group can be deprotected and converted into a variety of sulfur­(VI) products, while iterative coupling of SES oxazole **5** provides additional modularity to these approaches. Overall, this reagent adds to the toolbox available for the synthesis and diversification of sulfur­(VI)-containing azoles and 5-membered heteroarenes.

## Supplementary Material



## Data Availability

The data underlying this study are available in the published article and its Supporting Information.

## Notes

Experimental procedures, compound characterization, and supporting figures (PDF)

## Abbreviations

TosMic: *p*-tolylsulfonylmethyl isocyanide
SESMic: (2-((isocyanomethyl)­sulfonyl)­ethyl)­trimethylsilane
HMNPC: (2*S*,4*R*)-4-Hydroxy-N-(2-methylnaphthalen-1-yl)­pyrrolidine-2-carboxamide
CDI: 1,1′-carbonyl diimidazole
