# Supplementary material for: SESMic: A Reagent for the Synthesis of 5‑Membered Heteroaryl Masked Sulfinates
Source: Org Lett. 2026 Aug 5;28(33):10543–7. doi: 10.1021/acs.orglett.6c02962 (PMC13505289; doi:10.1021/acs.orglett.6c02962)

Supporting Information

SESMic: A reagent for the synthesis of 5-membered heteroaryl masked sulfinates

Yumeng Chi, Geoffrey H. Chan, and Matthew J. Moschitto,*[a]

Department of Medicinal Chemistry
Ernest Mario School of Pharmacy, Rutgers, the State University of New Jersey
163 Frelinghuysen Road, Piscataway New Jersey, USA

Table of Contents

General Information 3
Synthesis of SESMic 4
Oxazole Synthesis 5
Oxazole Functionalization 13
Additional Heterocycles 14
Derivatization 16
Stability Testing 18
Spectra 20

General Information

All chemicals were purchased from Ambeed, Sigma Aldrich or ThermoFisher Scientific and used without purification. Anhydrous solvents (THF, CH_2Cl_2, DMF, CH_3CN) were purified before use by passing through a column composed of activated alumina and a supported copper redox catalyst. ACS grade MeOH was purchased from Fisher Chemicals and used without purification. Aluminum oxide (activated, Brockmann I, standard grade, neutral) was purchased from Sigma Aldrich. Yields refer to chromatographically and spectroscopically (^{1}H NMR) homogeneous material unless otherwise noted. Analytical thin-layer chromatography (TLC) was performed using Merck Silica Gel 60 Å F-254 precoated plates (0.25 mm thickness), and components were visualized by ultraviolet light (254 nm) and/or by staining with $KMnO_4$ or phosphomolybdic acid. Flash column chromatography was performed on a Teledyne CombiFlash NextGen 300 automated flash purification system with various Teledyne cartridges (4-80 g, 40-63 µm, 60 Å). Purifications were performed with hexanes and ethyl acetate unless otherwise noted. ^{1}H and ^{13}C NMR spectra were recorded on a Bruker Advance-III spectrometer at 400 MHz and 101 MHz or on a Bruker Advance Neo at 500 MHz and 126 MHz, respectively, in $CDCl_3$, methanol-d_4, benzene-d_6, or DMSO-d_6. Chemical shifts were reported in ppm; multiplicities are indicated by s = singlet, d = doublet, t = triplet, q = quartet, sep = septet, dd = doublet of doublet, dt = doublet of triplet, m = multiplet, br = broad resonance. Coupling constants '*J*' were reported in Hz. High-resolution mass spectral data were obtained on a Waters SYNAPT XS high resolution mass spectrometer using electron spray ionization (ESI) in the positive ion mode.

Synthesis of SESMic

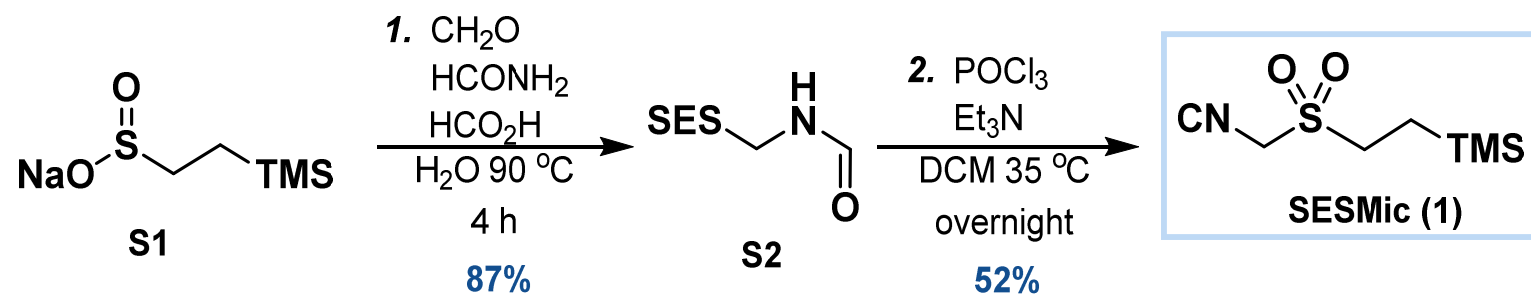

Synthesis of N-(((2-(trimethylsilyl)ethyl)sulfonyl)methyl)formamide (S2)

A 25 mL round-bottomed flask fitted with a reflux condenser was charged with sodium trimethylsilylethyl sulfinate (**S1,** 1.00 g, 5.3 mmol), formaldehyde (1.30 mL, 15.9 mmol, 3 equiv, 37% in water), formamide (2.10 mL, 53.1 mmol, 10 equiv), formic acid (0.70 mL, 18.6 mmol, 3.5 equiv) and water (2.7 mL). The reaction mixture was stirred at 90 °C in an oil bath for 4 h. After cooling to room temperature, water (2.7 mL) was added, and the aqueous layer was extracted with ethyl acetate (10 mL). The organic layer was washed with water, 1 M HCl, and brine. The solution was dried (Na_2SO_4), filtered, and concentrated *in vacuo* to yield a colorless oil. The reaction mixture was purified by flash chromatography with hexanes:ethyl acetate (gradient from 100:0 to 80:20 over 16 min) to afford product **S2** (1.03 g, 4.6 mmol, 87% yield) as a sticky oil. **^{1}H NMR** (400 MHz, $CDCl_3$) δ 8.23 (s, 1H), 6.5 (s, 1H), 4.52 (d, *J* = 6.9 Hz, 2H), 2.90 (m, 2H), 1.07 (m, 2H), 0.00 (s, 9H). **^{13}C{^{1}H} NMR** (101 MHz, $CDCl_3$) δ 160.6, 55.0, 48.5, 8.3, -1.9; **HRMS** (ESI-TOF) m/z: $[M+H]^+$ calc'd for $C_7H_{17}NO_3SSi$: 224.0776; found: 224.0767.

(2-((isocyanomethyl)sulfonyl)ethyl)trimethylsilane (SESMic, 1)

A 25 mL sealed tube was charged with **S2** (500 mg, 2.2 mmol), $POCl_3$ (0.4 mL, 4.4 mmol, 2 equiv), and THF (3.3 mL). Et_3N (1.8 mL, 13.2 mmol, 6 equiv) was added dropwise to the reaction mixture at 0 °C. After addition, the mixture was stirred at 23 °C for 1 h. Water was slowly added at 0 °C, and the solution was extracted with ethyl acetate (10 mL X 3). The organic layer was washed with brine, dried (Na_2SO_4), filtered, and concentrated *in vacuo*. The reaction mixture was purified by activated alumina (neutral) flash chromatography with hexanes/ethyl acetate (gradient from 0-20% ethyl acetate over 10 min) to afford the SESMic **1** (241 mg, 1.2 mmol, 53% yield) as a noncrystalline amorphous solid. **^{1}H NMR** (400 MHz, $CDCl_3$) δ 4.55 (s, 2H), 3.21 – 3.12 (m, 2H), 1.11 – 1.02 (m, 2H), 0.11 (s, 9H). **^{13}C{^{1}H} NMR** (101 MHz, C_6D_6) δ 167.8, 56.4, 47.3, 7.8, -2.4. **HRMS** (ESI-TOF) m/z: $[M+H+MeOH]^+$ calc'd for $C_8H_{20}NO_3SSi$: 238.0933; found: 238.0928.

Note: At higher concentrations in $CDCl_3$, SESMic will form a gel. This does not occur in C_6D_6. SESMic forms a more crystalline solid at -20 °C.

Oxazole Synthesis

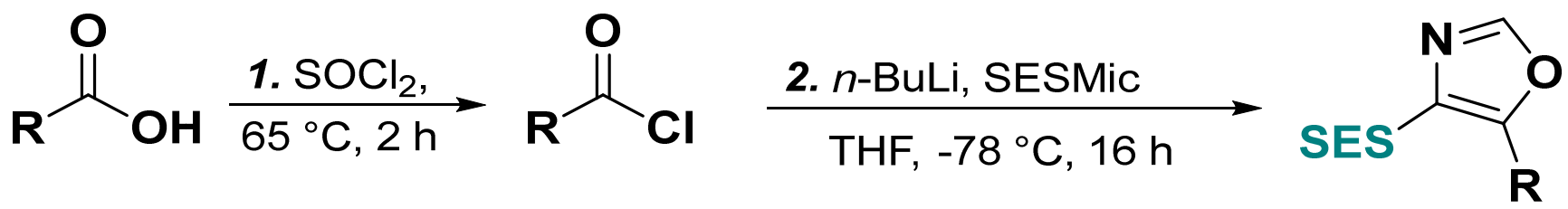

General Procedure A

To a 1-dram vial containing the corresponding carboxylic acid (1 equiv) was added $SOCl_2$ (1 M). The reaction was stirred at 65 °C for 2 h. After cooling to room temperature, the reaction mixture was concentrated and used directly in the next step without purification.

A 2-dram vial was charged with SESMic (1.1 equiv) and THF (0.2 M) under N_2. A solution of *n*-BuLi (1.2 equiv, 1.6 M in hexanes) was added slowly at -78 °C. The reaction mixture was stirred at -78 °C for 15 min before the acyl chloride (1 M in THF) was added dropwise. The reaction mixture was warmed to 0 °C and then stirred at room temperature for 16 h. After the reaction was finished, water was added, and the aqueous layer was extracted with ethyl acetate. The organic layer was washed with 1 M HCl and brine. The solution was dried (Na_2SO_4), filtered, and concentrated *in vacuo*. The reaction mixture was purified by flash chromatography (hexanes:ethyl acetate gradient from 0-25% ethyl acetate over 10-15 min unless otherwise noted) with hexanes/ethyl acetate to afford the product.

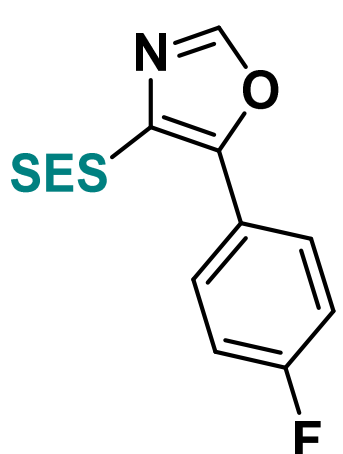

3a

5-(4-fluorophenyl)-4-((2-(trimethylsilyl)ethyl)sulfonyl)oxazole (3a)

Small scale: According to General Procedure A, 4-fluorobenzoic acid (30 mg, 0.21 mmol) and $SOCl_2$ (0.21 mL) yielded the corresponding benzoyl chloride, which was reacted with *n*-BuLi (0.16 mL, 0.26 mmol) and SESMic (49 mg, 0.24 mmol) to yield **3a** (42 mg, 0.13 mmol, 60% yield) as a white solid.

Large scale: A 25 mL round-bottom flask was charged with SESMic (500 mg, 2.42 mmol, 1.1 equiv) and purged by three vacuum–nitrogen refill cycles. THF (12 mL, 0.2 M) was then added via syringe under a nitrogen atmosphere. A solution of *n*-BuLi (1.8 mL, 2.91 mmol, 1.2 equiv, 1.6 M in hexanes) was added slowly at −78 °C, during which the reaction mixture turned dark. The mixture was stirred at −78 °C for 15 min before a solution of 4-fluorobenzoyl chloride (0.26 mL, 2.20 mmol, 1.0 equiv) in THF (0.26 mL) was added dropwise. The reaction mixture was warmed to 0 °C, then stirred at room temperature for 16 h. Upon completion of the reaction, water was added, and the aqueous layer was extracted with ethyl acetate. The combined organic layers were washed with 1 M HCl and brine, dried over Na_2SO_4, filtered, and concentrated *in vacuo*. The crude

product was purified by flash chromatography (hexanes/ethyl acetate 0–25%, v/v) to afford the desired product as a white solid (300 mg, 0.91 mmol, 42%).

^{1}H NMR (400 MHz, $CDCl_3$) δ 8.03 – 7.94 (m, 3H), 7.19 (t, *J* = 8.6 Hz, 2H), 3.35 – 3.26 (m, 2H), 1.03 – 0.94 (m, 2H), 0.04 (s, 9H). **^{13}C{^{1}H} NMR** (101 MHz, $CDCl_3$) δ 166.3 (d, *J* = 253.0 Hz), 154.8, 151.2, 135.2, 133.2 (d, *J* = 8.8 Hz), 123.6, 118.0 (d, *J* = 22.1 Hz), 52.8, 10.4, 0.0. **^{19}F{^{1}H, ^{13}C} NMR** (376 MHz, $CDCl_3$) δ -107.94. **HRMS** (ESI-TOF) m/z: $[M+H]^+$ calc'd for $C_{14}H_{19}FNO_3SSi$: 328.0839; found: 328.0842. **mp** 110-111 °C.

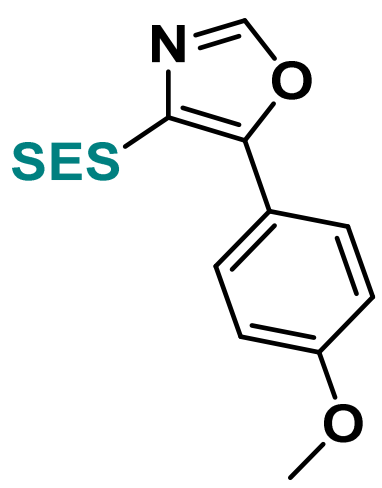

3b

5-(4-methoxyphenyl)-4-((2-(trimethylsilyl)ethyl)sulfonyl)oxazole (3b)

According to General Procedure A, 4-methoxybenzoic acid (30 mg, 0.20 mmol) and $SOCl_2$ (0.2 mL) yielded the corresponding benzoyl chloride, which was reacted with *n*-BuLi (0.15 mL, 0.24 mmol) and SESMic (46 mg, 0.22 mmol) to yield **3b** (16 mg, 0.046 mmol, 23% yield) as a white solid.

^{1}H NMR (400 MHz, $CDCl_3$) δ 7.93 (m, 3H), 7.00 (d, *J* = 8.9 Hz, 2H), 3.86 (s, 3H), 3.32 – 3.23 (m, 2H), 1.02 – 0.93 (m, 2H), 0.03 (s, 9H). **^{13}C{^{1}H} NMR** (101 MHz, $CDCl_3$) δ 163.7, 155.9, 150.7, 133.8, 132.5, 119.7, 116.2, 57.4, 52.9, 10.4, 0.0. **HRMS** (ESI-TOF) m/z: $[M+H]^+$ calc'd for $C_{15}H_{22}NO_4SSi$: 340.1039; found: 340.1049. **mp** 55-58 °C.

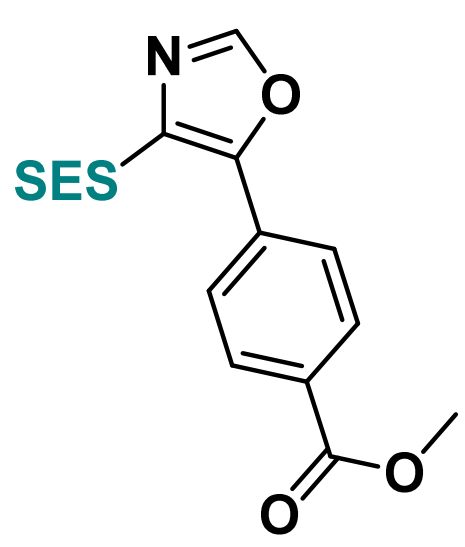

3c

Methyl 4-(4-((2-(trimethylsilyl)ethyl)sulfonyl)oxazol-5-yl)benzoate (3c)

According to General Procedure A, 4-(methoxycarbonyl)benzoic acid (30 mg, 0.17 mmol) and $SOCl_2$ (0.17 mL) yielded the corresponding benzoyl chloride, which was reacted with *n*-BuLi (0.13 mL, 0.20 mmol) and SESMic (38 mg, 0.18 mmol) to yield **3c** (22 mg, 0.060 mmol, 36% yield) as a white solid.

^{1}H NMR (400 MHz, $CDCl_3$) δ 8.15 (d, *J* = 8.5 Hz, 2H), 8.07 (d, *J* = 8.5 Hz, 2H), 8.02 (s, 1H), 3.95 (s, 3H), 3.37 – 3.28 (m, 2H), 1.04 – 0.93 (m, 2H), 0.04 (d, *J* = 1.0 Hz, 9H). **^{13}C{^{1}H} NMR** (101 MHz, $CDCl_3$) δ 168.2, 154.4, 151.7, 136.9, 134.1, 131.8, 131.2, 130.8, 54.4, 52.9, 10.4, 0.0. **HRMS** (ESI-TOF) m/z: $[M+H]^+$ calc'd for $C_{16}H_{22}NO_5SSi$: 368.0988; found: 368.0990. **mp** 55-58 °C.

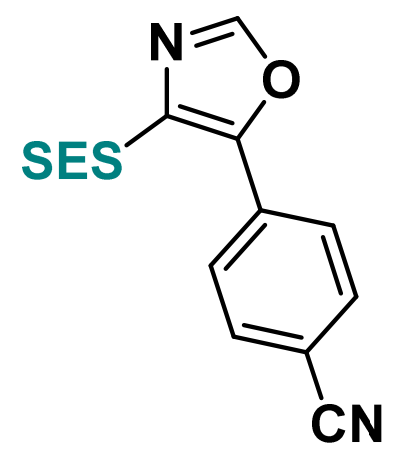

3d

4-(4-((2-(trimethylsilyl)ethyl)sulfonyl)oxazol-5-yl)benzonitrile (3d)

According to General Procedure A, 4-cyanobenzoic acid (30 mg, 0.20 mmol) and $SOCl_2$ (0.20 mL) yielded the corresponding benzoyl chloride, which was reacted with *n*-BuLi (0.18 mL, 0.29 mmol) and SESMic (46 mg, 0.22 mmol) to yield **3d** (28 mg, 0.085 mmol, 42% yield) as a white solid.
^{1}H NMR (400 MHz, $CDCl_3$) δ 8.13 (d, *J* = 8.5 Hz, 2H), 8.04 (s, 1H), 7.78 (d, *J* = 8.5 Hz, 2H), 3.39 – 3.30 (m, 2H), 1.03 – 0.94 (m, 2H), 0.05 (s, 9H). **^{13}C{^{1}H} NMR** (101 MHz, $CDCl_3$) δ 153.2, 152.0, 137.7, 134.4, 131.3, 131.3, 120.0, 116.5, 52.8, 10.4, 0.0. **HRMS** (ESI-TOF) m/z: $[M+Na]^+$ calc'd for $C_{15}H_{18}N_2O_3SSiNa$: 357.0705; found: 357.0699. **mp** 128-131 °C.

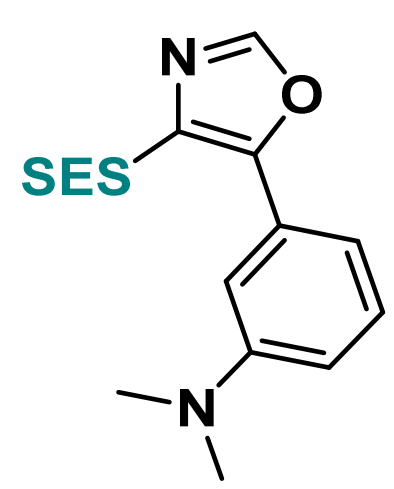

3e

***N,N*-dimethyl-3-(4-((2-(trimethylsilyl)ethyl)sulfonyl)oxazol-5-yl)aniline (3e)**

A 2-dram vial was charged with SESMic (32 mg, 0.15 mmol) and THF (0.70 mL) under N_2. A solution of *n*-BuLi (0.10 mL, 0.18 mmol) was added slowly at -78 °C. The reaction mixture was stirred at -78 °C for 15 min before (3-(dimethylamino)phenyl)(1H-imidazol-1-yl)methanone[1] (30 mg, 0.14 mmol in 0.14 mL THF) was added dropwise. The reaction was warmed to 0 °C and then stirred at room temperature overnight. After the reaction was finished, water was added, and the aqueous layer was extracted with ethyl acetate. The organic layer was washed with 1 M HCl and brine. The solution was dried (Na_2SO_4), filtered, and concentrated *in vacuo*. The reaction mixture was purified by flash chromatography (hexanes/ethyl acetate, 0-30%) to afford **3e** (21 mg, 0.058 mmol, 42%) as an amorphous solid.

^{1}H NMR (400 MHz, $CDCl_3$) 7.94 (s, 1H), 7.42 (s, 1H), 7.33 (t, *J* = 7.98 Hz, 1H), 7.23 (d, *J* = 8.04 Hz, 1H), 6.84 (d, *J* = 8.26 Hz, 1H), 3.33 – 3.24 (m, 2H), 3.01 (s, 6H), 1.04 – 0.95 (m, 2H), 0.02 (s, 9H). **^{13}C{^{1}H} NMR** (101 MHz, $CDCl_3$) δ 156.5, 152.5, 150.8, 135.0, 131.4, 127.9, 118.3, 116.9, 114.9, 52.9, 42.5, 10.5, 0.0. **HRMS** (ESI-TOF) m/z: $[M+H]^+$ calc'd for $C_{16}H_{25}N_2O_3SSi$: 353.1355; found: 353.1371.

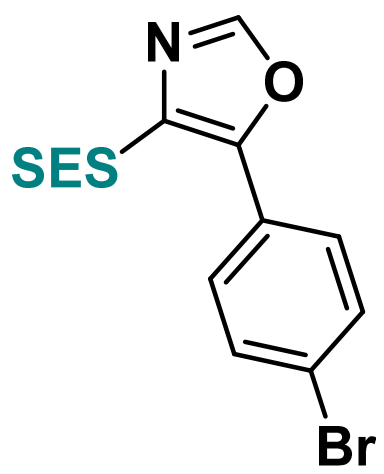

3f

5-(4-bromophenyl)-4-((2-(trimethylsilyl)ethyl)sulfonyl)oxazole (3f)

According to General Procedure A, 4-bromobenzoic acid (30 mg, 0.15 mmol) and $SOCl_2$ (0.15 mL) yielded the corresponding benzoyl chloride, which was reacted with *n*-BuLi (0.11 mL, 0.179 mmol) and SESMic (33.9 mg, 0.18 mmol) to yield **3f** (34.2 mg, 0.088 mmol, 59 % yield) as a pale yellow solid.

^{1}H NMR (400 MHz, $CDCl_3$) δ 7.97 (s, 1H), 7.85 (d, *J* = 8.4 Hz, 2H), 7.63 (d, *J* = 8.4 Hz, 2H), 3.35 – 3.26 (m, 2H), 1.02 – 0.93 (m, 2H), 0.04 (s, 9H). **^{13}C{^{1}H} NMR** (101 MHz, $CDCl_3$) δ 154.6, 151.8, 135.8, 134.5, 132.3, 127.8, 126.2, 52.9, 10.4, 0.3. **HRMS** (ESI-TOF) m/z: $[M+H]^+$ calc'd for $C_{14}H_{19}BrNO_3SSi$: 388.0038; found: 388.0032. **mp** 118-119 °C.

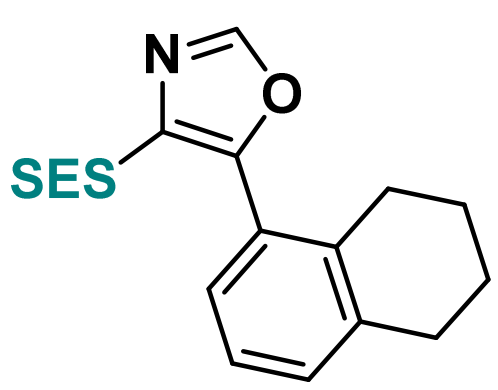

3g

5-(5,6,7,8-tetrahydronaphthalen-1-yl)-4-((2-(trimethylsilyl)ethyl)sulfonyl)oxazole (3g)

According to General Procedure A, 5,6,7,8-tetrahydronaphthalene-1-carboxylic acid (30 mg, 0.17 mmol) and $SOCl_2$ (0.17 mL) yielded the corresponding benzoyl chloride, which was reacted with *n*-BuLi (0.13 mL, 0.20 mmol) and SESMic (38.6 mg, 0.19 mmol) to yield **3g** (17.3 mg, 0.058 mmol, 28% yield) as an amorphous solid.

^{1}H NMR (400 MHz, $CDCl_3$) δ 8.00 (s, 1H), 7.31 – 7.15 (m, 3H), 3.21 – 3.12 (m, 2H), 2.83 (t, *J* = 6.1 Hz, 2H), 2.61 (t, *J* = 6.1 Hz, 2H), 1.79 (tdd, *J* = 17.1, 8.3, 5.2 Hz, 4H), 0.99 – 0.87 (m, 2H), 0.02 (s, 9H). **^{13}C{^{1}H} NMR** (101 MHz, $CDCl_3$) δ 157.0, 152.1, 140.0, 139.2, 136.6, 134.4, 131.2,

127.2, 126.8, 53.0, 31.7, 29.5, 24.8, 24.6, 10.5, 0.0. **HRMS** (ESI-TOF) m/z: $[M+Na]^+$ calc'd for $C_{18}H_{25}NO_3SSiNa$: 386.1222; found: 386.1242.

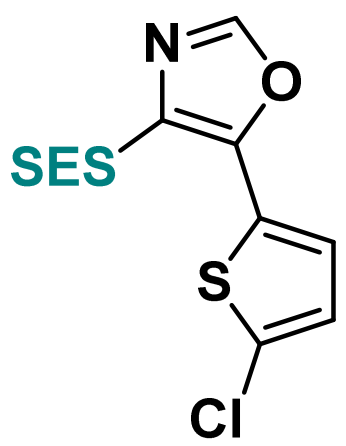

3h

5-(5-chlorothiophen-2-yl)-4-((2-(trimethylsilyl)ethyl)sulfonyl)oxazole (3h)

According to General Procedure A, 5-chlorothiophene-2-carboxylic acid (30 mg, 0.19 mmol) and $SOCl_2$ (0.19 mL) yielded the corresponding acid chloride, which was reacted with *n*-BuLi (0.14 mL, 0.22 mmol) and SESMic (42 mg, 0.20 mmol) to yield **3h** (41 mg, 0.12 mmol, 63% yield) as an amorphous solid. **^{1}H NMR** (400 MHz, $CDCl_3$) δ 7.87 (s, 1H), 7.79 (d, J = 4.1 Hz, 1H), 6.98 (d, J = 4.1 Hz, 1H), 3.31 – 3.22 (m, 2H), 1.03 – 0.94 (m, 2H), 0.04 (s, 9H). **^{13}C{^{1}H} NMR** (126 MHz, $CDCl_3$) δ 148.7, 148.3, 136.1, 131.1, 131.0, 127.5, 124.8, 51.2, 8.6, -1.9. **HRMS** (ESI-TOF) m/z: $[M+Na]^+$ calc'd for $C_{12}H_{16}ClNO_3S_2SiNa$: 371.9927; found: 371.9914.

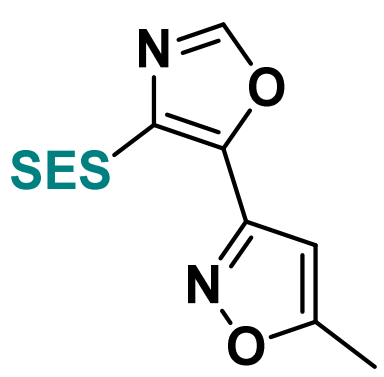

3i

5-methyl-3-(4-((2-(trimethylsilyl)ethyl)sulfonyl)oxazol-5-yl)isoxazole (3i)

According to General Procedure A, 5-methylisoxazole-3-carboxylic acid (30 mg, 0.24 mmol) and $SOCl_2$ (0.24 mL) yielded the corresponding acid chloride, which was reacted with *n*-BuLi (0.18 mL, 0.28 mmol) and SESMic (54 mg, 0.26 mmol) to yield **3i** (22 mg, 0.071 mmol, 30% yield) as yellow solid.

^{1}H NMR (400 MHz, $CDCl_3$) δ 9.78 (s, 1H), 6.57 (s, 1H), 3.03 – 2.94 (m, 2H), 2.54 (s, 3H), 1.16 – 1.06 (m, 2H), 0.08 (s, J = 1.4 Hz, 9H). **^{13}C{^{1}H} NMR** (126 MHz, $CDCl_3$) δ 171.2, 151.2, 151.0, 144.2, 137.0, 102.9, 51.3, 11.9, 8.4, -2.1. **HRMS** (ESI-TOF) m/z: $[M+H]^+$ calc'd for $C_{12}H_{18}N_2O_4SSi$: 315.0835; found: 315.0838. **mp** 151-152 °C.

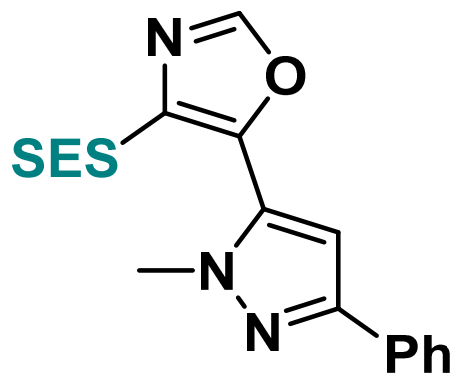

3j

5-(1-methyl-3-phenyl-1H-pyrazol-5-yl)-4-((2-(trimethylsilyl)ethyl)sulfonyl)oxazole (3j)

According to General Procedure A, 1-methyl-3-phenyl-1H-pyrazole-5-carboxylic acid (30 mg, 0.15 mmol) and $SOCl_2$ (0.15 mL) yielded the corresponding acid chloride, which was reacted with *n*-BuLi (0.11 mL, 0.18 mmol) and SESMic (33 mg, 0.16 mmol) to yield **3j** (28 mg, 0.071 mmol, 48% yield) as a yellow oil. Flash chromatography (hexanes:ethyl acetate gradient 0-40% over 15 min).

^{1}H NMR (400 MHz, $CDCl_3$) δ 8.07 (s, 1H), 7.81 (d, *J* = 8.8 Hz, 2H), 7.41 (t, *J* = 7.5 Hz, 2H), 7.32 (t, *J* = 7.3 Hz, 1H), 7.25 (s, 1H), 4.07 (s, 3H), 3.33 – 3.24 (m, 2H), 1.04 – 0.94 (m, 2H), 0.05 (s, 9H). **^{13}C{^{1}H} NMR** (101 MHz, $CDCl_3$) δ 153.1, 152.2, 146.0, 137.6, 134.4, 130.7, 130.1, 127.7, 127.6, 110.6, 52.9, 41.2, 10.3, 0.0. **HRMS** (ESI-TOF) m/z: $[M+H]^+$ calc'd for $C_{18}H_{24}N_3O_3SSi$: 390.1307; found: 390.1305.

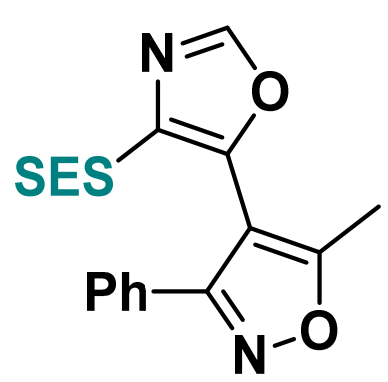

3k

5-methyl-3-phenyl-4-(4-((2-(trimethylsilyl)ethyl)sulfonyl)oxazol-5-yl)isoxazole (3k)

According to General Procedure A, 5-methyl-3-phenylisoxazole-4-carboxylic acid (30 mg, 0.15 mmol) and $SOCl_2$ (0.15 mL) yielded the corresponding acid chloride, which was reacted with *n*-BuLi (0.11 mL, 0.18 mmol) and SESMic (33 mg, 0.16 mmol) to yield **3k** (18 mg, 0.046 mmol, 31% yield) as a yellow solid. Flash chromatography (hexanes:ethyl acetate gradient 0-25% over 15 min).

^{1}H NMR (400 MHz, $CDCl_3$) δ 8.03 (s, 1H), 7.50 – 7.34 (m, 5H), 3.06 – 2.97 (m, 2H), 2.53 (s, 3H), 0.82 – 0.73 (m, 2H), -0.02 (s, 9H). **^{13}C{^{1}H} NMR** (101 MHz, $CDCl_3$) δ 174.7, 163.3, 153.6, 147.4, 139.1, 132.3, 130.9, 130.2, 129.9, 103.7, 52.8, 14.5, 10.4, 0.0. **HRMS** (ESI-TOF) m/z: [M+H]+ calc'd for $C_{18}H_{23}N_2O_4SSi$: 391.1148; found: 391.1162. **mp** 95-98 °C.

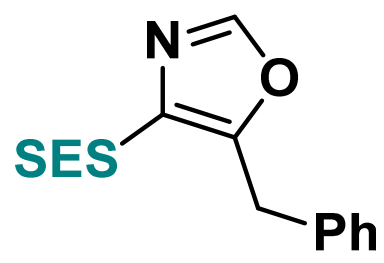

3l

5-benzyl-4-((2-(trimethylsilyl)ethyl)sulfonyl)oxazole (3l)

According to General Procedure A, 2-phenylacetic acid (30 mg, 0.22 mmol) and $SOCl_2$ (0.22 mL) yielded the corresponding acid chloride, which was reacted with *n*-BuLi (0.17 mL, 0.26 mmol) and SESMic (50 mg, 0.24 mmol) to yield **3l** (26 mg, 0.082 mmol, 37% yield) as yellow solid.

^{1}H NMR (400 MHz, $CDCl_3$) δ 7.77 (s, 1H), 7.29-7.14 (m, 5H), 4.33 (s, 2H), 3.13 – 3.05 (m, 2H), 0.88 – 0.79 (m, 2H), -0.04 (s, 9H). **^{13}C{^{1}H} NMR** (101 MHz, $CDCl_3$) δ 156.8, 150.1, 135.5, 132.8, 129.1, 129.0, 127.5, 51.2, 31.4, 8.8, -1.9. **HRMS** (ESI-TOF) m/z: [M+O-H] (Bn oxidized) calc'd for $C_{15}H_{19}NO_4SSi$: 338.0882; found: 338.0891. **mp** 50-52 °C.

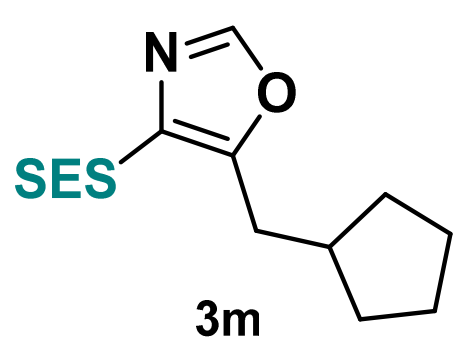

3m

5-(cyclopentylmethyl)-4-((2-(trimethylsilyl)ethyl)sulfonyl)oxazole (3m)

According to General Procedure A, 2-cyclopentylacetic acid (30 mg, 0.23 mmol) and $SOCl_2$ (0.23 mL) yielded the corresponding acid chloride, which was reacted with *n*-BuLi (0.18 mL, 0.281 mmol) and SESMic (53 mg, 0.26 mmol) to yield **3m** (36 mg, 0.12 mmol, 49% yield) as a yellow oil.

^{1}H NMR (400 MHz, $CDCl_3$) δ 7.83 (s, 1H), 3.21 – 3.12 (m, 2H), 3.04 (d, *J* = 7.5 Hz, 2H), 2.24 (p, *J* = 7.7 Hz, 1H), 1.79 – 1.72 (m, 2H), 1.67 (m, 2H), 1.60 (m, 2H), 1.26 (m, 2H), 0.99 – 0.90 (m, 2H), 0.04 (s, 9H). **^{13}C{^{1}H} NMR** (101 MHz, $CDCl_3$) δ 160.5, 151.6, 134.8, 53.0, 41.1, 34.3, 32.8, 26.8, 10.7, 0.0. **HRMS** (ESI-TOF) m/z: $[M+Na]^+$ calc'd for $C_{14}H_{25}NO_3SSiNa$: 338.1222; found: 338.1217.

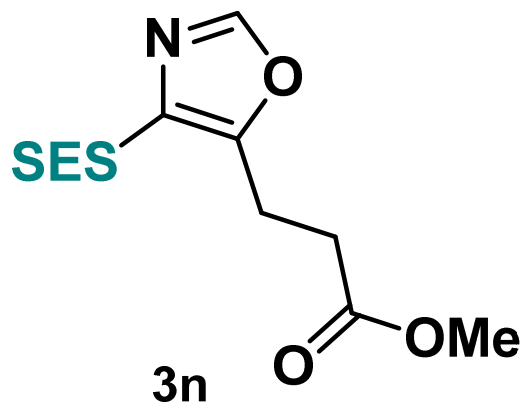

3n

Methyl 3-(4-((2-(trimethylsilyl)ethyl)sulfonyl)oxazol-5-yl)propanoate (3n)

According to General Procedure A, monomethyl succinate (30 mg, 0.23 mmol) and $SOCl_2$ (0.23 mL) yielded the corresponding acid chloride, which was reacted with *n*-BuLi (0.17 mL, 0.27 mmol) and SESMic (51 mg, 0.25 mmol) to yield **3n** (26 mg, 0.082 mmol, 36% yield) as a colorless oil.

^{1}H NMR (400 MHz, CDCl3) δ 7.84 (s, 1H), 3.69 (s, 3H), 3.38 (t, J = 7.3 Hz, 2H), 3.24 – 3.15 (m, 2H), 2.76 (t, J = 7.3 Hz, 2H), 1.02 – 0.93 (m, 2H), 0.05 (s, 9H). **^{13}C{^{1}H} NMR** (101 MHz, $CDCl_3$) δ 173.8, 158.6, 151.8, 135.3, 54.0, 53.0, 33.6, 22.9, 10.5, 0.0. **HRMS** (ESI-TOF) m/z: $[M+Na]^+$ calc'd for $C_{12}H_{21}NO_5SSiNa$: 342.0808; found: 342.0819.

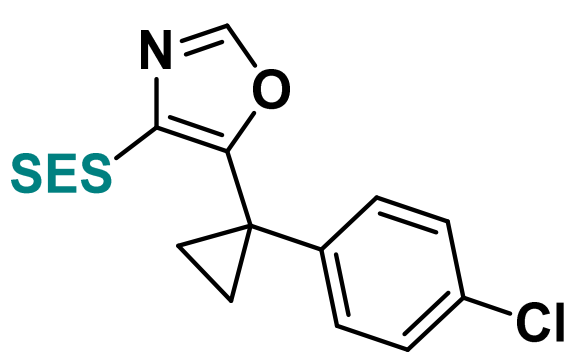

3o

5-(1-(4-chlorophenyl)cyclopropyl)-4-((2-(trimethylsilyl)ethyl)sulfonyl)oxazole (3o)

According to General Procedure A, 1-(4-chlorophenyl)cyclopropane-1-carboxylic acid (30 mg, 0.15 mmol) and $SOCl_2$ (0.15 mL) yielded the corresponding acid chloride, which was reacted with *n*-BuLi (0.11 mL, 0.18 mmol) and SESMic (33 mg, 0.17 mmol) to yield **3o** (28.1 mg, 0.073 mmol, 48% yield) as a yellow oil.

^{1}H NMR (400 MHz, $CDCl_3$) δ 7.80 (s, 1H), 7.39 (d, *J* = 8.5 Hz, 2H), 7.25 (d, *J* = 8.5 Hz, 2H), 3.11 – 3.02 (m, 2H), 1.59 – 1.56 (m, 2H), 1.46 – 1.41 (m, 2H), 0.82 – 0.73 (m, 2H), -0.01 (s, 9H). **^{13}C{^{1}H} NMR** (126 MHz, $CDCl_3$) δ 159.0, 149.4, 139.7, 134.3, 133.4, 130.4, 128.9, 50.9, 20.7, 15.4, 8.5, 0.0. **HRMS** (ESI-TOF) m/z: $[M+H]^+$ calc'd for $C_{17}H_{23}ClNO_3SSi$: 384.0856; found: 384.0858.

Oxazole Functionalization

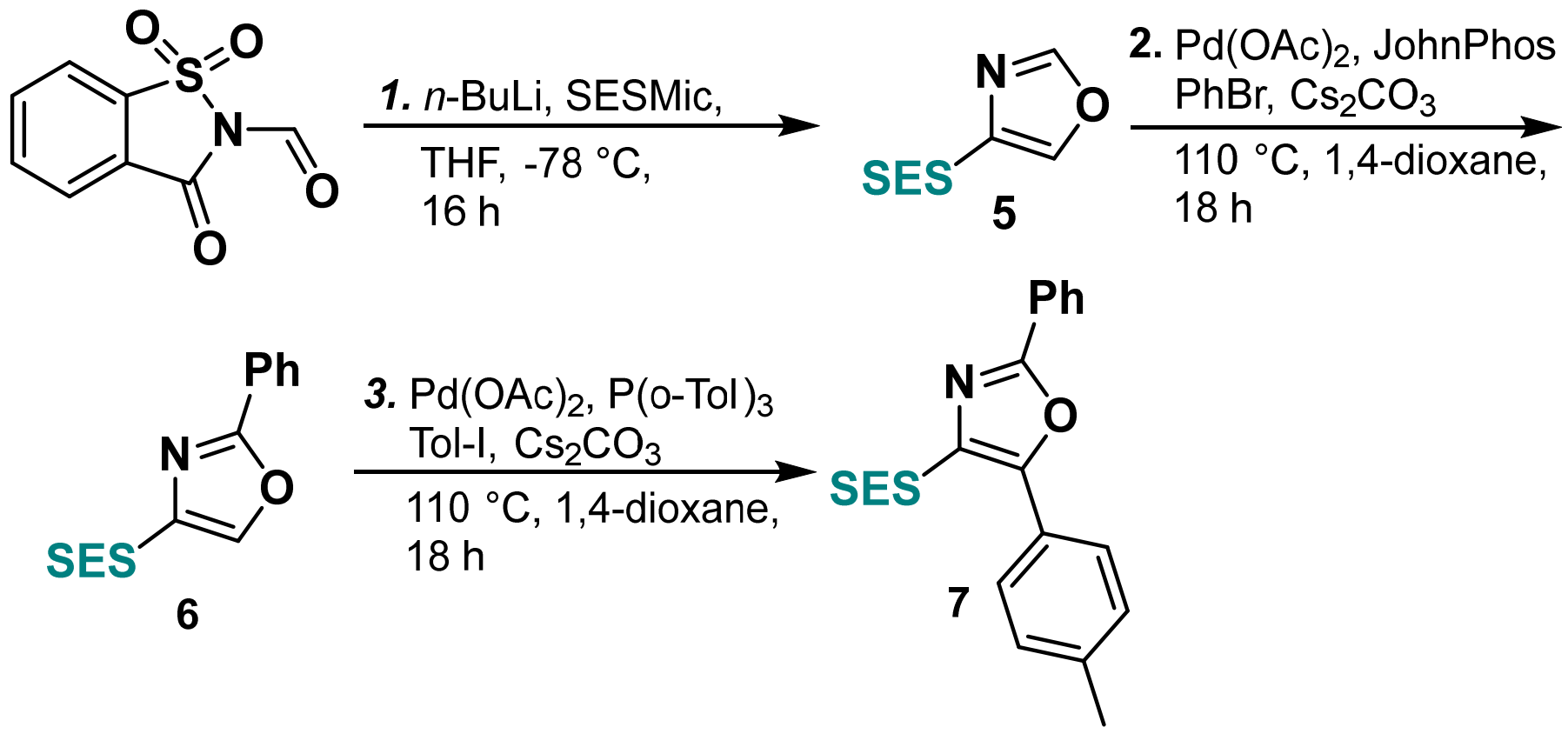

4-((2-(trimethylsilyl)ethyl)sulfonyl)oxazole (5)

A 2-dram vial was charged with SESMic (81 mg, 0.39 mmol, 1.1 equiv) in 1.80 mL THF under N_2. A solution of *n*-BuLi (0.27 mL, 0.43 mmol, 1.2 equiv) was added slowly at -78 °C. After 15 min, a solution of *N*-formyl saccharin (3-oxobenzo[d]isothiazole-2(3H)-carbaldehyde, 75 mg, 0.36 mmol, 1 equiv) in 1.8 mL HMPA was added dropwise. The reaction was warmed to room temperature overnight. After completion of the reaction, workup and purification followed the procedures found in General Procedure A to yield **5** (42 mg, 0.18 mmol, 51% yield) as a white solid. Flash chromatography (hexanes:ethyl acetate 0-20% over 10 min).

^{1}H NMR (400 MHz, $CDCl_3$) δ 8.28 (s, 1H), 8.02 (s, 1H), 3.23 – 3.14 (m, 2H), 1.02 – 0.92 (m, 2H), 0.04 (s, 9H). **^{13}C{^{1}H} NMR** (101 MHz, $CDCl_3$) δ 154.2, 145.4, 142.0, 52.9, 10.7, 0.0. **HRMS** (ESI-TOF) m/z: $[M+Na]^+$ calc'd for $C_8H_{15}NO_3SSiNa$: 256.0440; found: 256.0462. **mp** 58-60 °C.

2-phenyl-4-((2-(trimethylsilyl)ethyl)sulfonyl)oxazole (6)

To a 2-dram vial was added **5** (30 mg, 0.13 mmol, 1 equiv), bromobenzene (13.5 µL, 0.129 mmol, 1 equiv), $Pd(OAc)_2$ (1.4 mg, 0.006 mmol, 0.05 equiv), Cs_2CO_3 (84 mg, 0.26 mmol, 2 equiv), JohnPhos (3.8 mg, 0.013 mmol 0.1 equiv), and dioxane (0.6 mL). The resulting mixture was stirred under N_2 at 110 °C for 18 h. After filtration through Celite and concentration *in vacuo*, the crude product was purified by flash column chromatography (hexanes:ethyl acetate gradient from 0-10% over 10 min) to yield **6** (17 mg, 0.055 mmol, 43% yield) as a white solid.

^{1}H NMR (400 MHz, $CDCl_3$) δ 8.21 (s, 1H), 8.04 (d, *J* = 7.0 Hz, 2H), 7.51 – 7.40 (m, 3H), 3.23 – 3.13 (m, 2H), 1.02 – 0.93 (m, 2H), 0.00 (s, 9H). **^{13}C{^{1}H} NMR** (126 MHz, $CDCl_3$) δ 163.6, 142.9, 141.2, 131.9, 129.2, 127.2, 126.0, 50.9, 8.7, -1.9. **HRMS** (ESI-TOF) m/z: $[M+H]^+$ calc'd for $C_{14}H_{20}NO_3SSi$: 310.0933; found: 310.0930. **mp** 69-73 °C.

2-phenyl-5-(p-tolyl)-4-((2-(trimethylsilyl)ethyl)sulfonyl)oxazole (7)

To a 2-dram vial was added **6** (5 mg, 0.016 mmol, 1 equiv), 4-iodotoluene (4.1 mg, 0.019 mmol, 1.2 equiv), $Pd(OAc)_2$ (0.7 mg, 0.003 mmol, 0.2 equiv), Cs_2CO_3 (10 mg, 0.032 mmol, 2 equiv),

$P(o\text{-}Tol)_3$ (1 mg, 0.003 mmol, 0.2 equiv), and dioxane (0.10 mL). The resulting mixture was stirred under N_2 at 110 °C for 18 h. After filtration through Celite and concentration *in vacuo*, the crude product was purified by flash column chromatography (hexanes:ethyl acetate gradient 0-3% over 15 min) to obtain **7** (4 mg, 0.01 mmol, 63% yield) as a yellow solid.

^{1}H NMR (400 MHz, $CDCl_3$) δ 8.12 (dd, *J* = 6.5, 3.1 Hz, 2H), 7.92 (d, *J* = 7.9 Hz, 2H), 7.51 (m, 3H), 7.32 (d, *J* = 7.9 Hz, 2H), 3.40 – 3.31 (m, 2H), 2.43 (s, 3H), 1.07 – 0.98 (m, 2H), 0.02 (s, 9H). **^{13}C{^{1}H} NMR** (126 MHz, $CDCl_3$) δ 159.9, 153.5, 141.4, 134.3, 131.6, 129.5, 129.1, 128.9, 127.0, 126.2, 123.1, 50.9, 21.7, 8.7, -1.9. **HRMS** (ESI-TOF) m/z: $[M+H]^+$ calc'd for $C_{21}H_{25}NO_3SSi$: 400.1402; found: 400.1404. **mp** 98-99 °C.

Additional Heterocycles

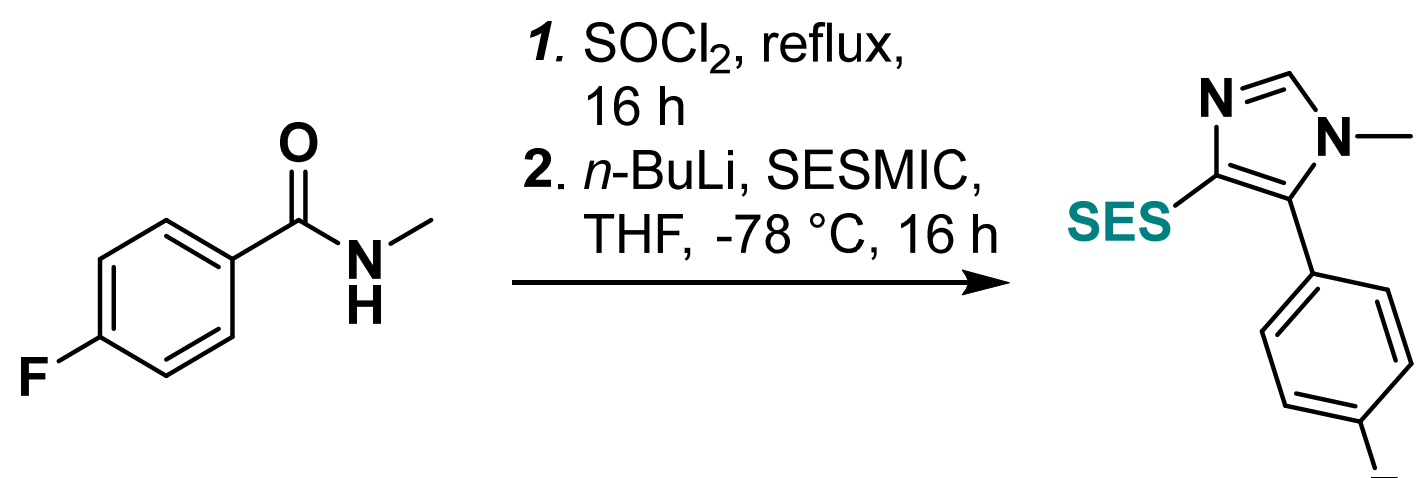

8 9

5-(4-fluorophenyl)-1-methyl-4-((2-(trimethylsilyl)ethyl)sulfonyl)-1H-imidazole (9)

To a 1-dram vial was added 4-fluoro-*N*-methylbenzamide (60 mg, 0.35 mmol, 1 equiv) and $SOCl_2$ (0.35 mL, 1 M). The resulting mixture was refluxed under N_2 overnight then concentration *in vacuo*, the crude product was diluted with anhydrous THF and added to SESMic (109 mg, 0.53 mmol, 1.5 equiv) and *n*-BuLi (0.36 mL, 0.58 mmol, 1.6 equiv) in 1.7 ml THF at -78 °C. The reaction was warmed to room temperature overnight. After completion of the reaction, workup and purification followed the procedures found in General Procedure A (flash chromatography hexanes:ethyl acetate 0-30% over 15 min) to yield **9** (33 mg 0.10 mmol, 29% yield) as an amorphous solid.

^{1}H NMR (500 MHz, $CDCl_3$) δ 7.61 (s, 1H), 7.42 – 7.37 (m, 2H), 7.22 – 7.13 (m, 2H), 3.51 (s, 3H), 3.14 – 3.08 (m, 2H), 0.93 – 0.85 (m, 2H), -0.01 (s, 9H). **^{13}C{^{1}H} NMR** (126 MHz, $CDCl_3$) δ 163.7 (d, *J* = 250.8 Hz), 138.3, 136.4, 135.8, 132.8 (d, *J* = 8.4 Hz), 122.7, 115.9 (d, *J* = 22.0 Hz), 51.2, 32.7, 8.6, -1.9. **^{19}F{^{1}H, ^{13}C} NMR** (376 MHz, $CDCl_3$) δ -110.38. **HRMS** (ESI-TOF) m/z: $[M+H]^+$ calc'd for $C_{15}H_{22}FN_2O_2SSi$: 341.1155; found: 341.1143.

*Note: **9** is unstable and will deuterate at the 2-position if concentrated from $CDCl_3$ or left for long periods of time.

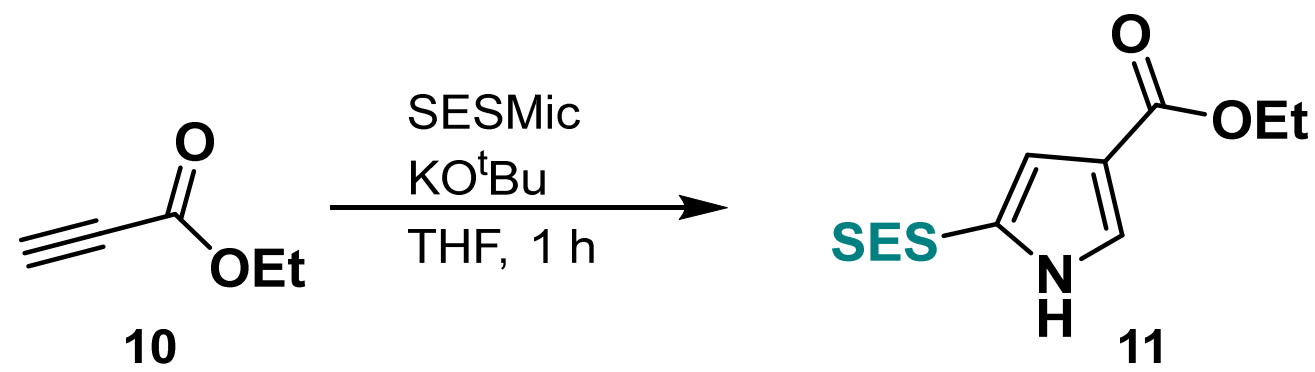

ethyl 5-((2-(trimethylsilyl)ethyl)sulfonyl)-1H-pyrrole-3-carboxylate (11)

A solution of KO^{t}Bu (150 mg, 1.33 mmol, 5.5 equiv) in THF was added to a solution of ethyl propiolate (24.5 µL, 0.24 mmol, 1 equiv) and SESMic (50 mg, 0.24 mmol, 1 equiv) in THF dropwise at room temperature over 15 min. After stirring for 1 h at room temperature, the reaction was quenched with glacial acetic acid (228.7 µL, 4.0 mmol, 16.5 equiv). After concentrating *in vacuo*, workup and purification followed the procedures found in General Procedure A (flash chromatography hexanes:ethyl acetate 0-25% over 10 min) to yield **11** (38 mg 0.13 mmol, 52% yield) as a yellow oil.

^{1}H NMR (400 MHz, $CDCl_3$) δ 9.52 (s, 1H), 7.60 – 7.55 (m, 1H), 4.29 (q, *J* = 7.1 Hz, 2H), 3.08 – 2.99 (m, 2H), 1.33 (t, *J* = 7.25 Hz, 3H), 0.95 – 0.86 (m, 2H), 0.00 (d, *J* = 1.3 Hz, 9H). **^{13}C{^{1}H} NMR** (101 MHz, $CDCl_3$) δ 163.6, 127.6, 127.2, 119.1, 116.6, 60.6, 53.9, 14.5, 9.5, -0.9. **HRMS** (ESI-TOF) m/z: $[M+H]^+$ calc'd for $C_{12}H_{22}NO_4SSi$: 304.1039; found: 304.1022.

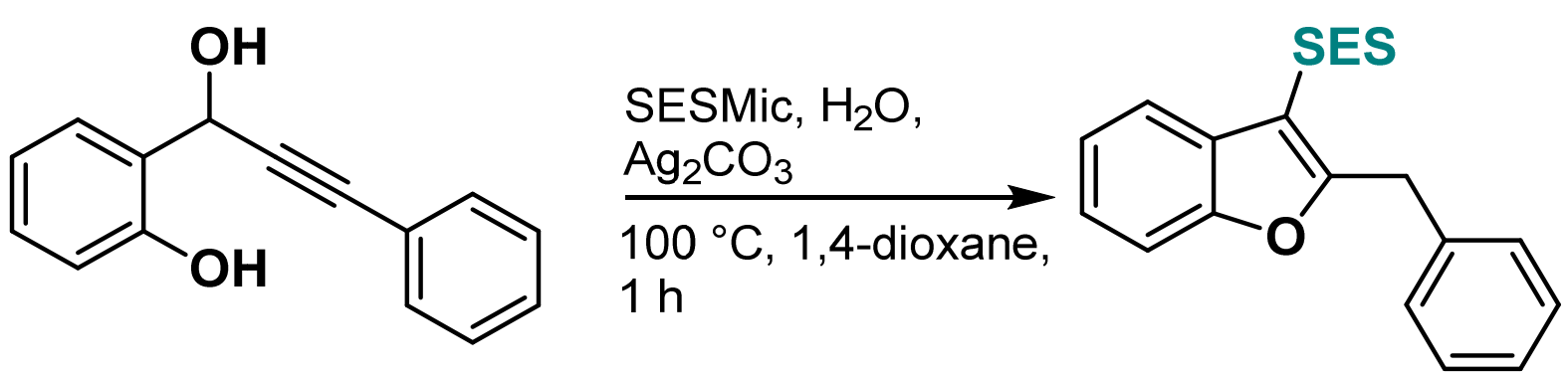

(2-((2-benzylbenzofuran-3-yl)sulfonyl)ethyl)trimethylsilane (13)

A mixture of 2-(1-hydroxy-3-phenylprop-2-yn-1-yl)phenol (**12**) (100 mg, 0.45 mmol 1 equiv), water (16.0 µL, 0.893 mmol, 2 equiv), SESMic (138 mg, 0.67 mmol, 1.5 equiv) and Ag_2CO_3 (12 mg, 0.045 mmol, 0.1 equiv) in 1,4-Dioxane (3.0 mL) was stirred at 100 °C for 1 h. The resulting mixture was concentrated, and the residue was taken up in DCM. The organic layer was washed with brine, dried over $MgSO_4$, and concentrated. The crude product was purified by flash chromatography (hexanes:ethyl acetate gradient from 0-30% over 10-15 min) to yield **13** (55 mg 0.15 mmol, 33% yield) as a yellow oil.

^{1}H NMR (400 MHz, $CDCl_3$) δ 7.87 – 7.80 (m, 1H), 7.52 – 7.48 (m, 1H), 7.41 – 7.22 (m, 7H), 4.51 (s, 2H), 3.02 – 2.93 (m, 2H), 0.94 – 0.85 (m, 2H), -0.06 (s, 9H). **^{13}C{^{1}H} NMR** (101 MHz, $CDCl_3$) δ 162.9, 153.6, 136.2, 129.2, 128.9, 127.3, 125.7, 125.0, 124.7, 120.7, 115.1, 111.7, 53.5, 33.2, 9.1, -1.9. **HRMS** (ESI-TOF) m/z: $[M+Na]^+$ calc'd for $C_{20}H_{24}O_3SSiNa$: 395.1113; found: 395.1112.

Derivatization

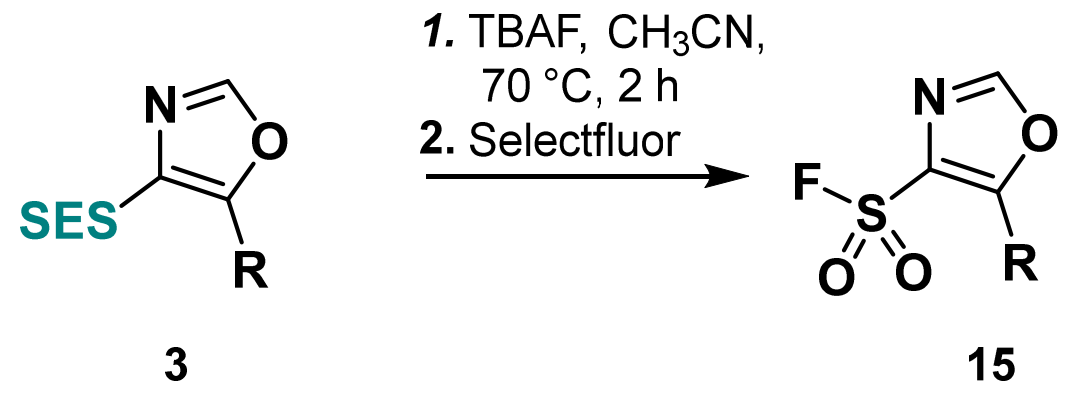

General Procedure B

A 1-dram vial was charged with sulfone (1 equiv) and TBAF (2 equiv, 1 M in THF). MeCN (0.28 M) was then added into the vial via syringe. The reaction was stirred at 70 °C for 2 h, until the disappearance of starting material (as shown by TLC). The reaction was allowed to cool to room temperature and then selectfluor (2 equiv) was added. The mixture was stirred at room temperature for 2 h. Upon completion, the reaction mixture was diluted with water, and the product was extracted with ethyl acetate. The organic extracts were dried over anhydrous Na_2SO_4, filtered, and concentrated under reduced pressure. The crude product was purified by flash chromatography (hexanes:ethyl acetate gradient from 0-25% over 10 min) to obtain **15**.

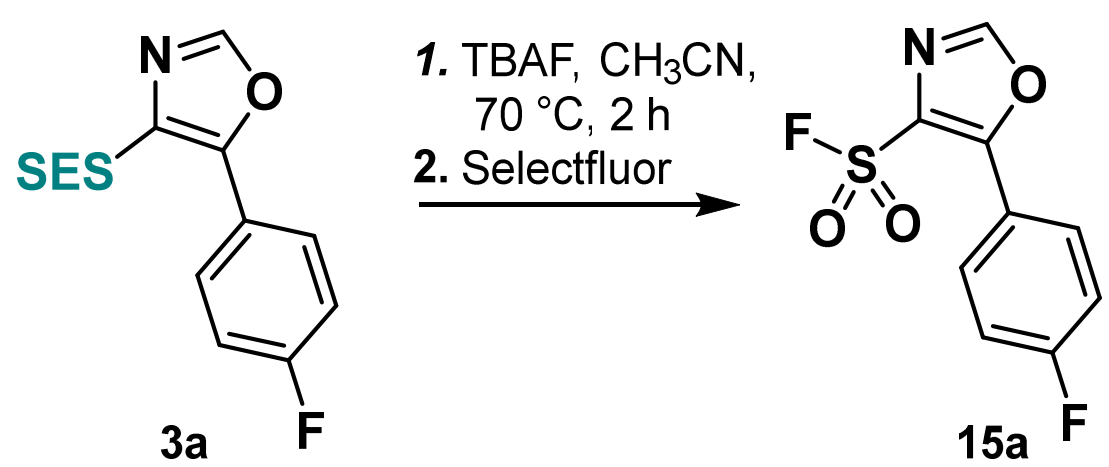

5-(4-fluorophenyl)oxazole-4-sulfonyl fluoride (15a)

According to General Procedure B, sulfone **3a** (25 mg, 0.085 mmol), TBAF (0.15 mL, 0.15 mmol, 2 equiv, 1 M in THF), MeCN (0.3 mL) and selectfluor (53 mg, 0.15 mmol, 2 equiv) yielded **15a** (11 mg, 0.044 mmol, 58% yield) as an amorphous solid.

^{1}H NMR (400 MHz, $CDCl_3$) δ 8.04 (s, 1H), 7.89 (dd, *J* = 8.8, 5.0 Hz, 2H), 7.23 (t, *J* = 8.8 Hz, 2H). **^{13}C{^{1}H} NMR** (126 MHz, C_6D_6) δ 164.4 (d, *J* = 253.8 Hz), 154.5 (d, *J* = 5.0 Hz), 149.1, 131.0 (d, *J* = 9.1 Hz), 128.0, 120.1 (d, *J* = 3.3 Hz), 115.7 (d, *J* = 24.0 Hz). **^{19}F{^{1}H, ^{13}C} NMR** (376 MHz, $CDCl_3$) δ 65.32, -105.66. **HRMS** (ESI-TOF) m/z: $[M+H]^+$ calc'd for $C_9H_6F_2NO_3S$: 246.0036; found: 246.0025.

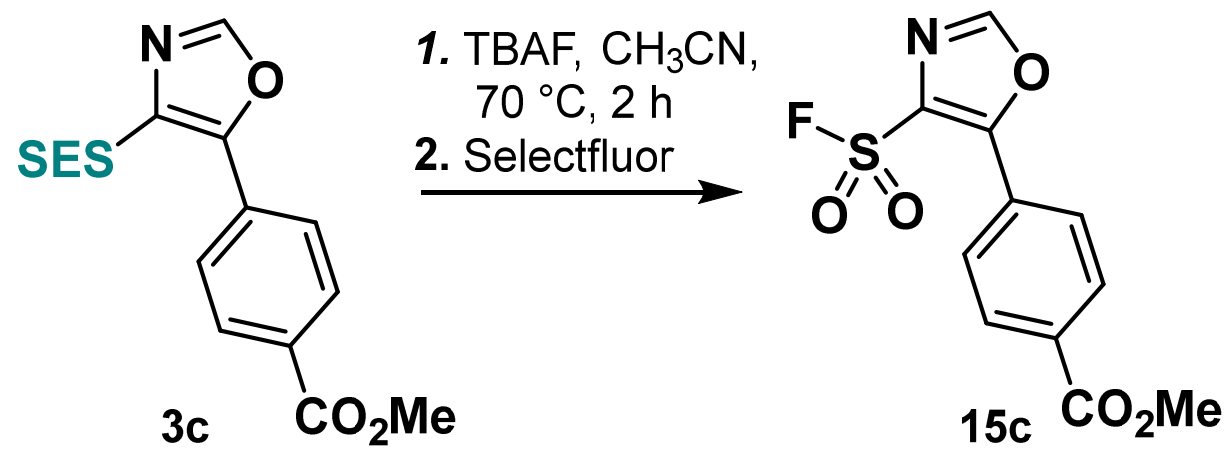

Methyl 4-(4-(fluorosulfonyl)oxazol-5-yl)benzoate (15c)

According to General Procedure B, sulfone **3c** (28 mg, 0.076 mmol), TBAF (0.15 mL, 0.15 mmol, 2 equiv, 1 M in THF), MeCN (0.3 mL) and selectfluor (54 mg, 0.15 mmol, 2 equiv) yielded **15c** (14 mg, 0.049 mmol, 65% yield) as a colorless oil.

^{1}H NMR (400 MHz, $CDCl_3$) δ 8.20 (dd, J = 8.3, 1.5 Hz, 2H), 8.09 (d, J = 1.4 Hz, 1H), 7.96 (dd, J = 8.5, 1.5 Hz, 2H), 3.97 (s, 3H). **^{13}C{^{1}H} NMR** (101 MHz, DMSO) δ 166.0, 150.3, 133.3, 130.4, 130.2, 129.0, 128.0, 124.3, 52.7. **^{19}F {^{1}H, ^{13}C} NMR** (376 MHz, $CDCl_3$) δ 65.41. **HRMS** (ESI-TOF) m/z: $[M+H]^+$ calc'd for $C_{11}H_9FNO_5S$: 286.0185; found: 286.0185.

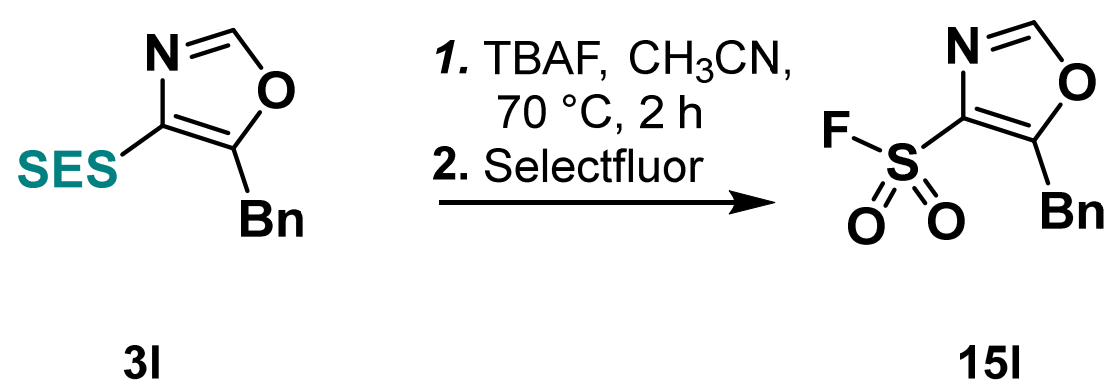

5-benzyloxazole-4-sulfonyl fluoride(15l)

According to General Procedure B, sulfone **3l** (42 mg, 0.130 mmol), TBAF (0.26 mL, 0.26 mmol, 2 equiv, 1 M in THF), MeCN (0.5 mL) and selectfluor (92 mg, 0.26 mmol, 2 equiv) yielded **15l** (16 mg, 0.066 mmol, 51% yield) as a yellow oil.

^{1}H NMR (400 MHz, $CDCl_3$) δ 7.89 (s, 1H), 7.39 – 7.27 (m, 5H), 4.37 (s, 2H). **^{13}C{^{1}H} NMR**(126 MHz, $CDCl_3$) δ 159.0, 150.4, 133.8, 129.1, 128.8, 127.9, 31.5. **^{19}F {^{1}H, ^{13}C} NMR** (376 MHz, $CDCl_3$) δ 65.26. **HRMS** (ESI-TOF) m/z: $[M+H]^+$ calc'd for $C_{10}H_9FNO_3S$: 242.0287; found: 242.0284.

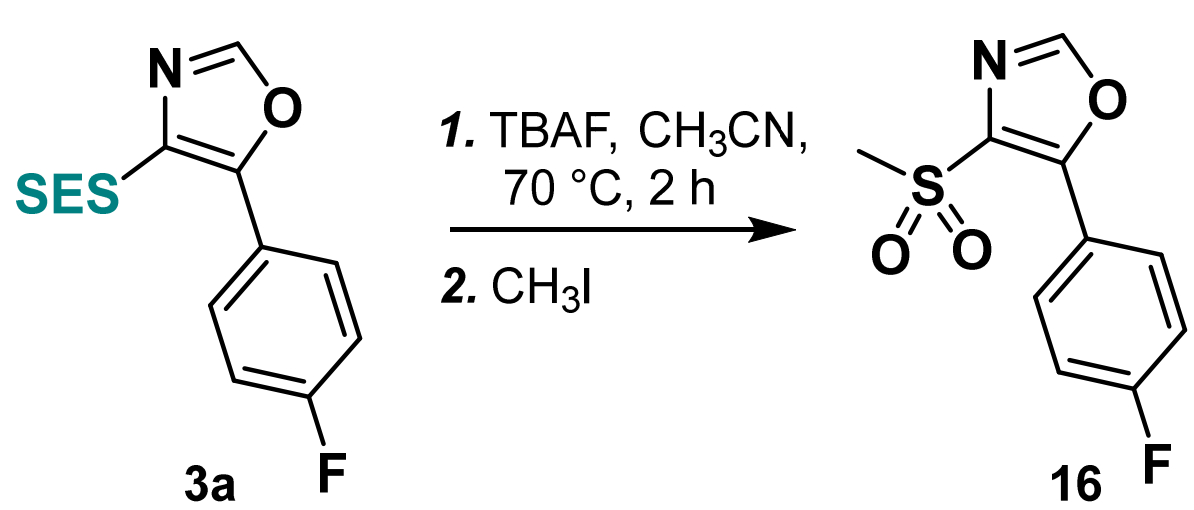

5-(4-fluorophenyl)-4-(methylsulfonyl)oxazole (16)

A 1-dram vial was charged with sulfone (36 mg, 0.11 mmol) and TBAF (0.22 mL, 0.22 mmol, 2 equiv, 1 M in THF). MeCN (0.4 mL) was then added into the vial via syringe. The reaction was

stirred at 70 °C for 2 h, until the disappearance of starting material (as shown by TLC). The reaction was allowed to cool to room temperature and then iodomethane (27.0 µL, 0.43 mmol, 4 equiv) was added. The mixture was stirred at room temperature for 2 h. Upon completion, the reaction mixture was diluted with water, and the product was extracted with ethyl acetate. The organic extracts were dried over anhydrous Na_2SO_4, filtered, and concentrated under reduced pressure. The crude product was purified by column chromatography (hexanes:ethyl acetate gradient from 0-30% over 15 min) to obtain **16** (13 mg, 0.052 mmol, 58 % yield) as an amorphous solid.

^{1}H NMR (400 MHz, $CDCl_3$) δ 8.01 – 7.93 (m, 3H), 7.19 (t, *J* = 8.5 Hz, 2H), 3.29 (s, 3H). **^{13}C{^{1}H} NMR** (101 MHz, $CDCl_3$) δ 164.4 (d, *J* = 253.3 Hz), 151.8, 149.1, 134.7, 131.3, 121.6, 116.1, 42.7. **^{19}F{^{1}H, ^{13}C} NMR** (376 MHz, $CDCl_3$) δ -107.75. **HRMS** (ESI-TOF) m/z: $[M+H]^+$ calc'd for $C_{10}H_9FNO_3S$: 242.0287; found: 242.0275.

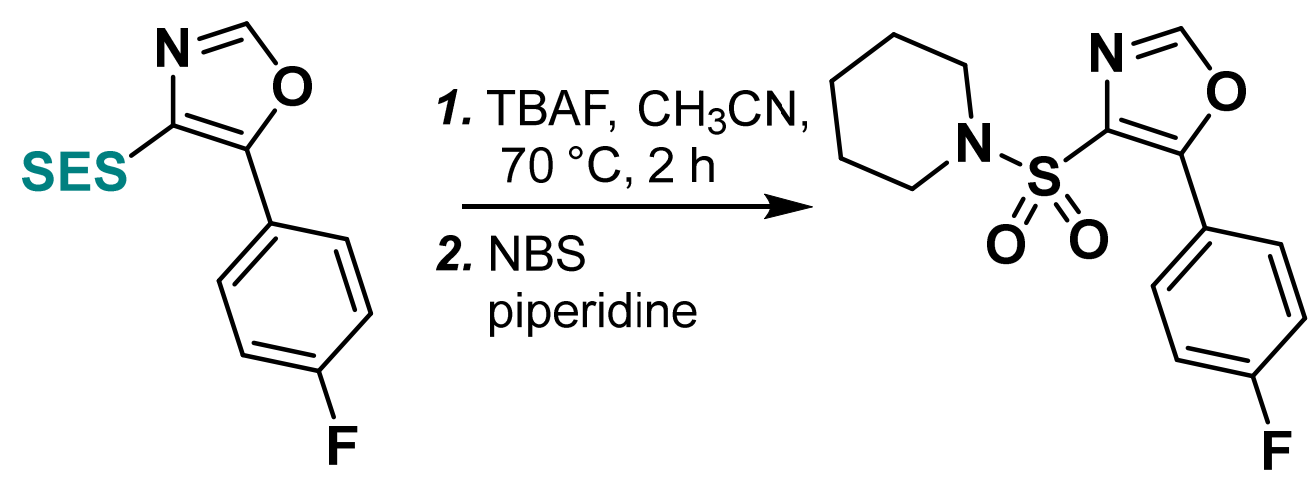

3a 17

5-(4-fluorophenyl)-4-(piperidin-1-ylsulfonyl)oxazole (17)

A 1-dram vial was charged with sulfone (20 mg, 0.061 mmol) and TBAF (0.12 mL, 0.12 mmol, 2 equiv, 1 M in THF). MeCN (0.24 mL) was then added into the vial via syringe. The reaction was stirred at 70 °C for 2 h, until the disappearance of starting material (as shown by TLC). The reaction was allowed to cool to room temperature and then *N*-bromosuccinimide (16 mg, 0.092 mmol, 1.5 equiv) and piperidine (60.3 µL, 0.61 mmol, 10 equiv) were added. The mixture was stirred at room temperature overnight. Upon completion, the reaction mixture was diluted with water, and the product was extracted with ethyl acetate. The organic extracts were dried over anhydrous Na_2SO_4, filtered, and concentrated under reduced pressure. The crude product was purified by flash chromatography (hexanes:ethyl acetate gradient from 0-20% over 15 min) to obtain **17** (12 mg, 0.040 mmol, 65 % yield) as yellow oil.

^{1}H NMR (400 MHz, $CDCl_3$) δ 8.01 – 7.93 (m, 2H), 7.90 (s, 1H), 7.16 (t, *J* = 8.7 Hz, 2H), 3.37 – 3.29 (m, 4H), 1.65 (p, *J* = 5.5 Hz, 4H), 1.54 (qd, *J* = 6.1, 3.5 Hz, 2H). **^{13}C{^{1}H} NMR** (126 MHz, $CDCl_3$) δ 164.1 (d, *J* = 252.3 Hz), 151.6, 149.0, 133.6, 131.0, 122.1, 116.1, 47.4, 25.5, 23.8. **^{19}F{^{1}H, ^{13}C} NMR** (376 MHz, $CDCl_3$) δ -108.71. **HRMS** (ESI-TOF) m/z: $[M+H]^+$ calc'd for $C_{14}H_{16}FN_2O_3S$: 311.0866; found: 311.0858.

Stability Testing

100 mM stock solutions of sulfonyl fluoride **15a**, commercially available 2-phenylbenzene-1-sulfonyl fluoride **18**, and internal standard (4-F-acetophenone) were prepared in DMSO-d6. 12.5 µL each of sulfonyl fluoride and internal standard were combined in a microcentrifuge tube followed by addition of 125 µL of DMSO-d6. To this centrifuge tube was added 350 µL of 1X

PBS (pH 7.4). Final concentrations were 2.5 mM sulfonyl fluoride and 2.5 mM internal standard in 30% DMSO-d6. After vortexing, the solution was quickly transferred to an NMR tube for analysis. $^{19}F\{^{1}H, ^{13}C\}$ NMR spectra were taken every 30 minutes in triplicate and data was plotted in GraphPad Prism and fit using a one phase decay model to yield half-life.

References

1. Mamone, P.; Grünberg, M. F.; Fromm, A.; Khan, B. A.; Gooßen, L. J., [Pd(μ-Br)(PtBu3)]2 as a Highly Active Isomerization Catalyst: Synthesis of Enol Esters from Allylic Esters. *Organic Letters* **2012,** *14* (14), 3716-3719.

Spectra

^{1}H ($CDCl_3$, 400 MHz) **S2**

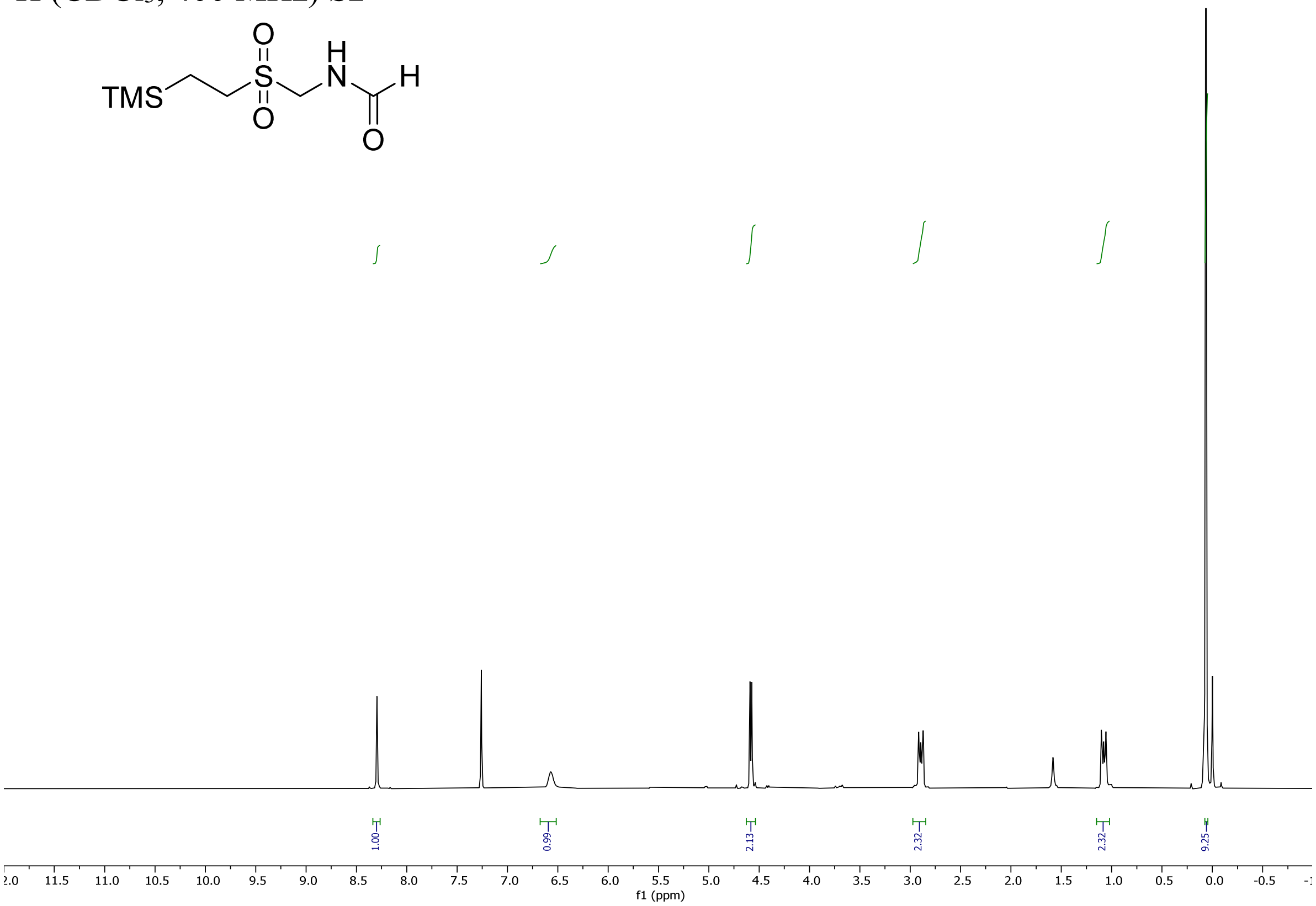

$^{13}C\{^{1}H\}$ ($CDCl_3$; 101 MHz) **S2**

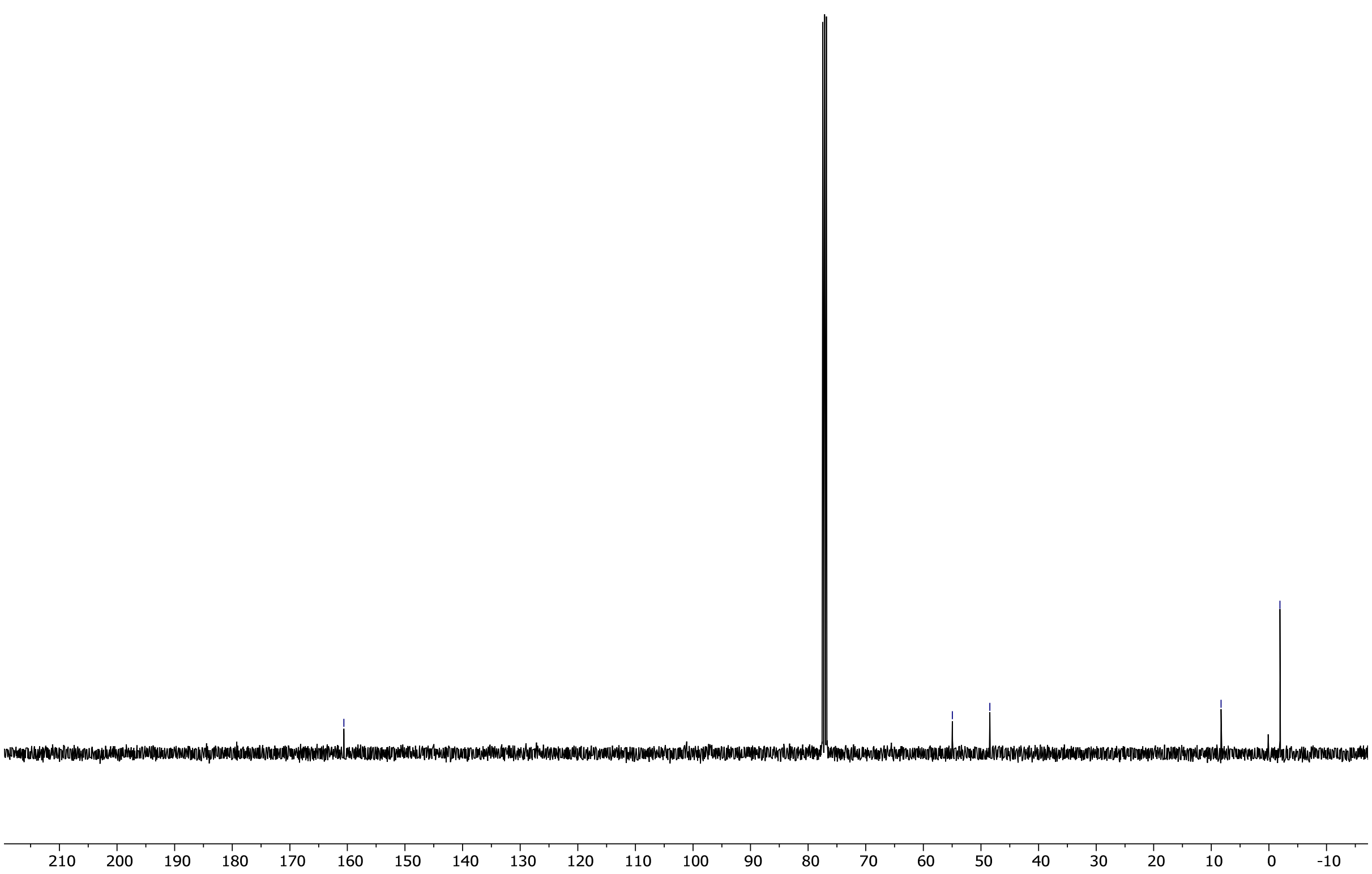

^{1}H ($CDCl_3$, 400 MHz) **SESMic (1)**

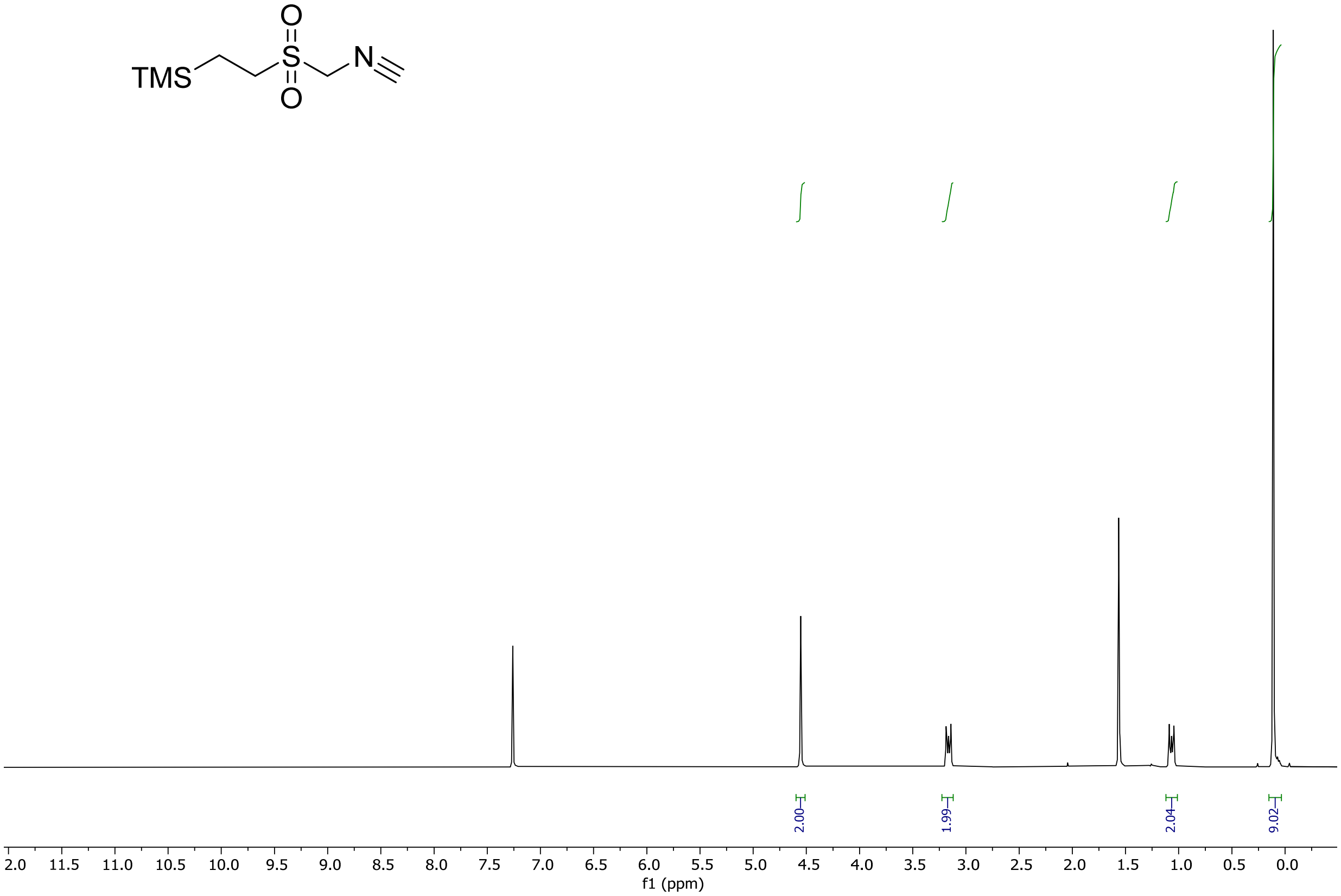

$^{13}C\{^{1}H\}$ (C_6D_6; 101 MHz)

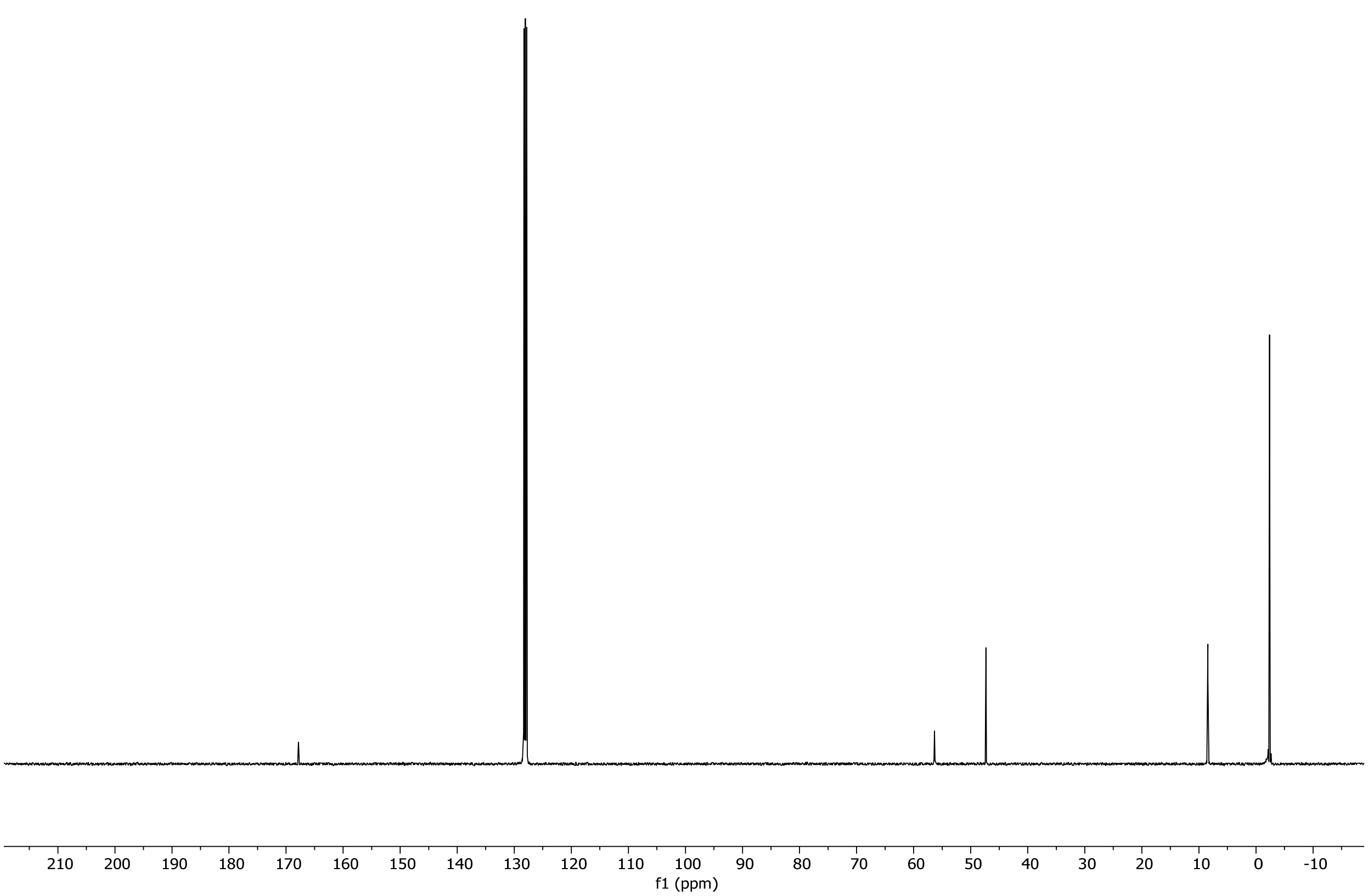

^{1}H ($CDCl_3$, 400 MHz) **3a**

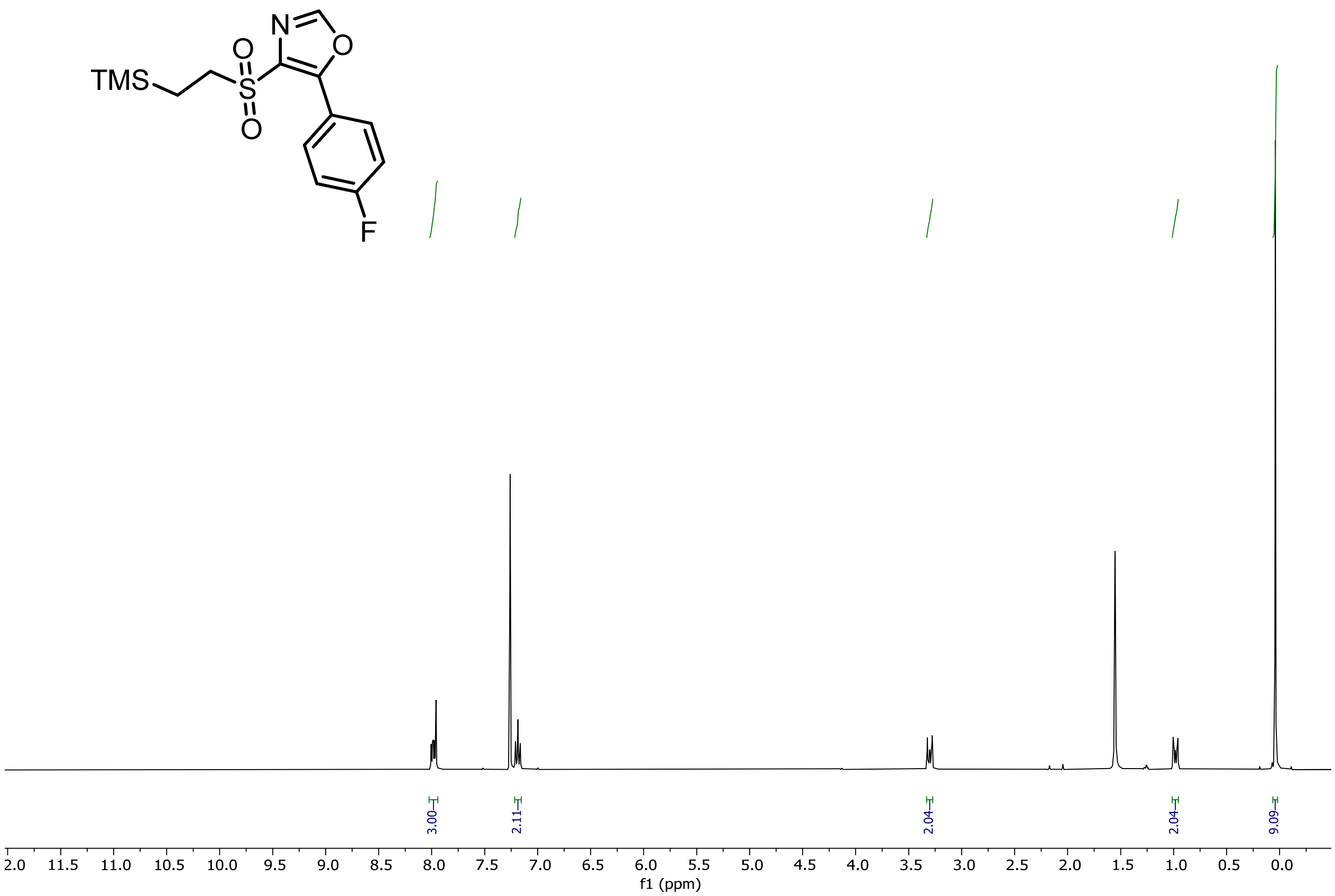

^{13}**C{^{1}H}** (CDCl3; 101 MHz) **3a**

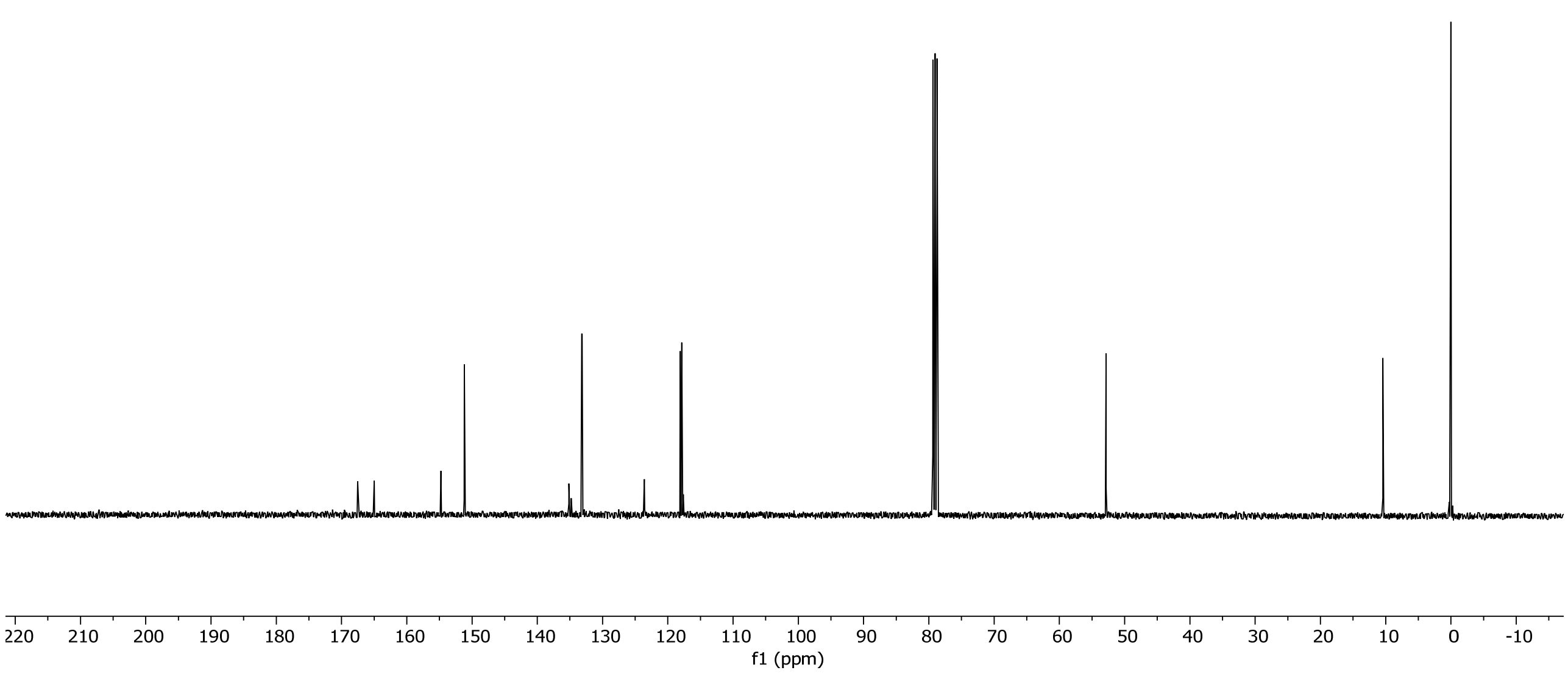

$^{19}F\{^{1}H, ^{13}C\}$ ($CDCl_3$, 376 MHz) **3a**

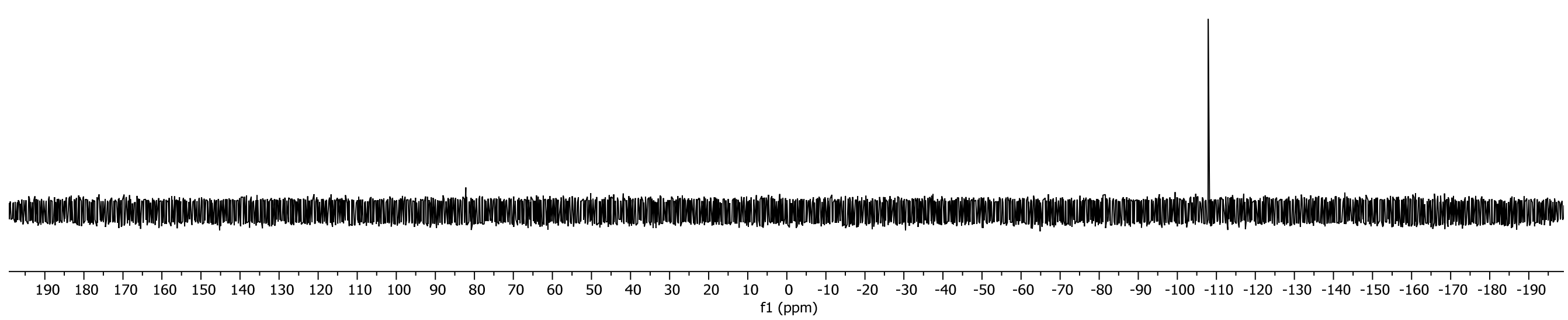

^{1}H (CDCl3, 400 MHz) **3b**

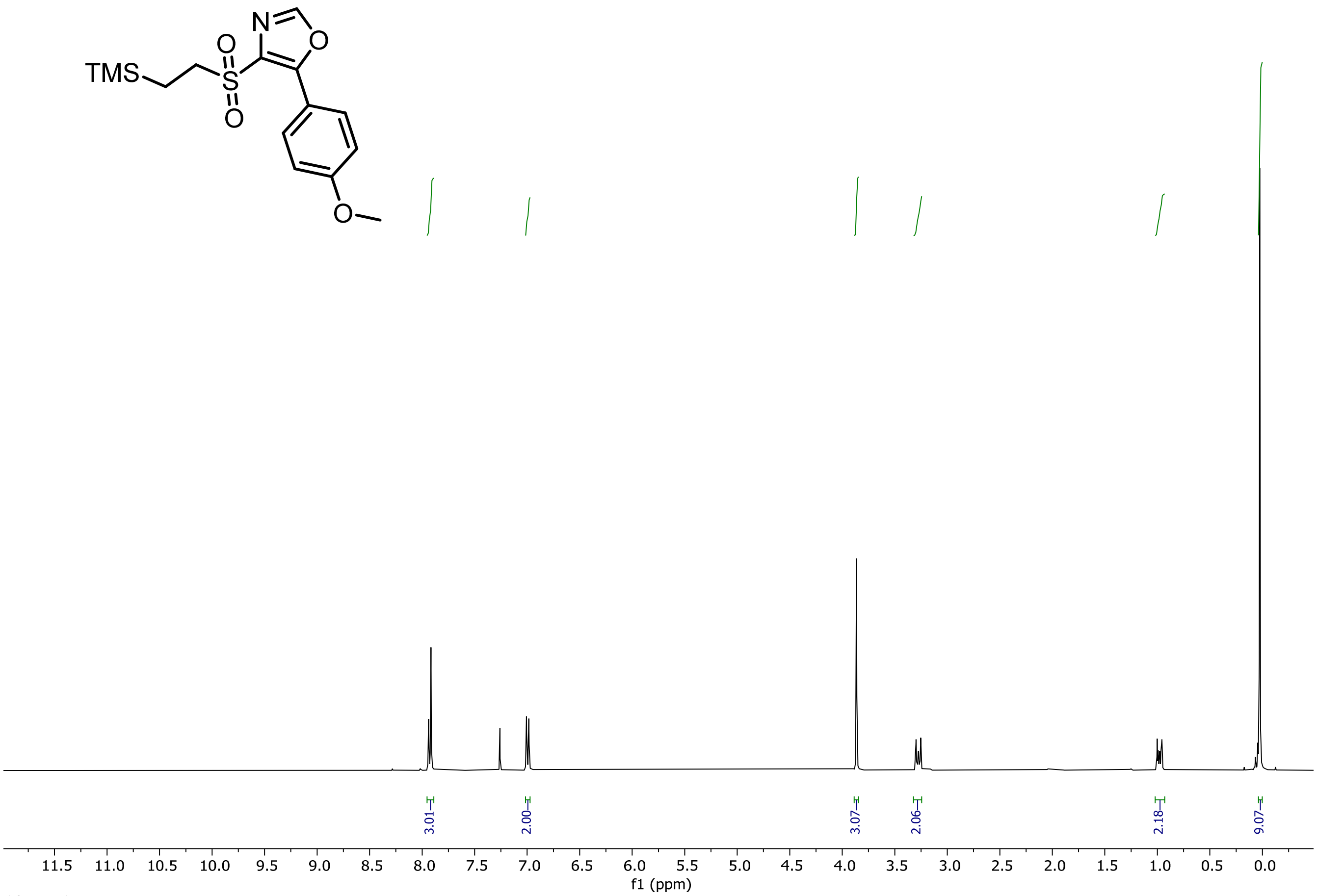

^{13}C{^{1}H} (CDCl3; 101 MHz) **3b**

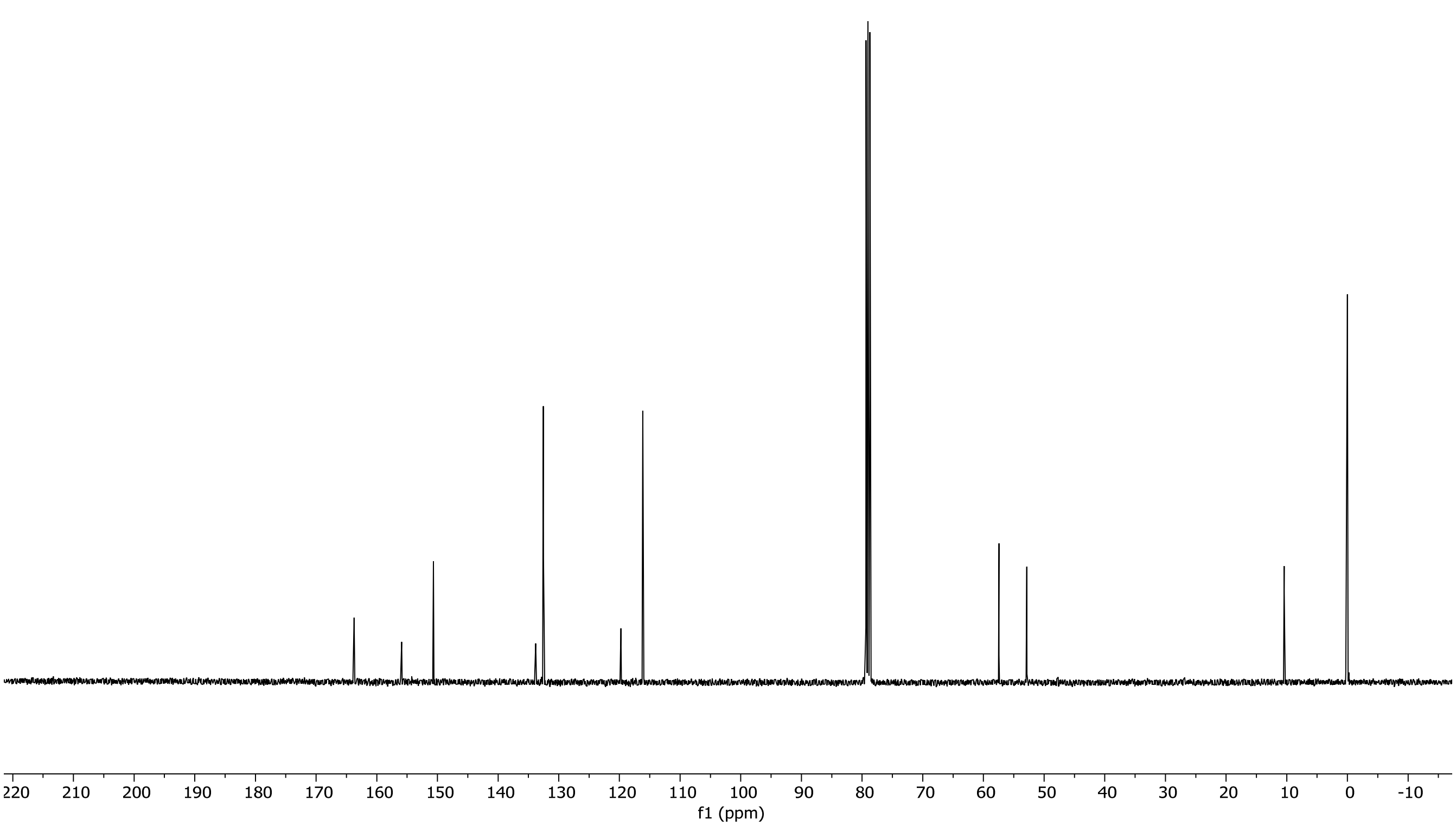

^{1}H ($CDCl_3$, 400 MHz) **3c**

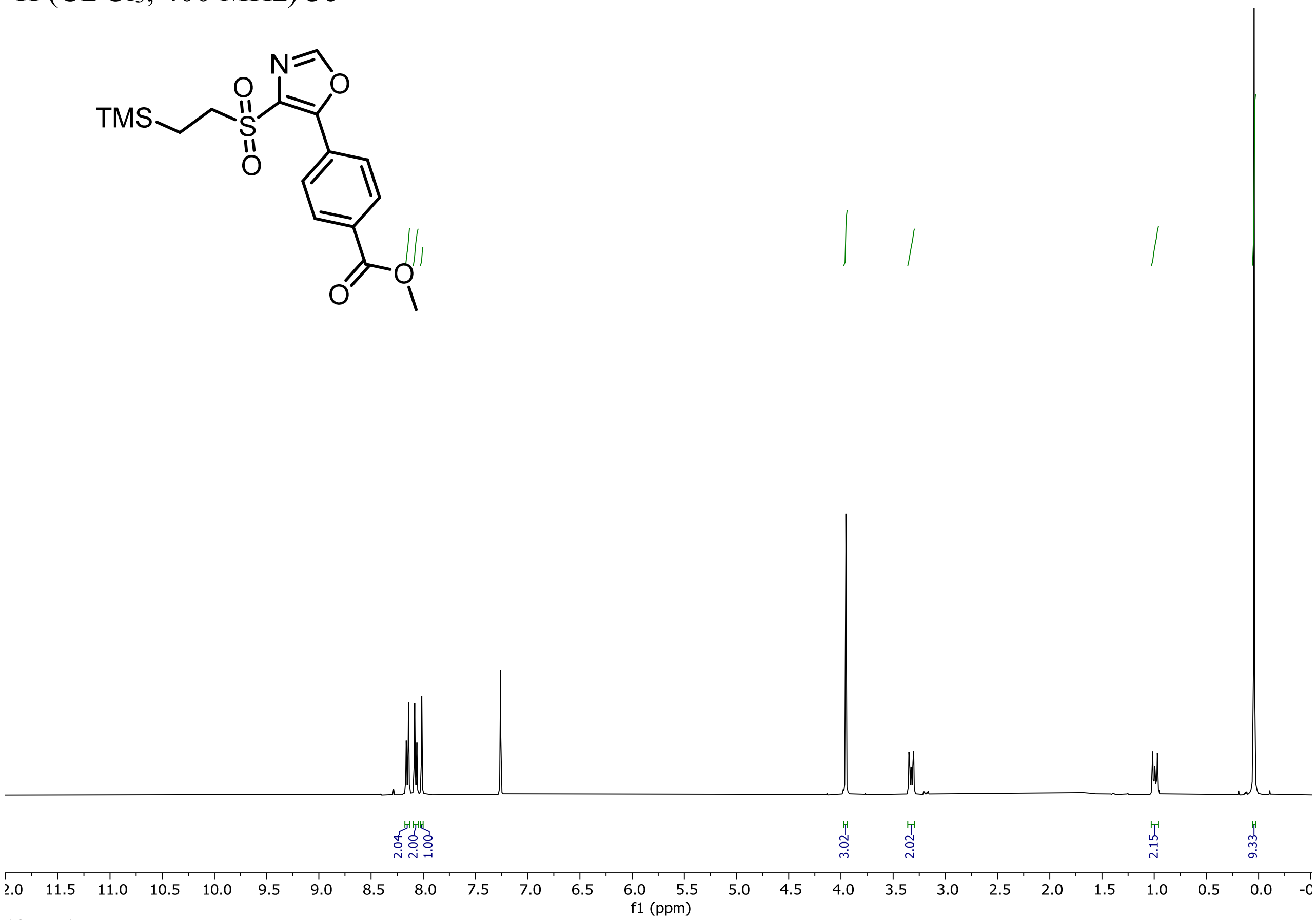

$^{13}C\{^{1}H\}$ (CDCl3; 101 MHz) **3c**

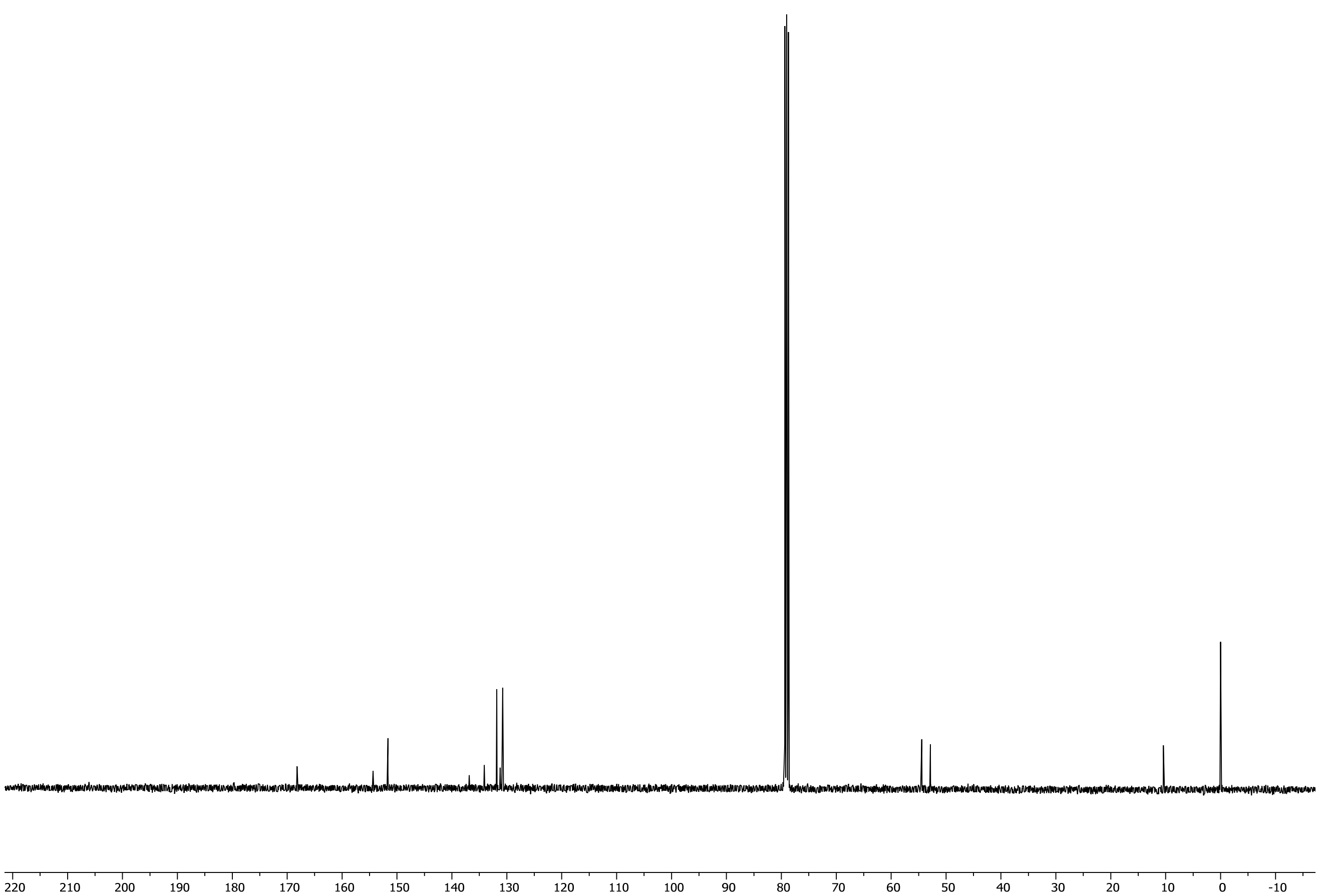

^{1}H (CDCl$_3$, 400 MHz) **3d**

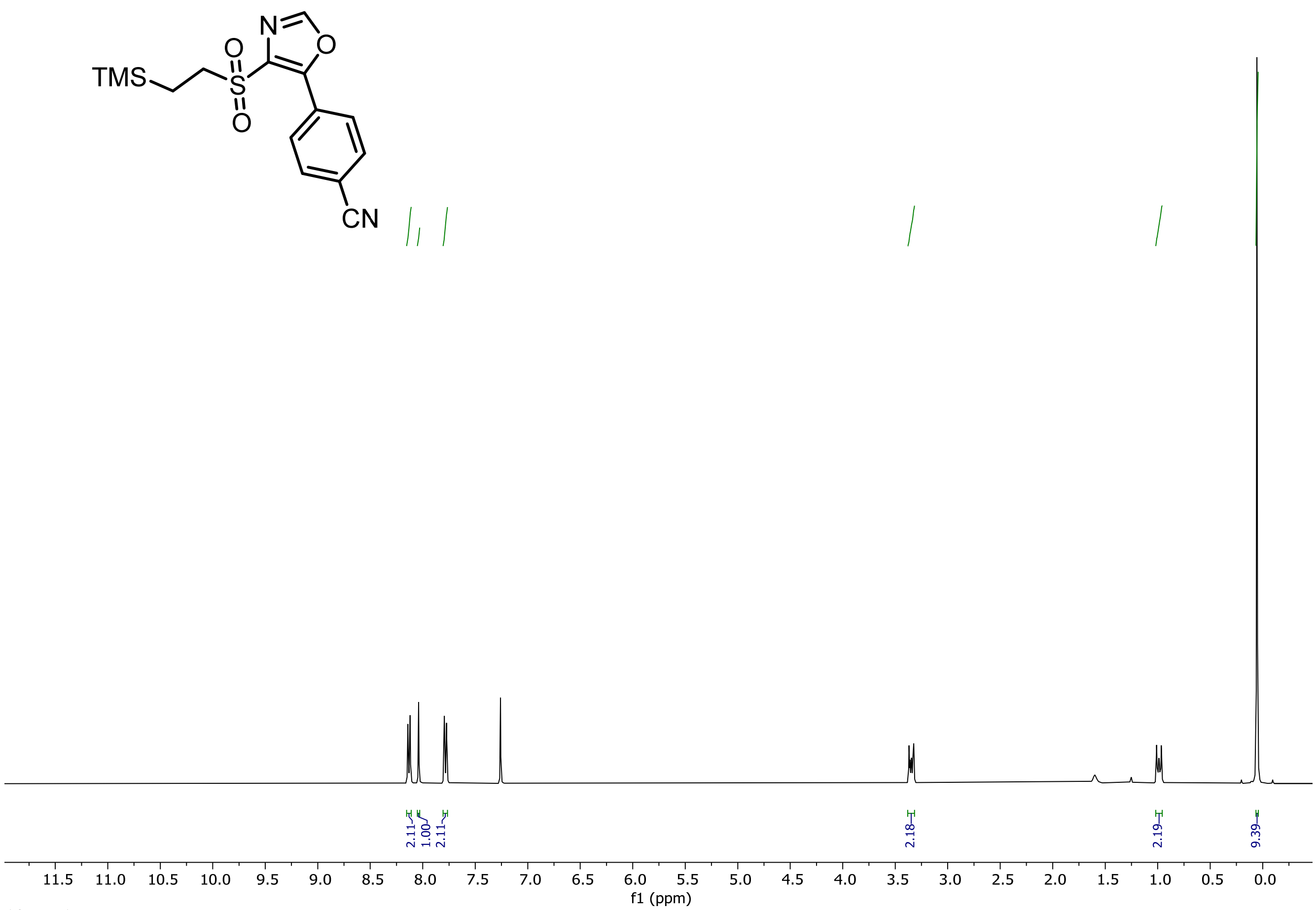

$^{13}C\{^{1}H\}$ (CDCl$_3$; 101 MHz) **3d**

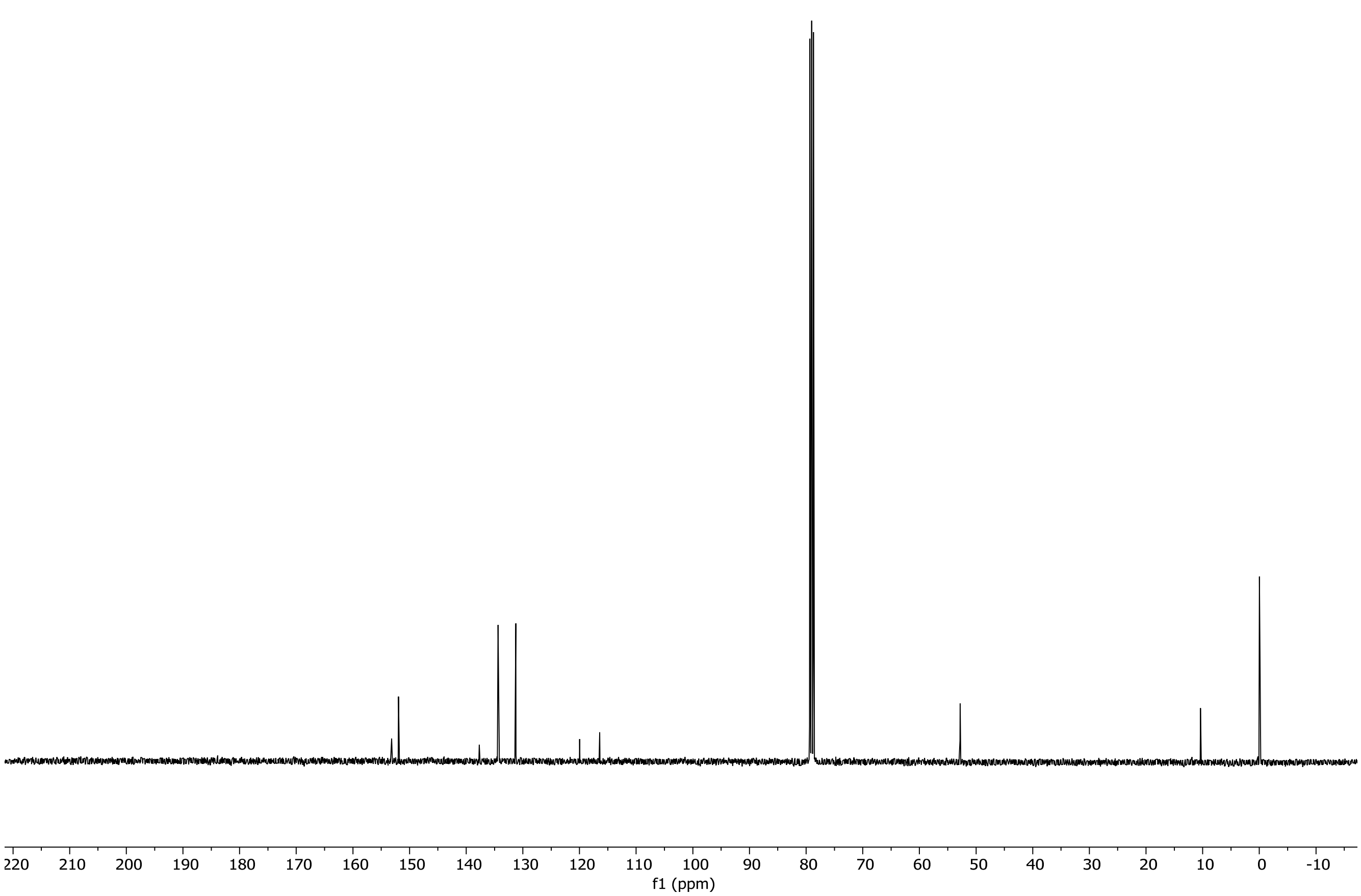

^{1}H ($CDCl_3$, 400 MHz) **3e**

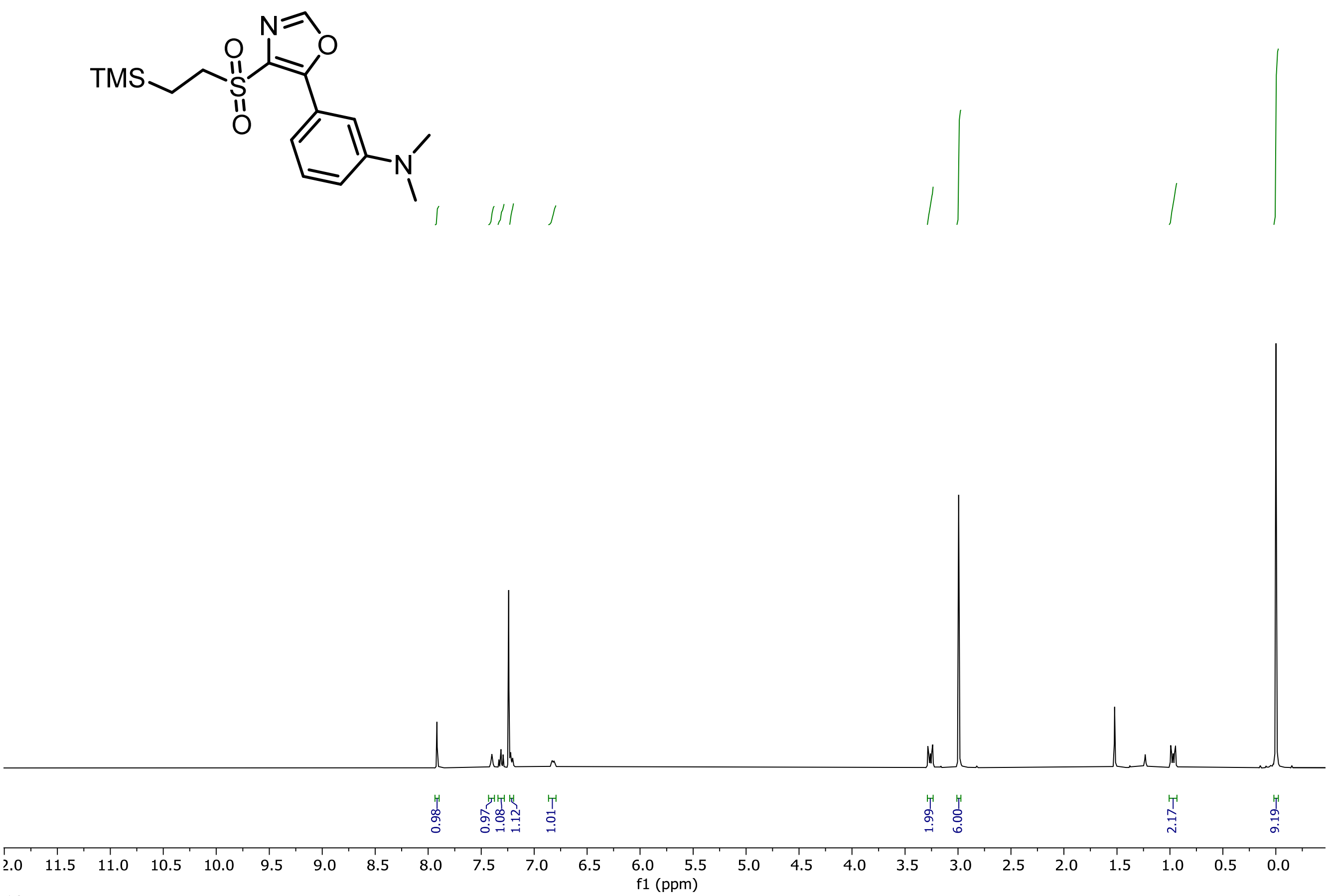

$^{13}C\{^{1}H\}$ ($CDCl_3$; 101 MHz) **3e**

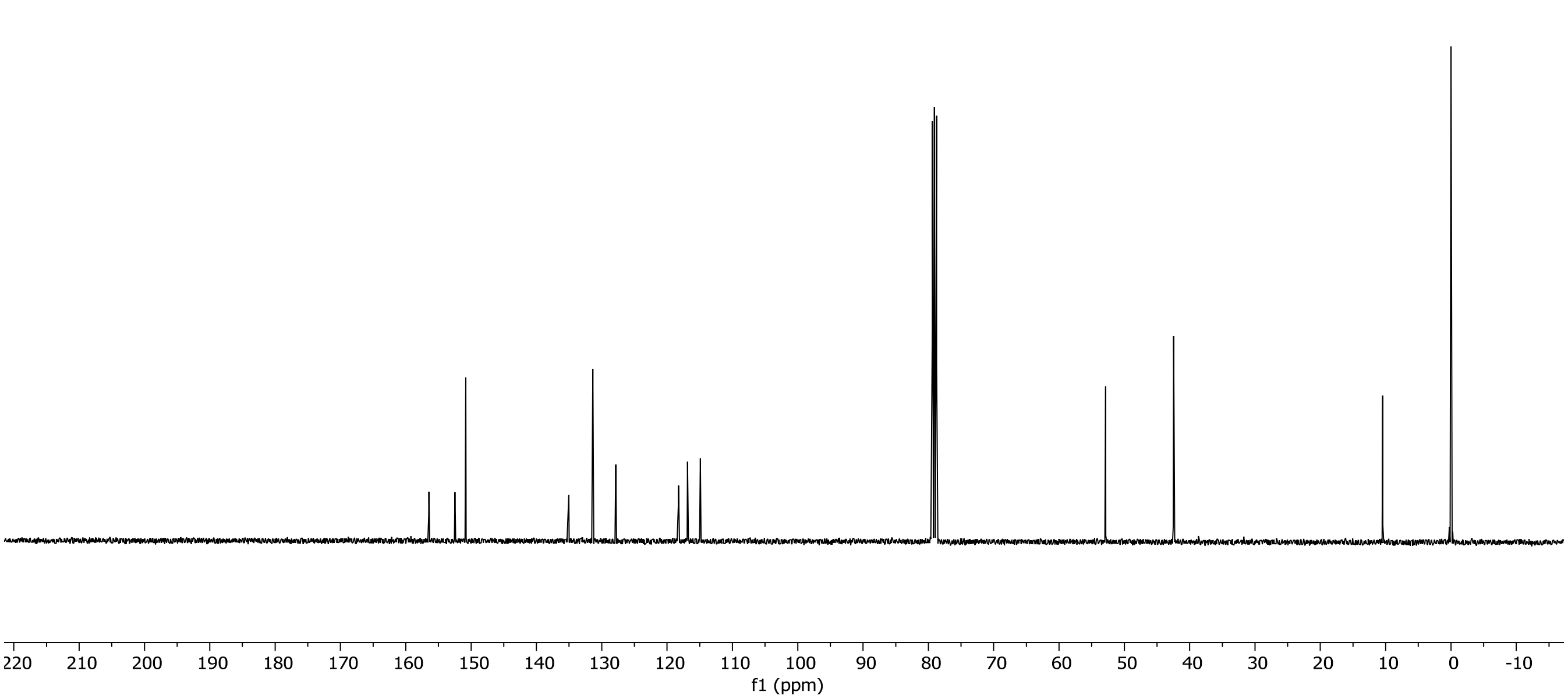

^{1}H ($CDCl_3$, 400 MHz) **3f**

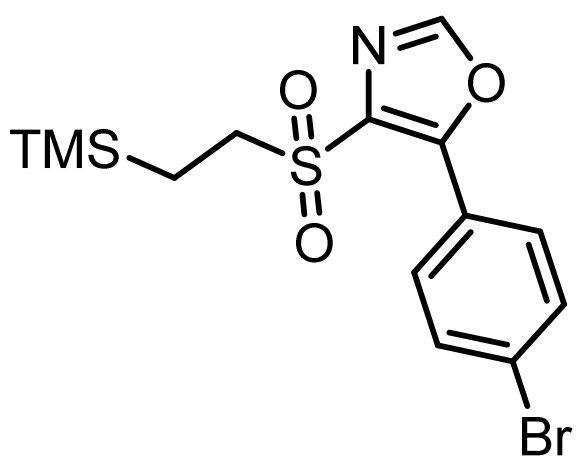

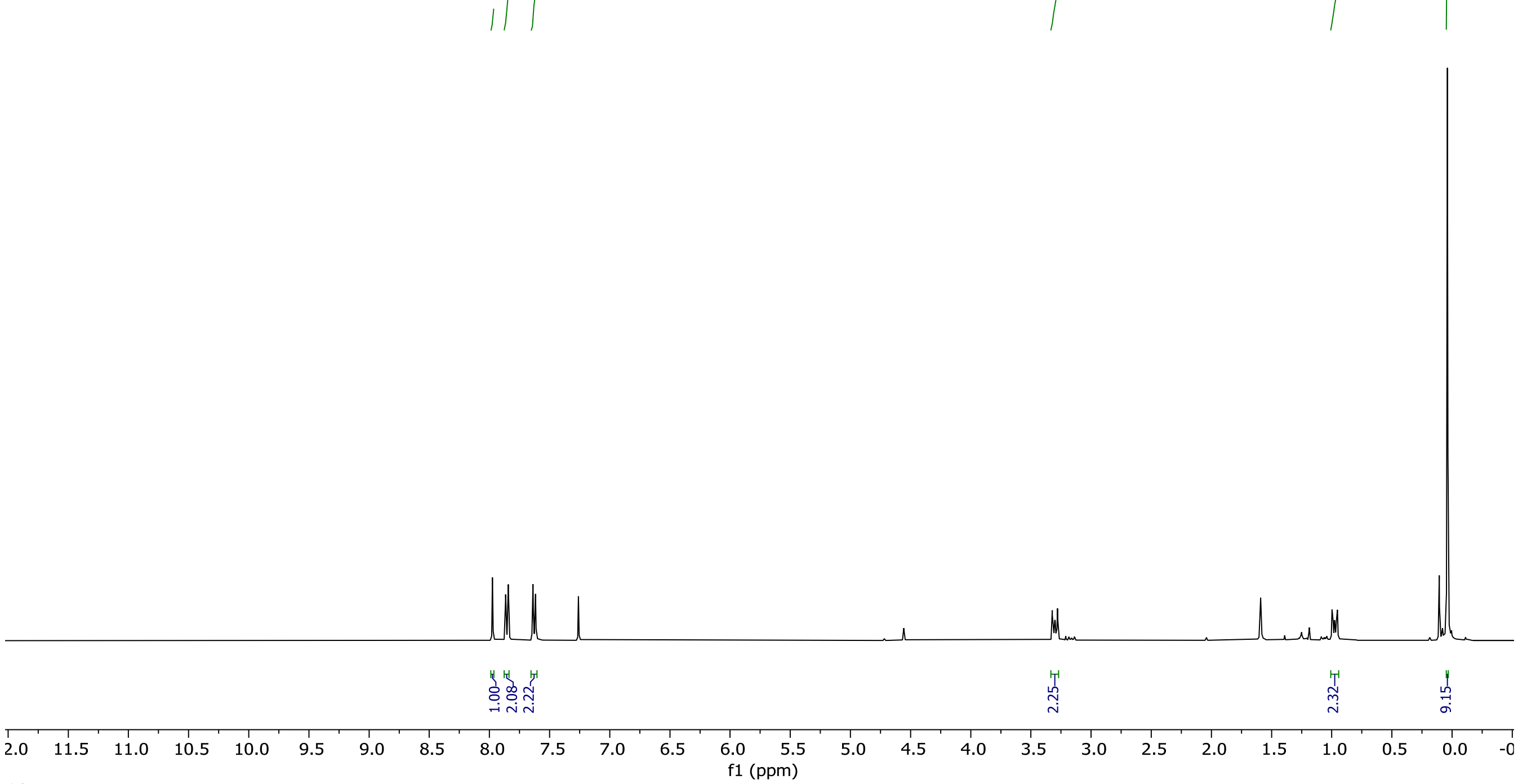

$^{13}C\{^{1}H\}$ ($CDCl_3$; 101 MHz) **3f**

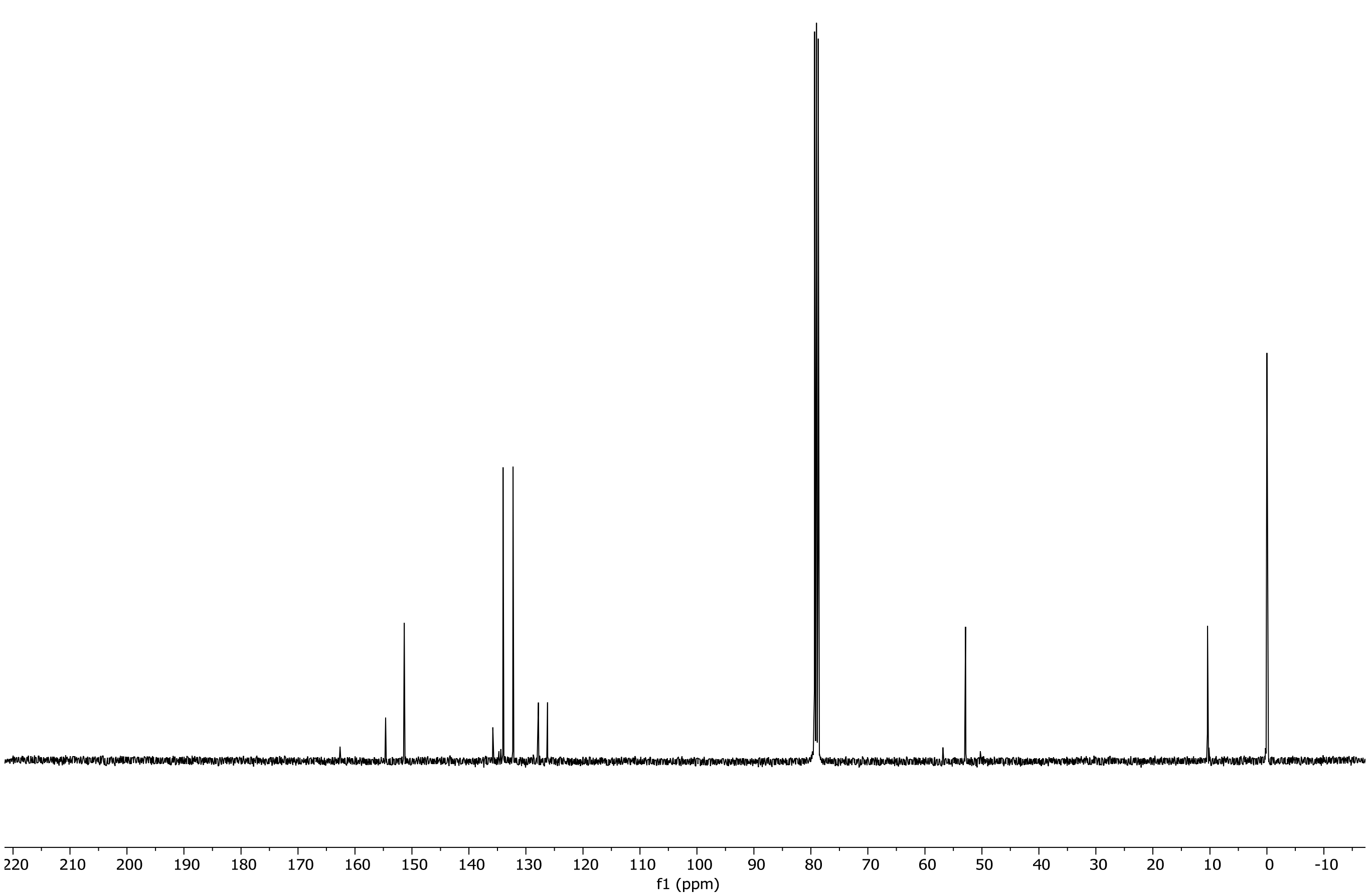

^{1}H ($CDCl_3$, 400 MHz) **3g**

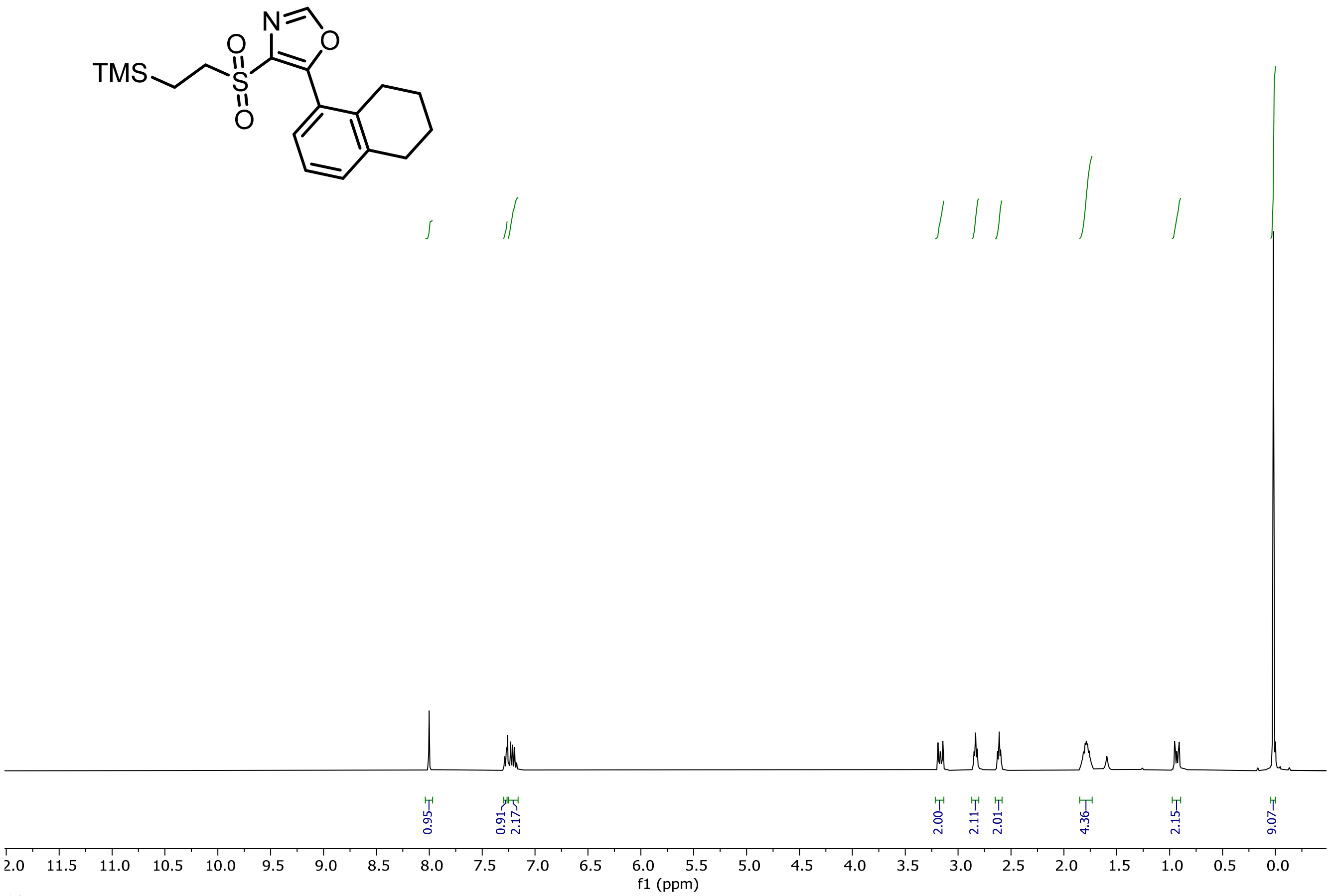

$^{13}C\{^{1}H\}$ ($CDCl_3$; 101 MHz) **3g**

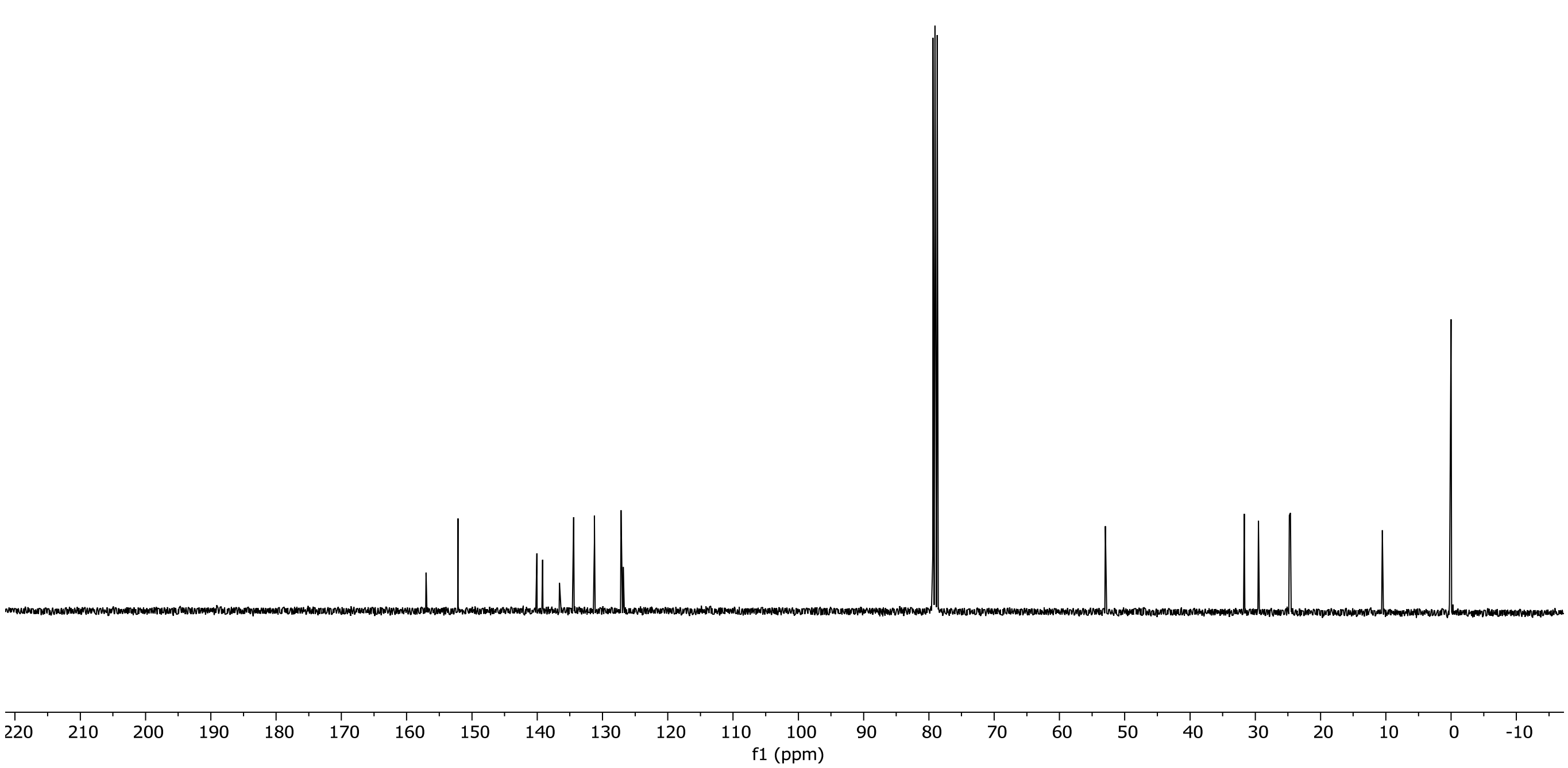

^{1}H ($CDCl_3$, 400 MHz) **3h**

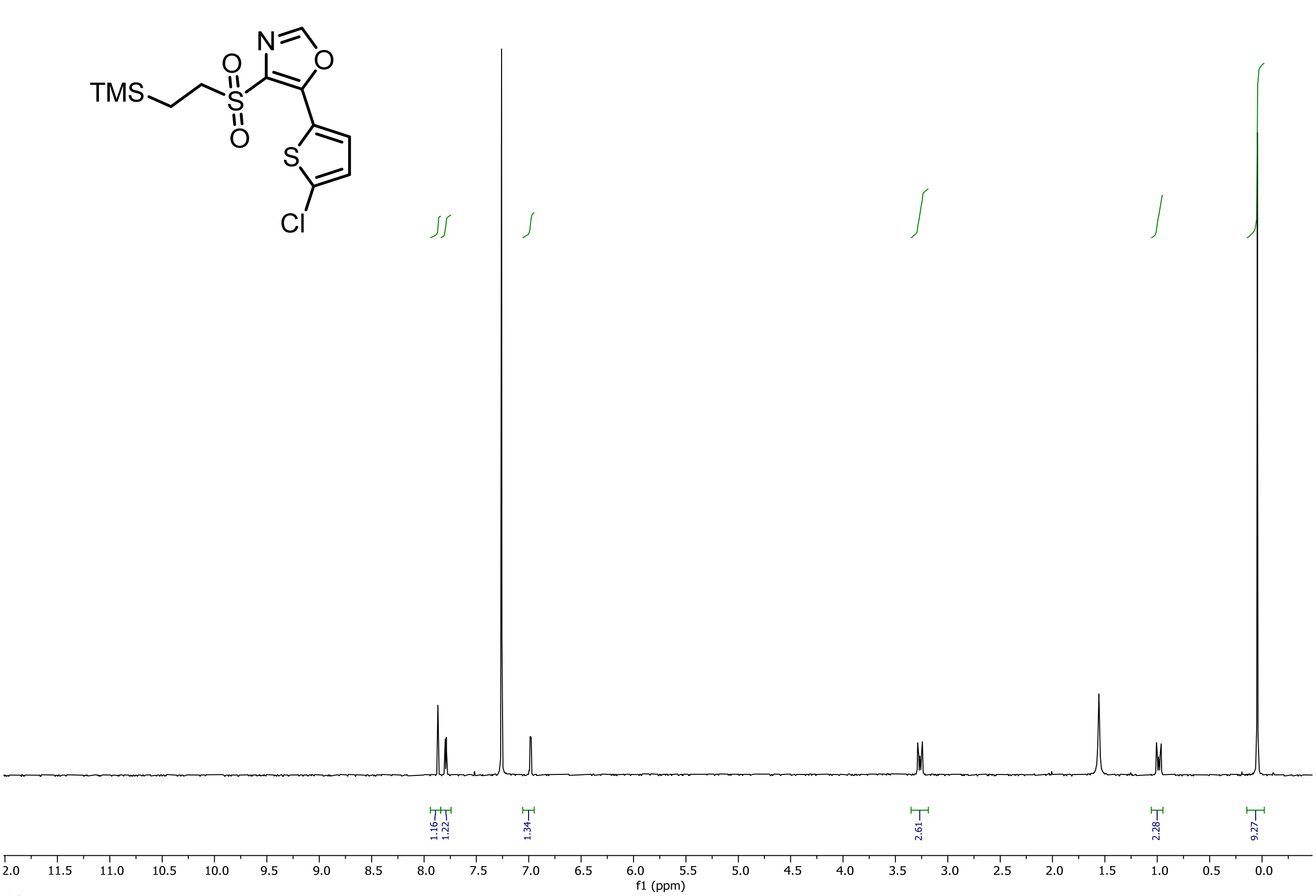

$^{13}C\{^{1}H\}$ ($CDCl_3$; 101 MHz) **3h**

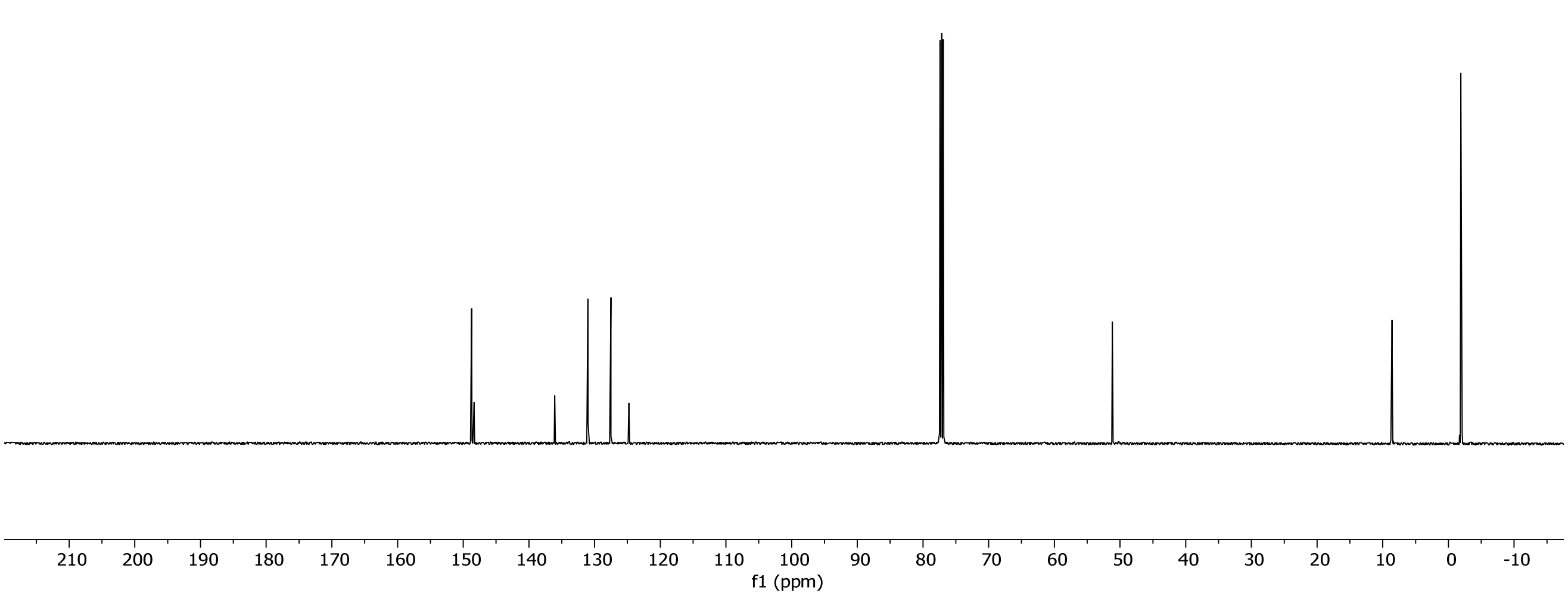

^{1}H ($CDCl_3$, 400 MHz) **3i**

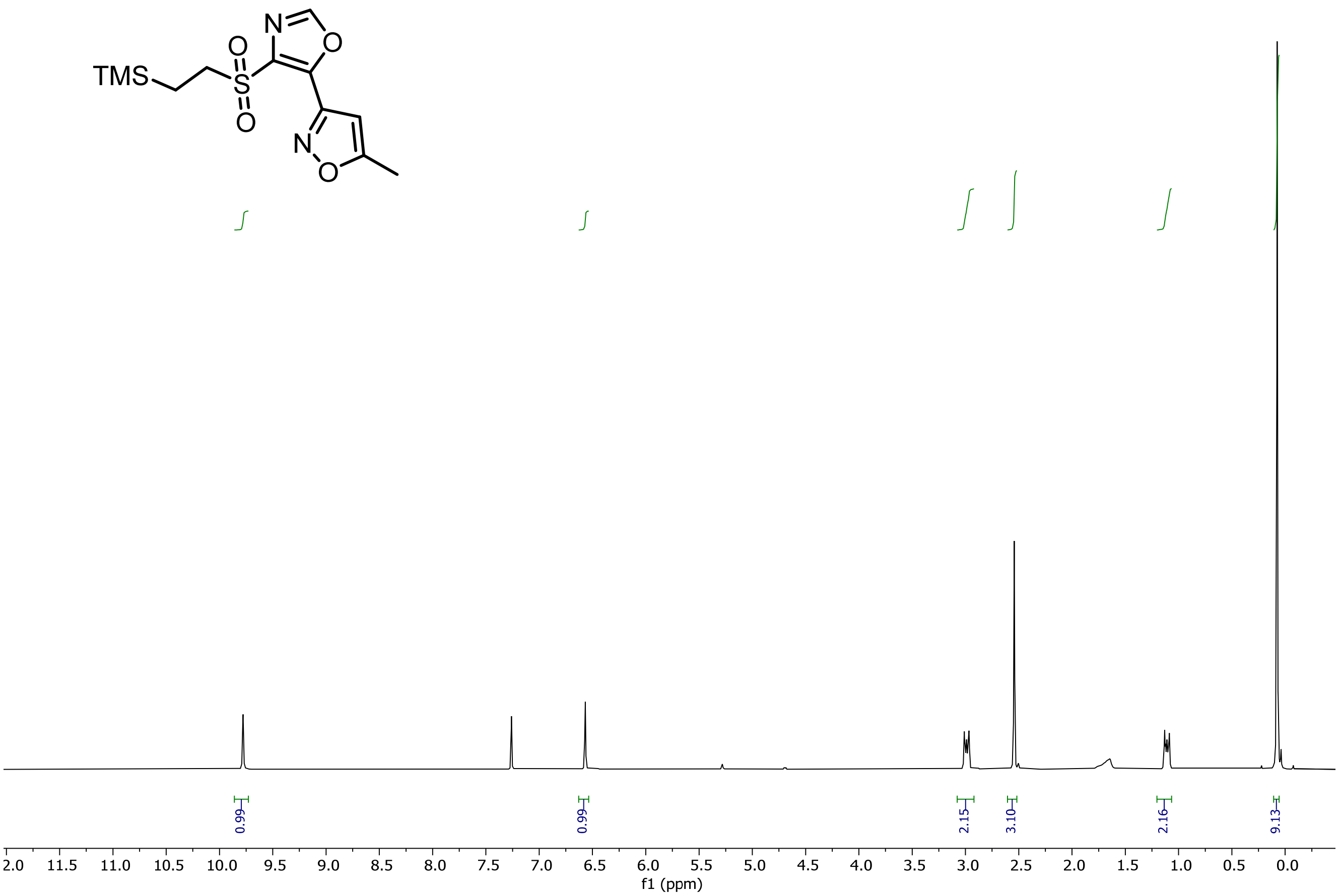

$^{13}C\{^{1}H\}$ ($CDCl_3$; 101 MHz) **3i**

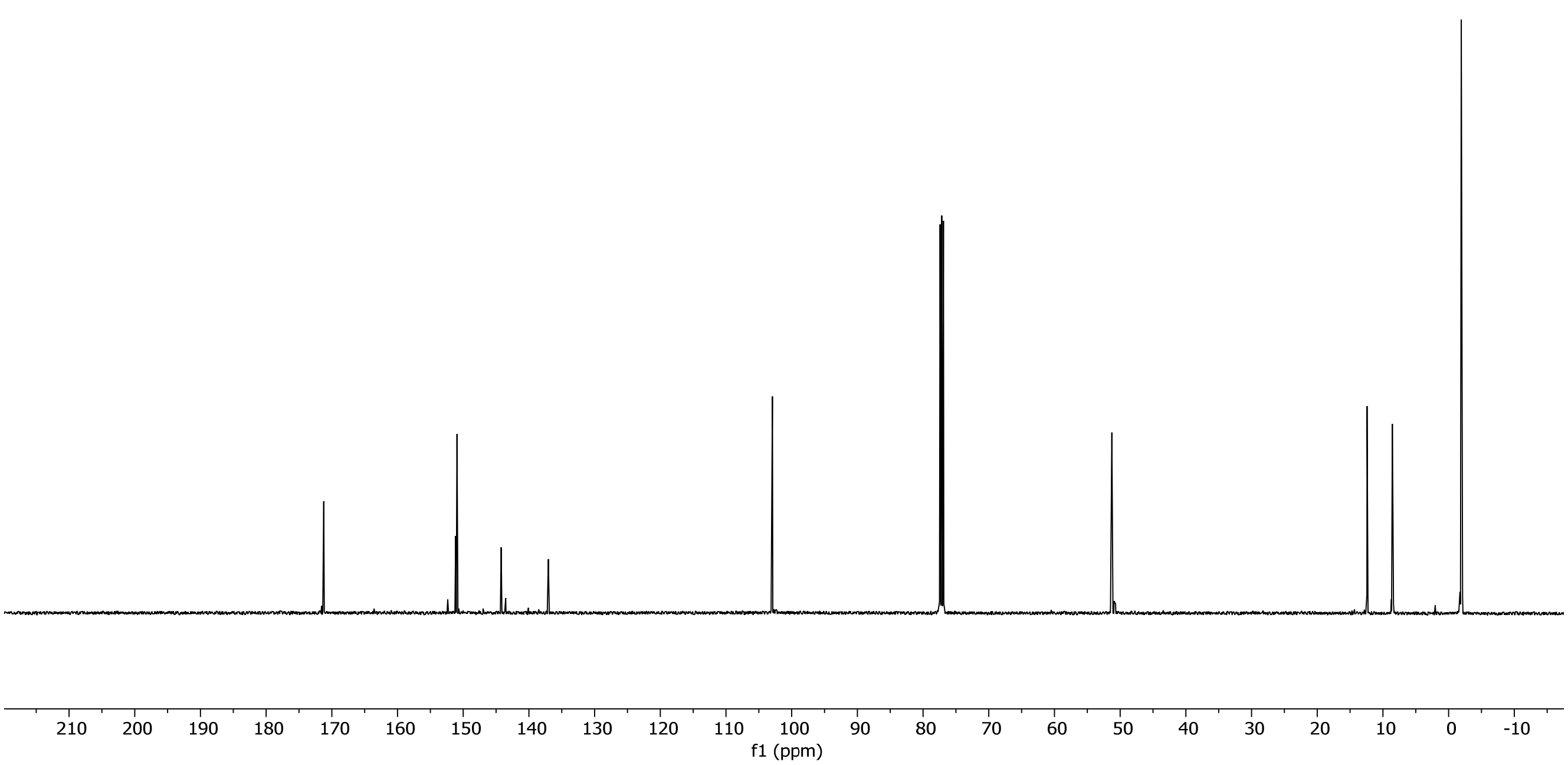

^{1}H (CDCl$_3$, 400 MHz) **3j**

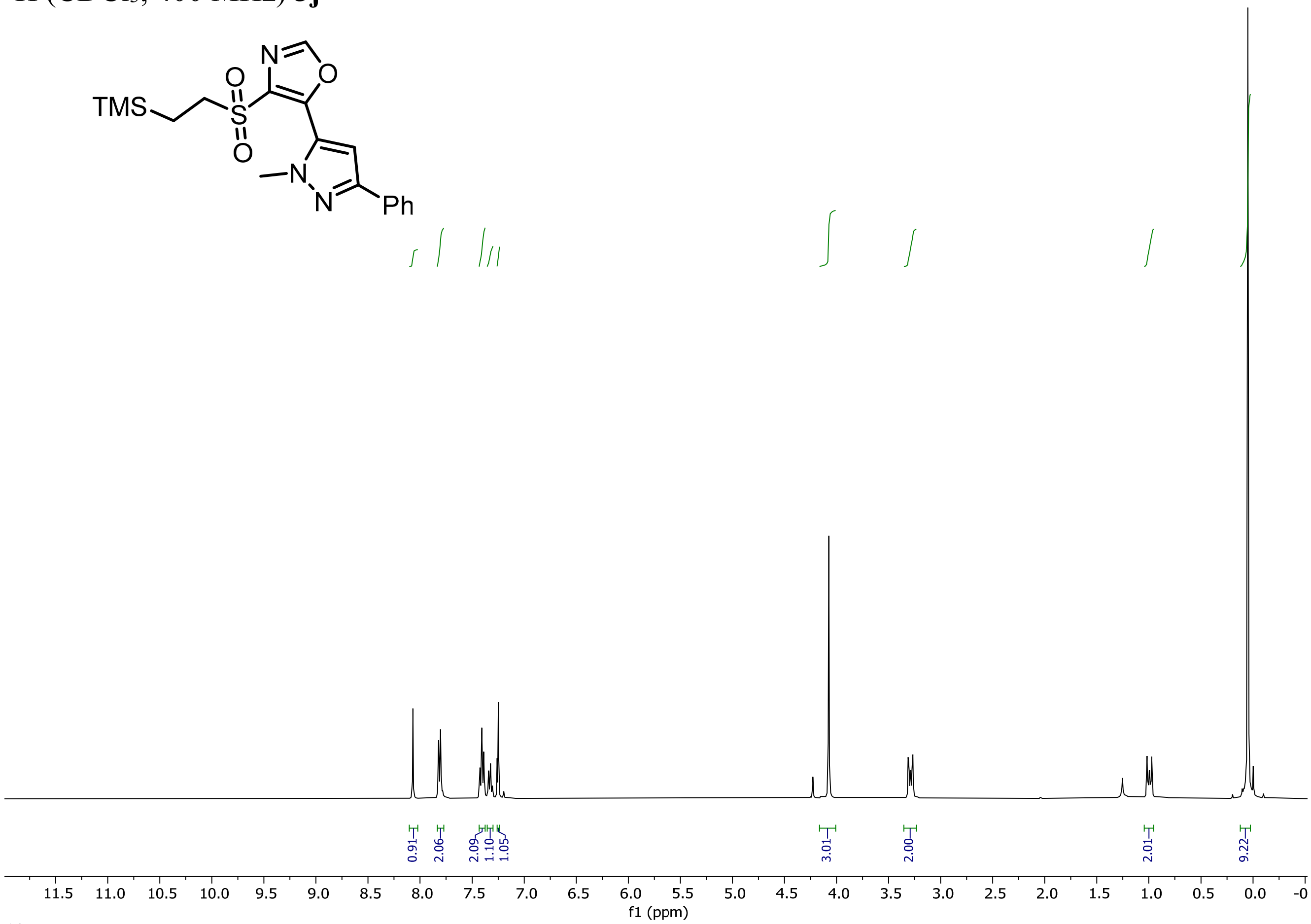

$^{13}C\{^{1}H\}$ (CDCl$_3$; 101 MHz) **3j**

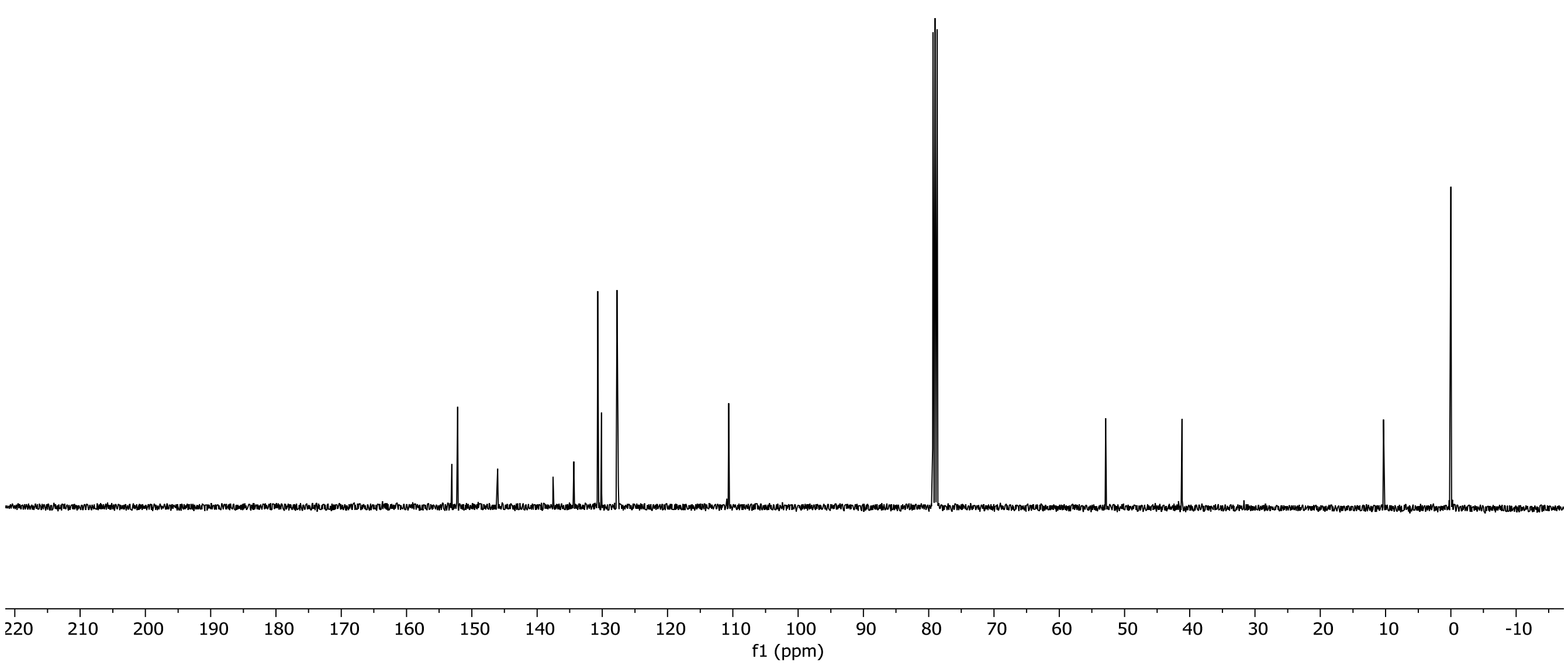

^{1}H ($CDCl_3$, 400 MHz) **3k**

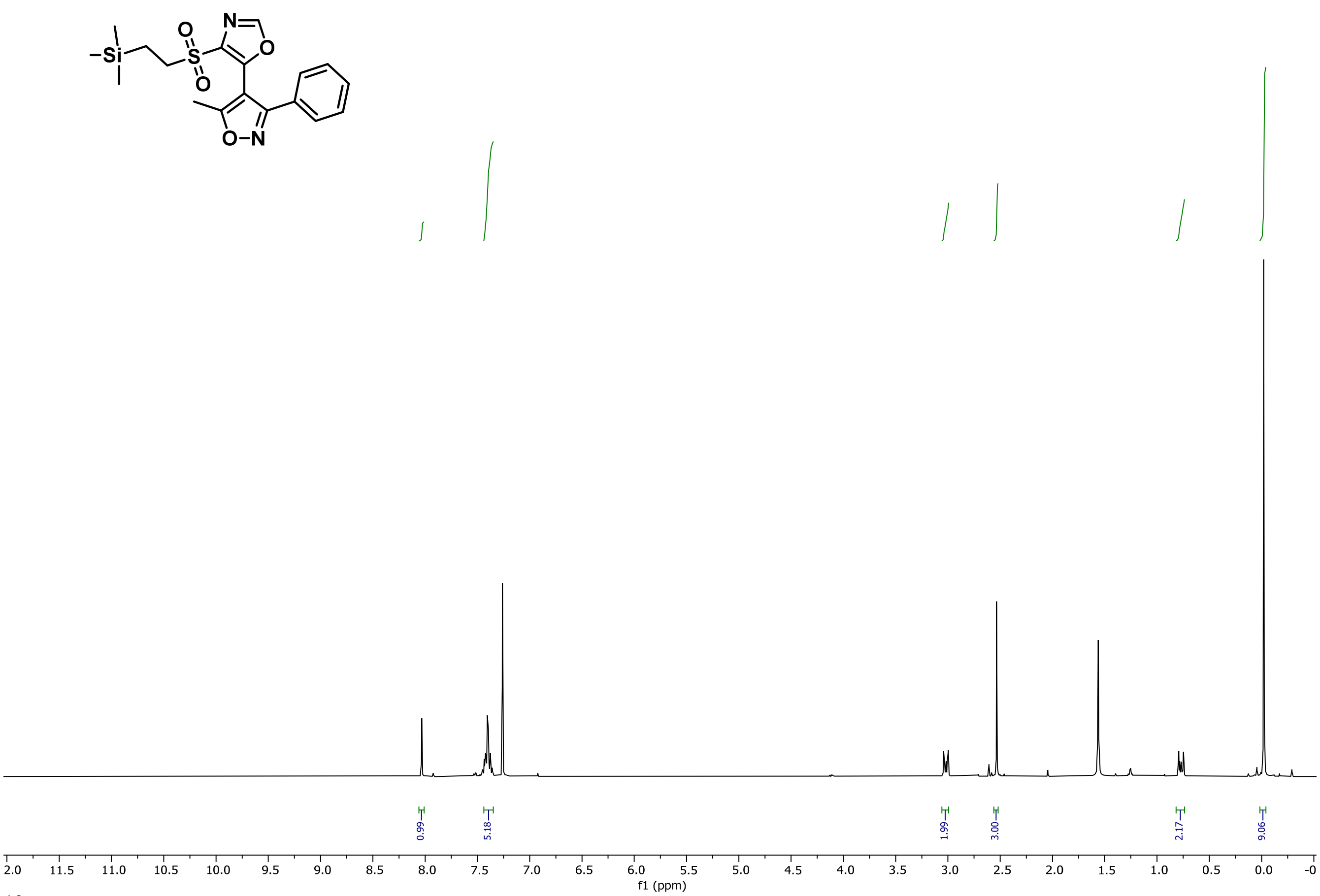

$^{13}C\{^{1}H\}$ ($CDCl_3$; 101 MHz) **3k**

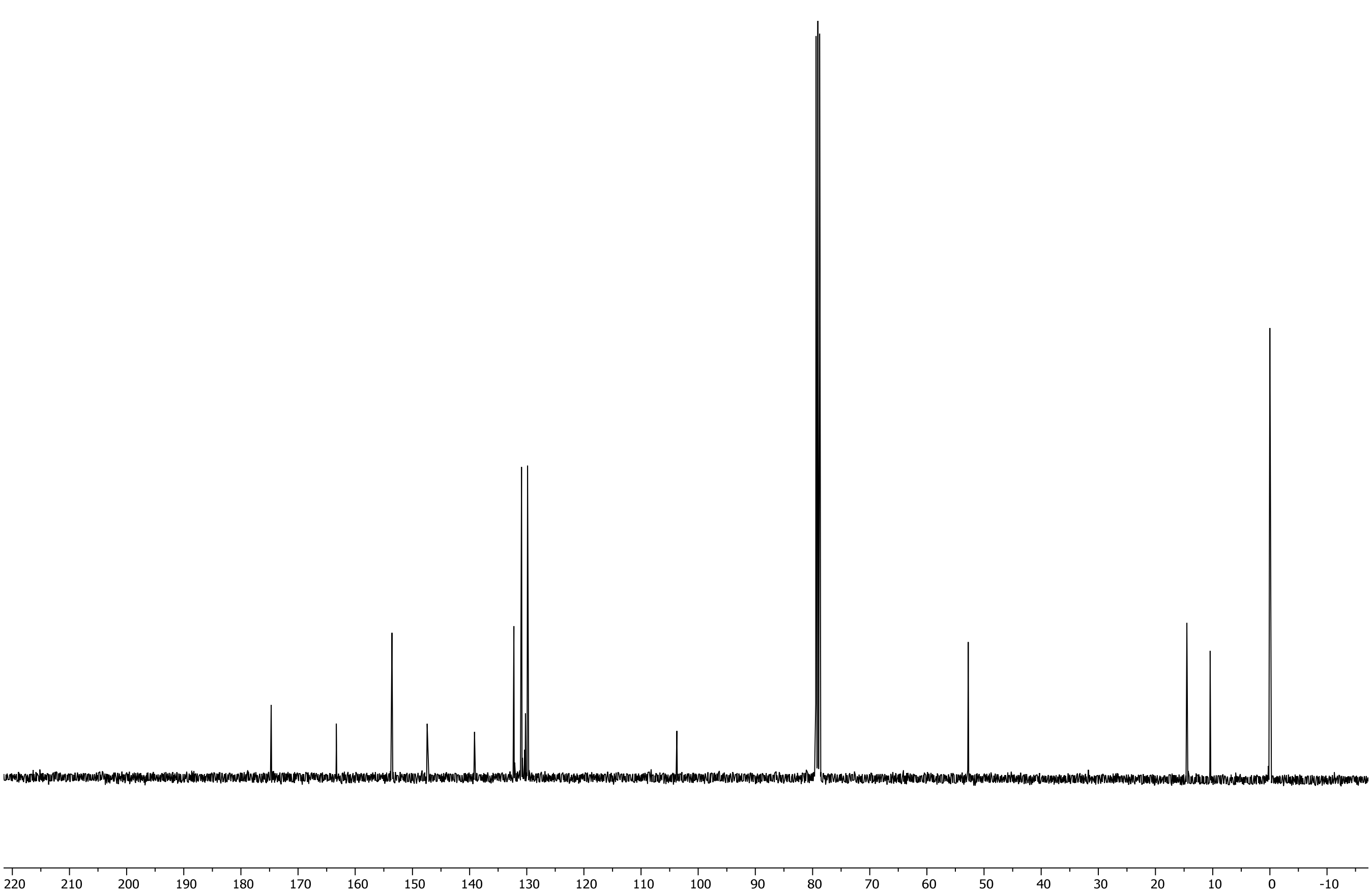

^{1}H (CDCl$_3$, 400 MHz) **3l**

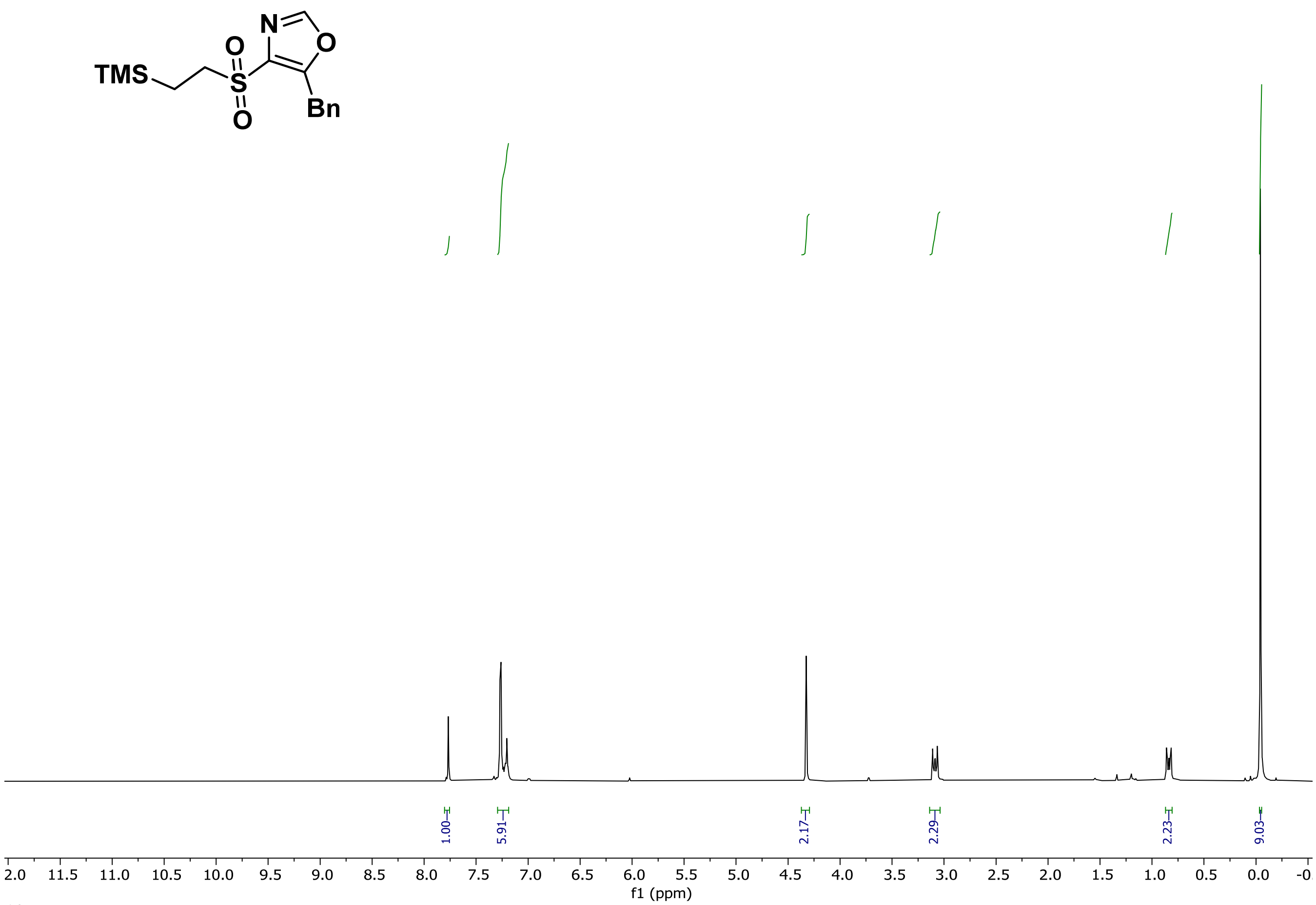

^{13}C{^{1}H} (CDCl$_3$; 101 MHz) **3l**

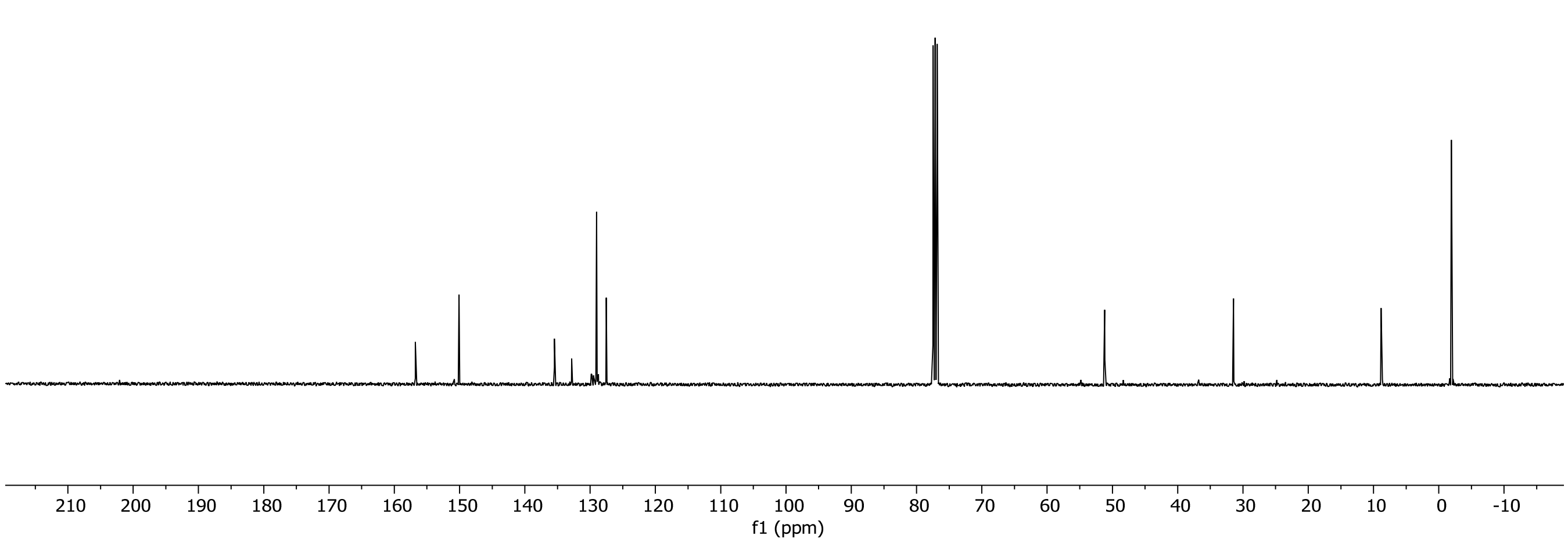

^{1}H (CDCl$_3$, 400 MHz) **3m**

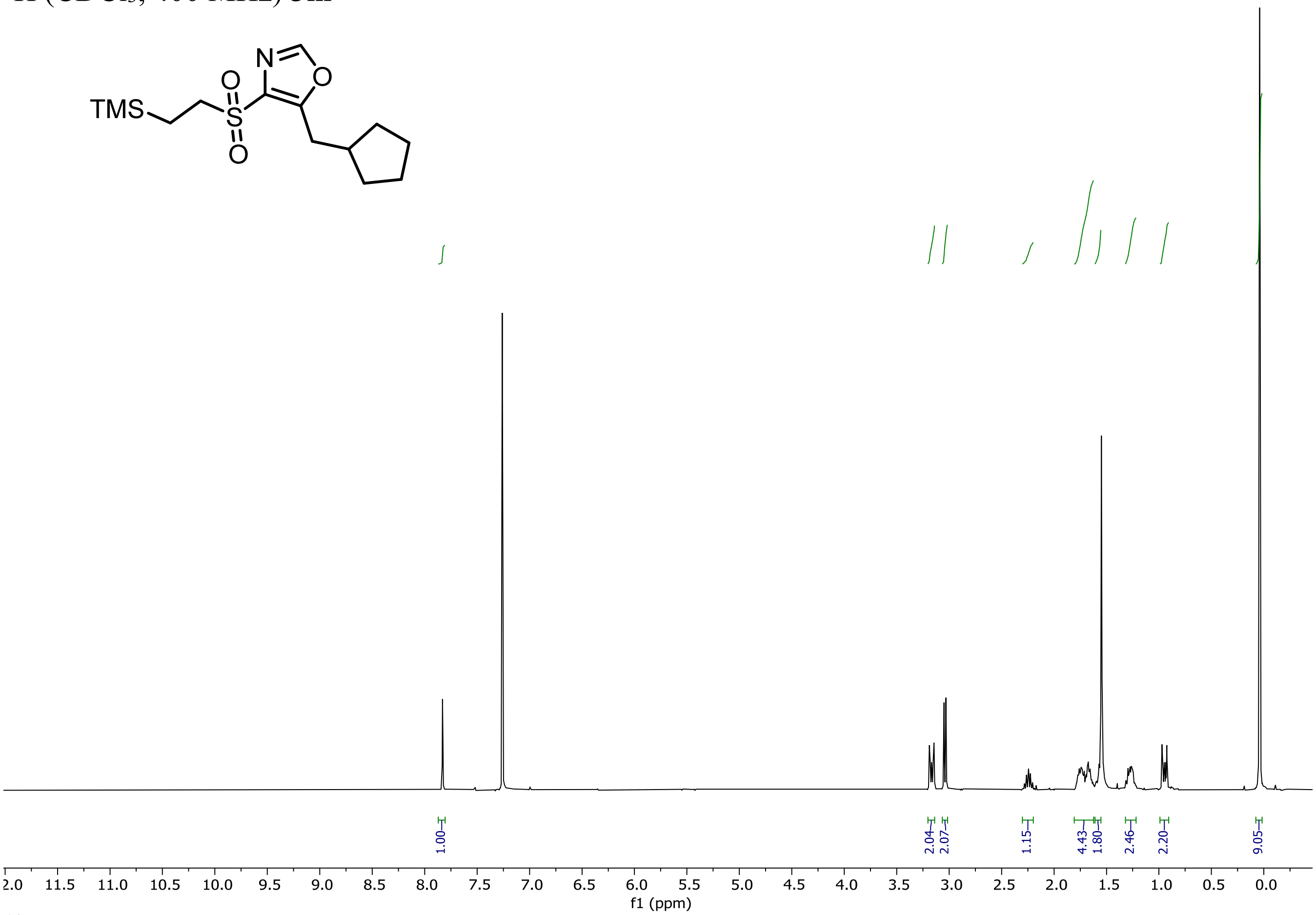

$^{13}C\{^{1}H\}$ (CDCl$_3$; 101 MHz) **3m**

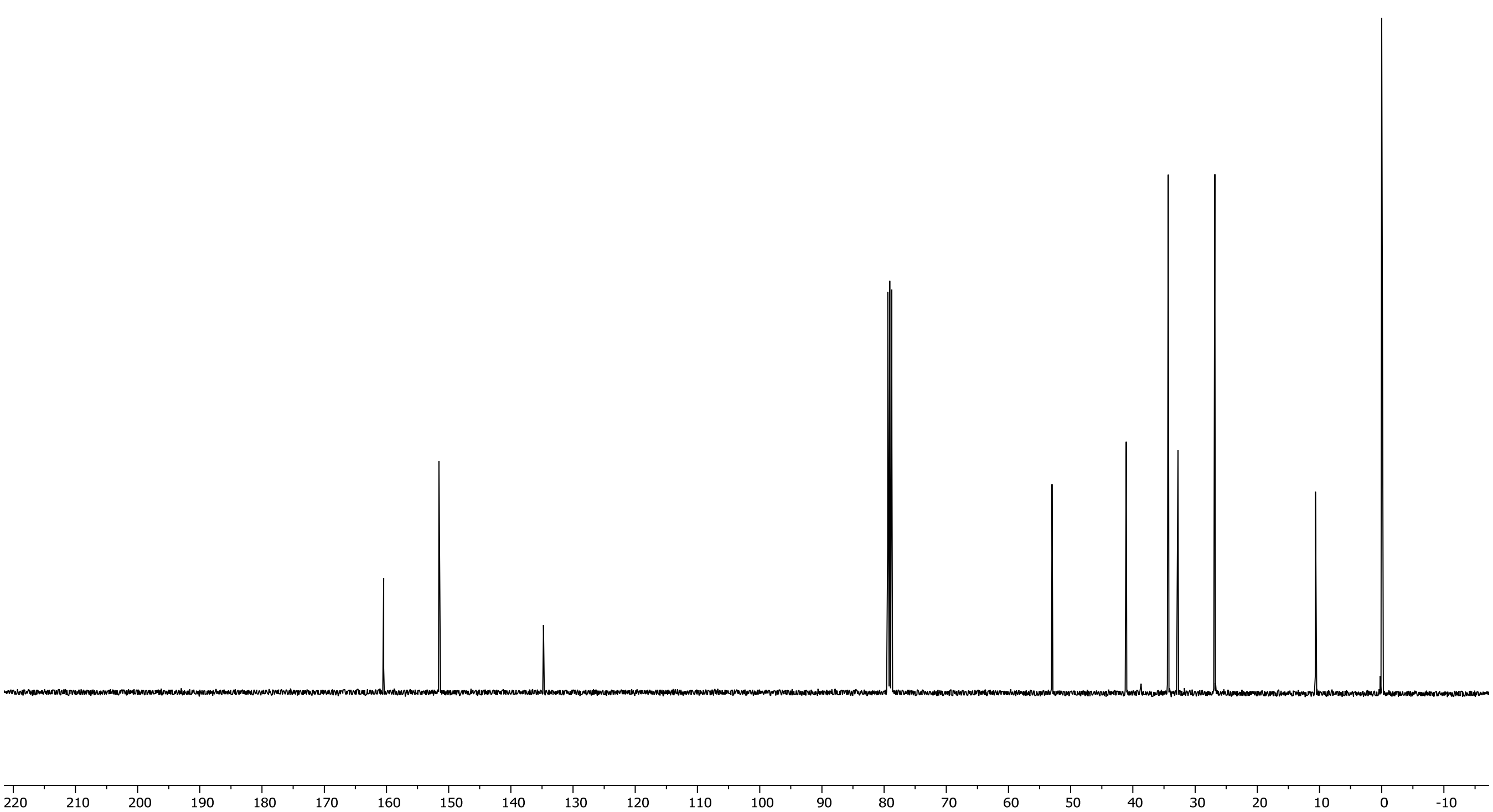

^{1}H ($CDCl_3$, 400 MHz) **3n**

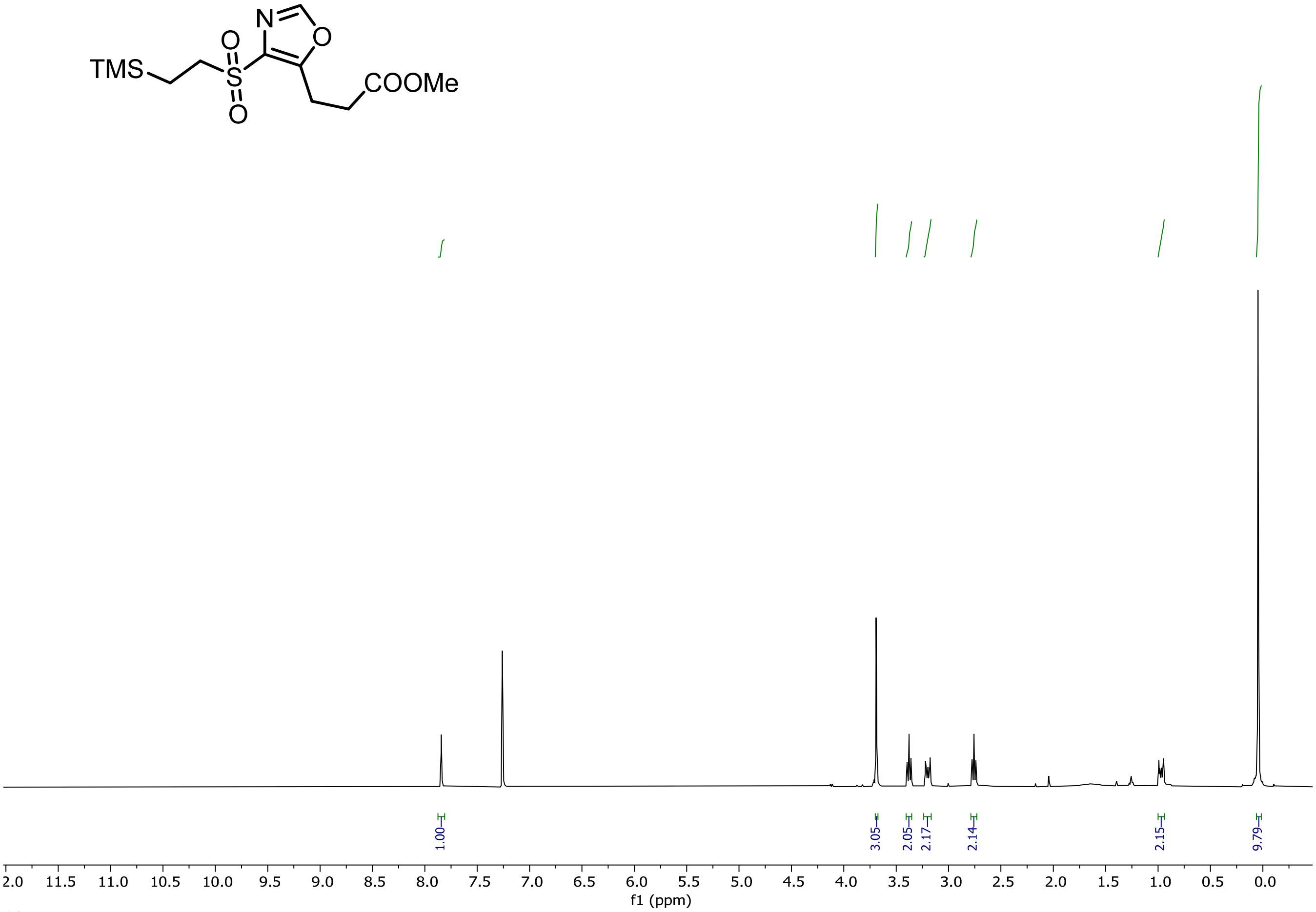

^{13}C{^{1}H} ($CDCl_3$; 101 MHz) **3n**

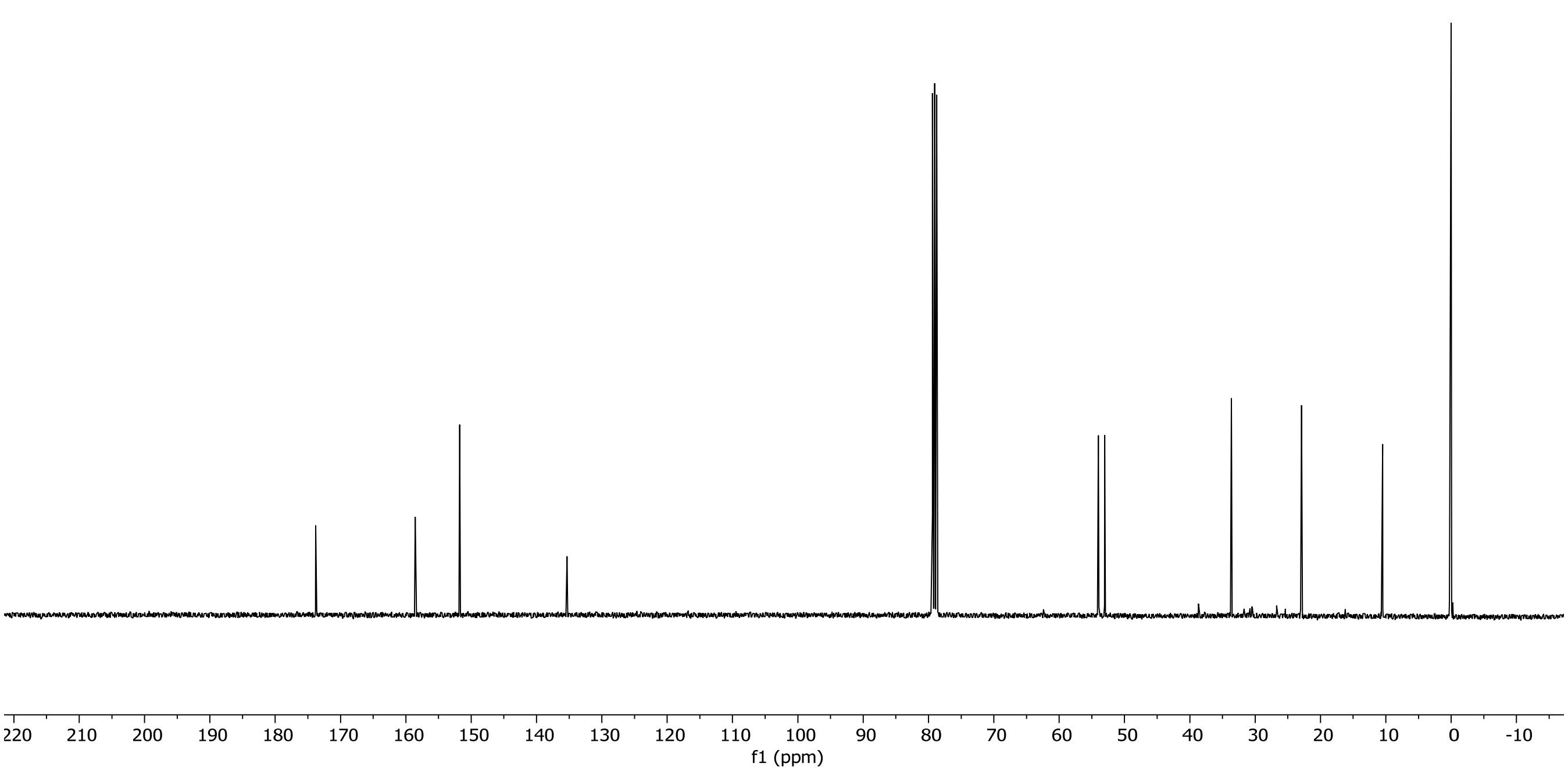

^{1}H ($CDCl_3$, 400 MHz) **3o**

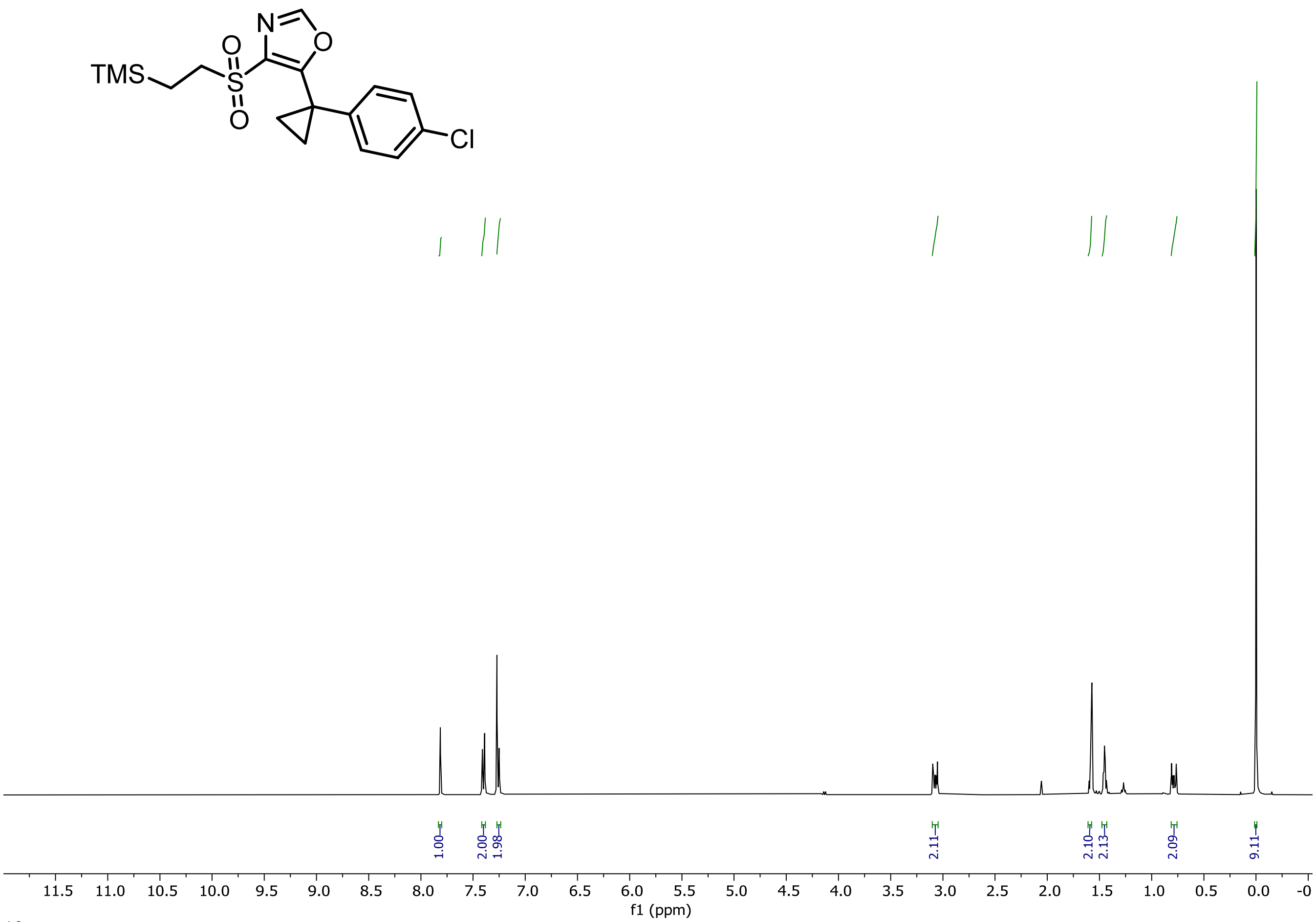

^{13}C{^{1}H} ($CDCl_3$; 101 MHz) **3o**

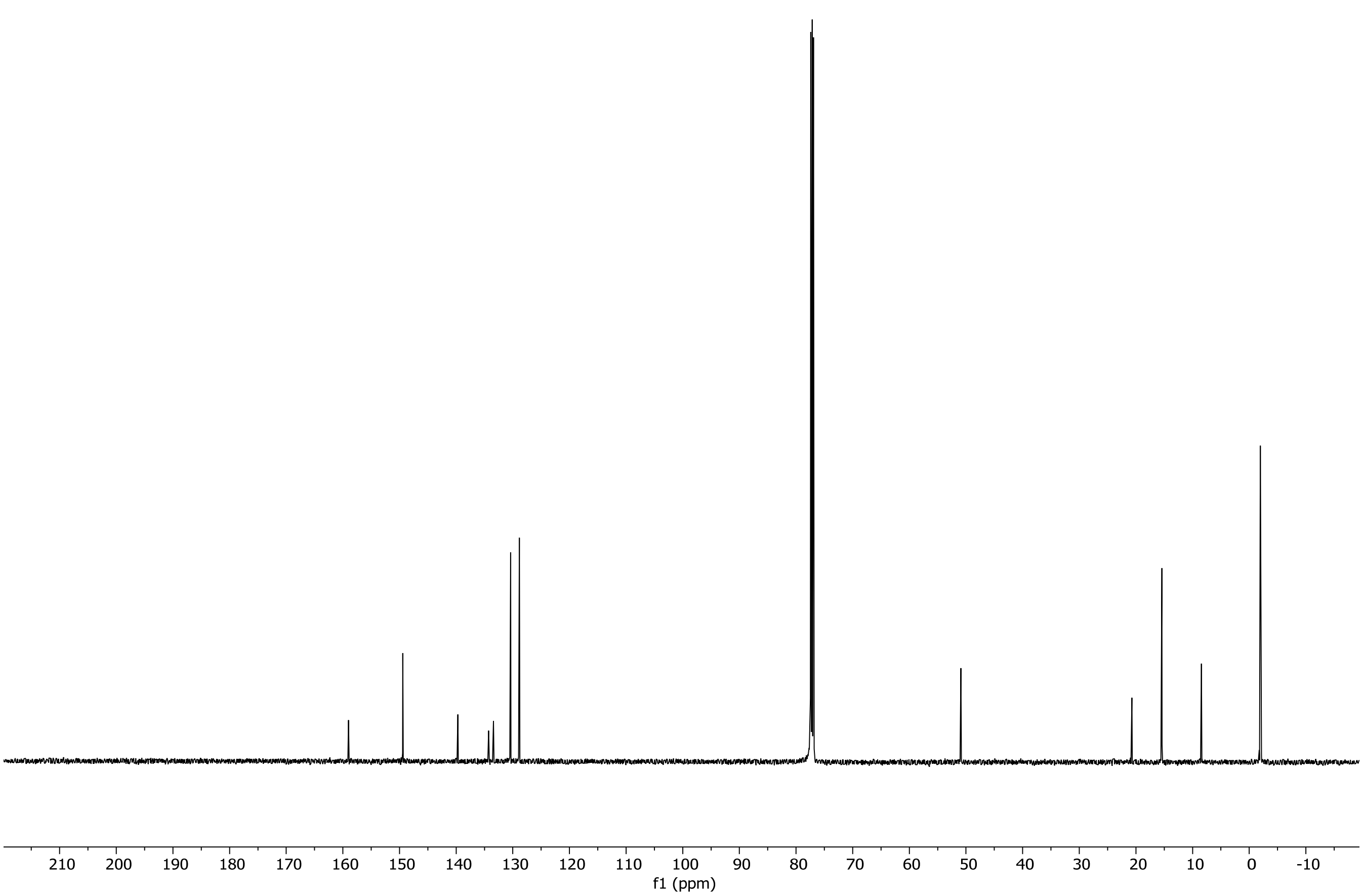

^{1}H ($CDCl_3$,400 MHz) **5**

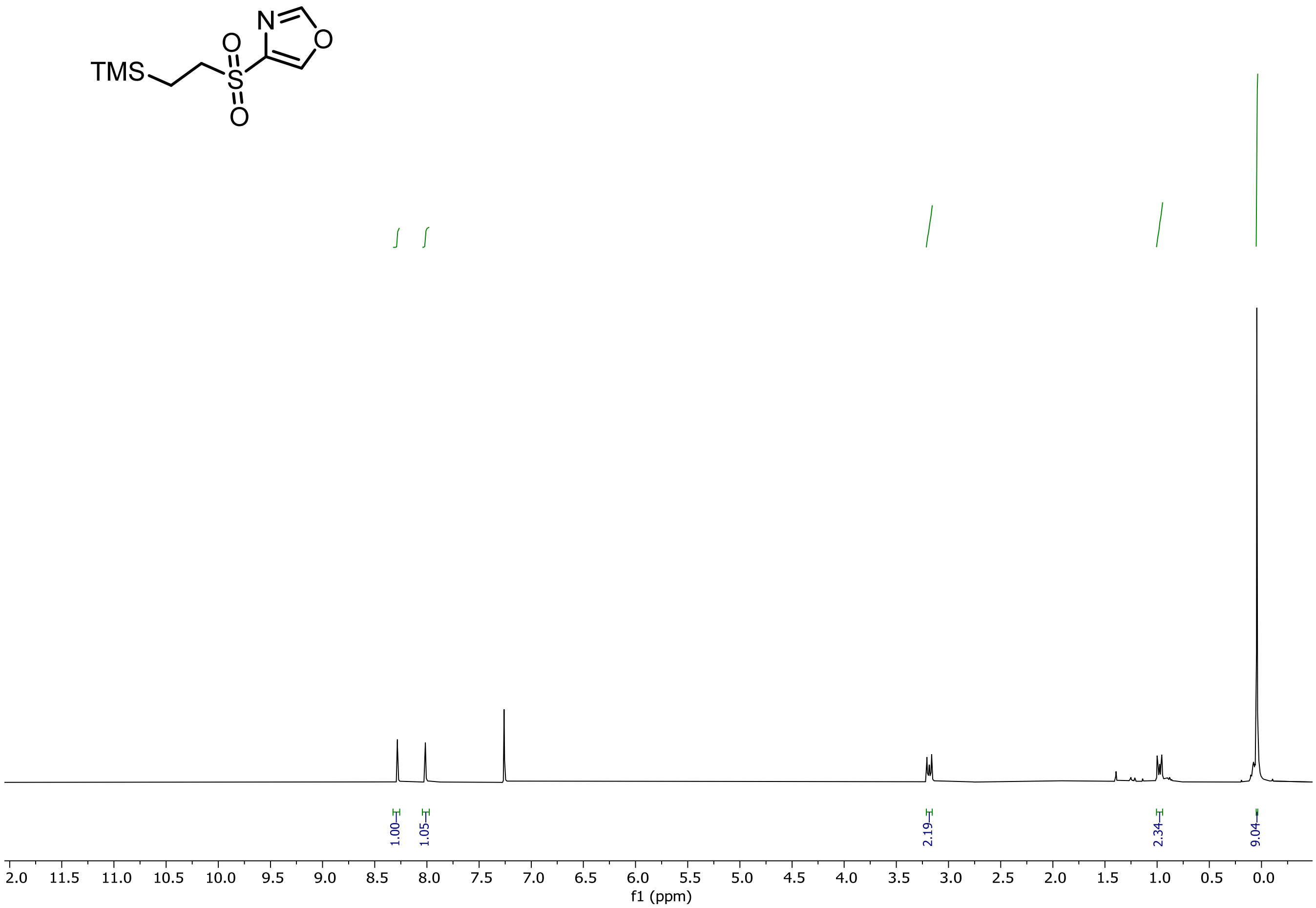

$^{13}C\{^{1}H\}$ ($CDCl_3$; 101 MHz) **5**

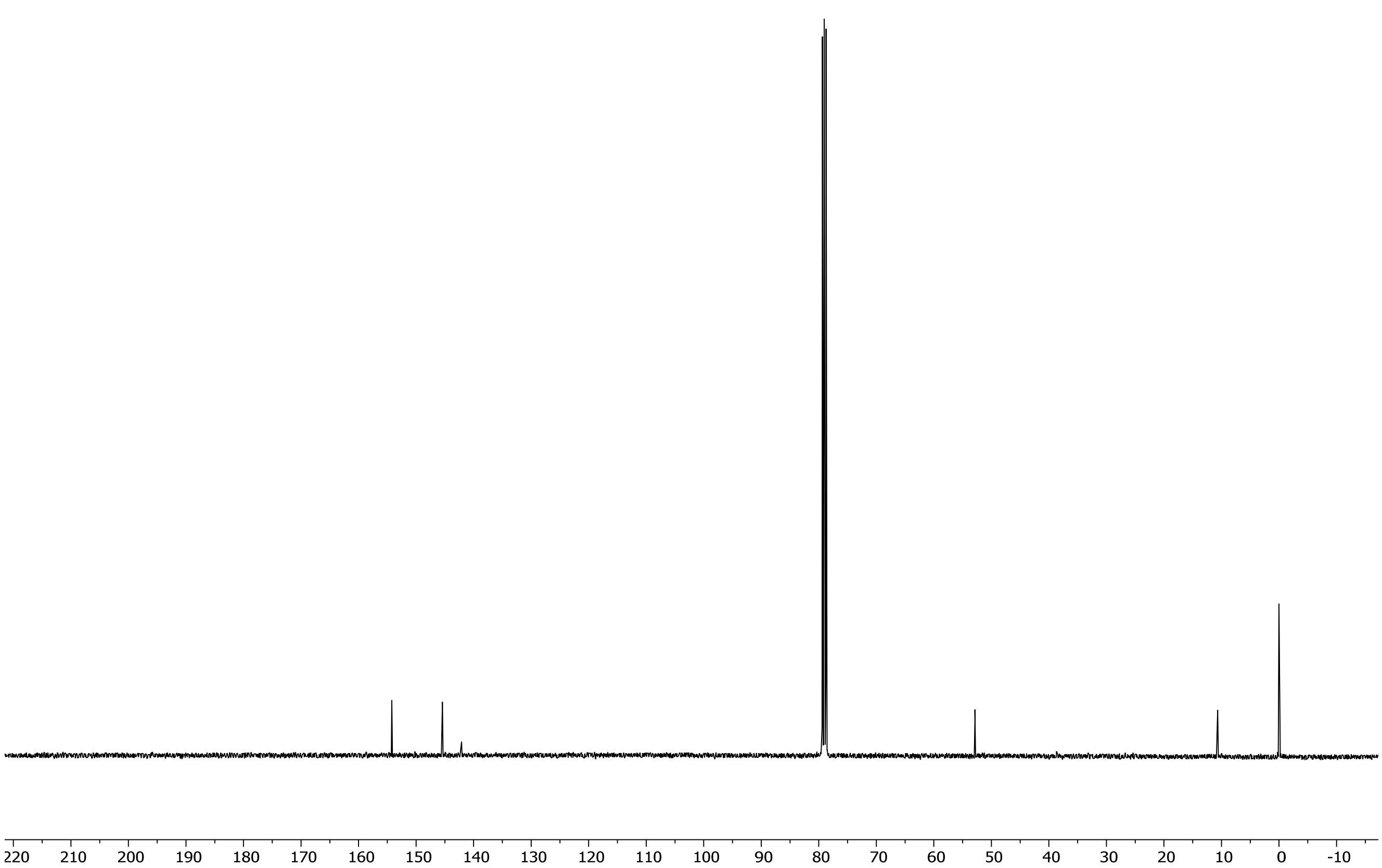

^{1}H (CDCl$_3$, 400 MHz) **6**

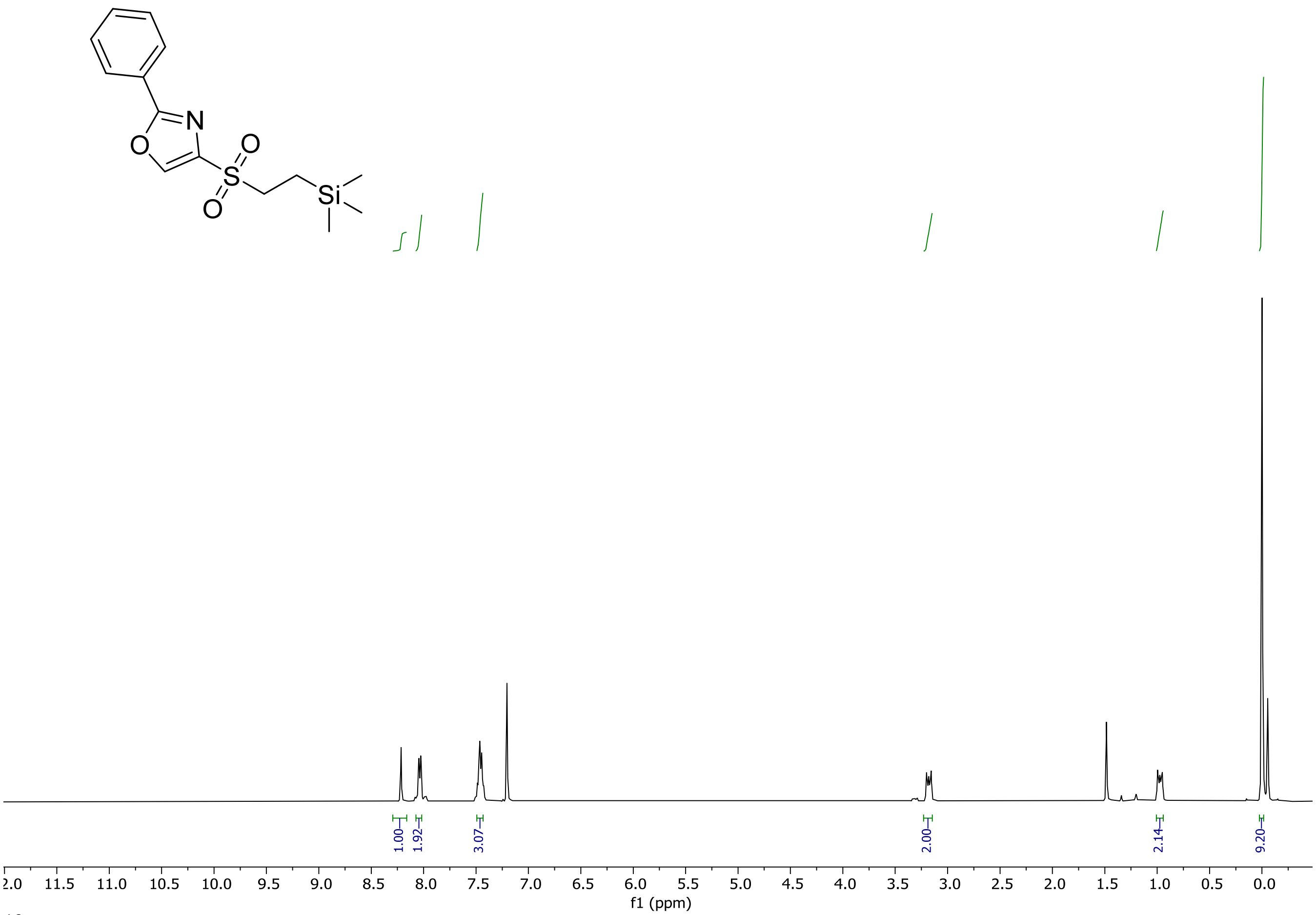

$^{13}C\{^{1}H\}$ (CDCl$_3$; 126 MHz) **6**

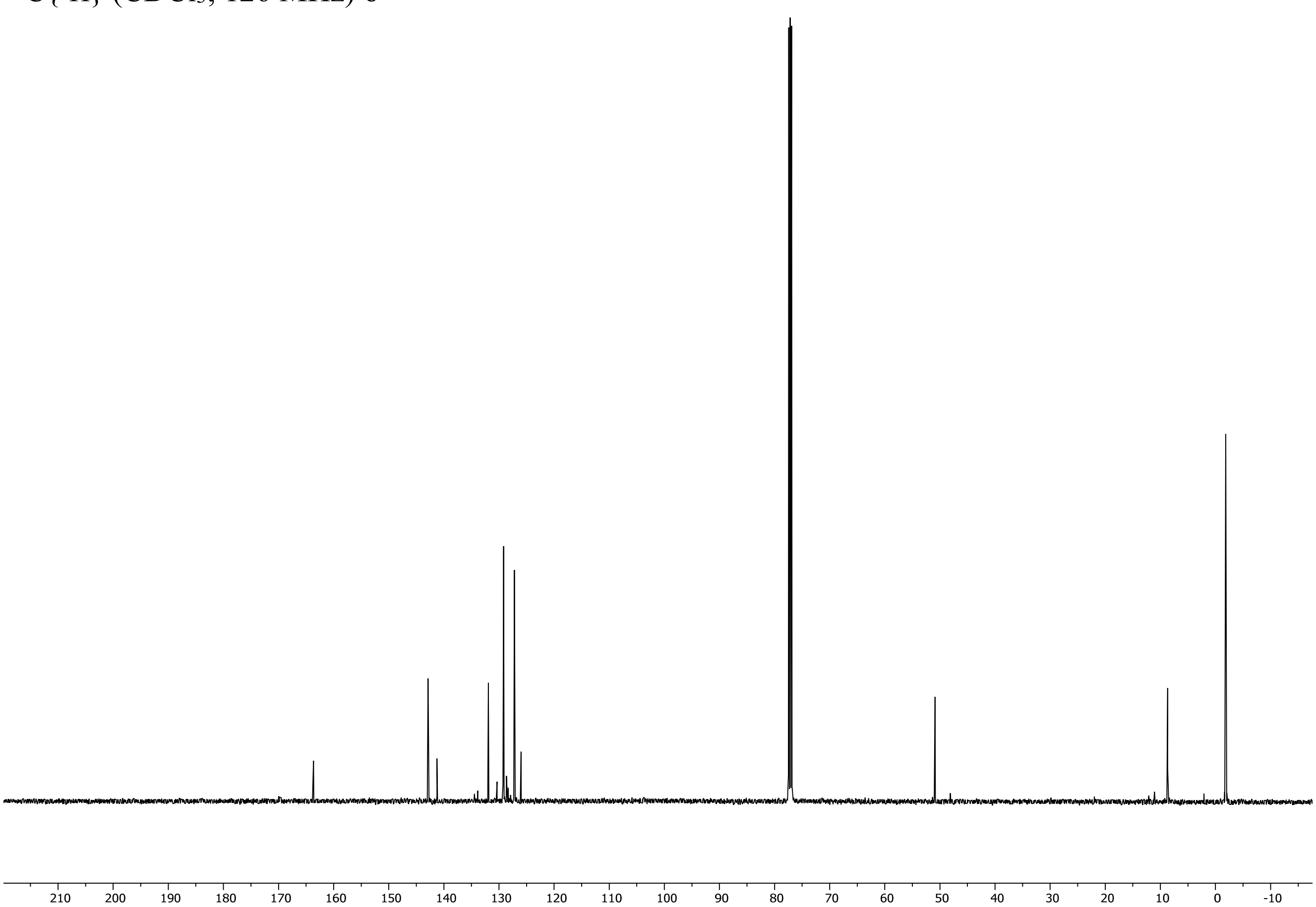

^{1}H ($CDCl_3$, 400 MHz) **7**

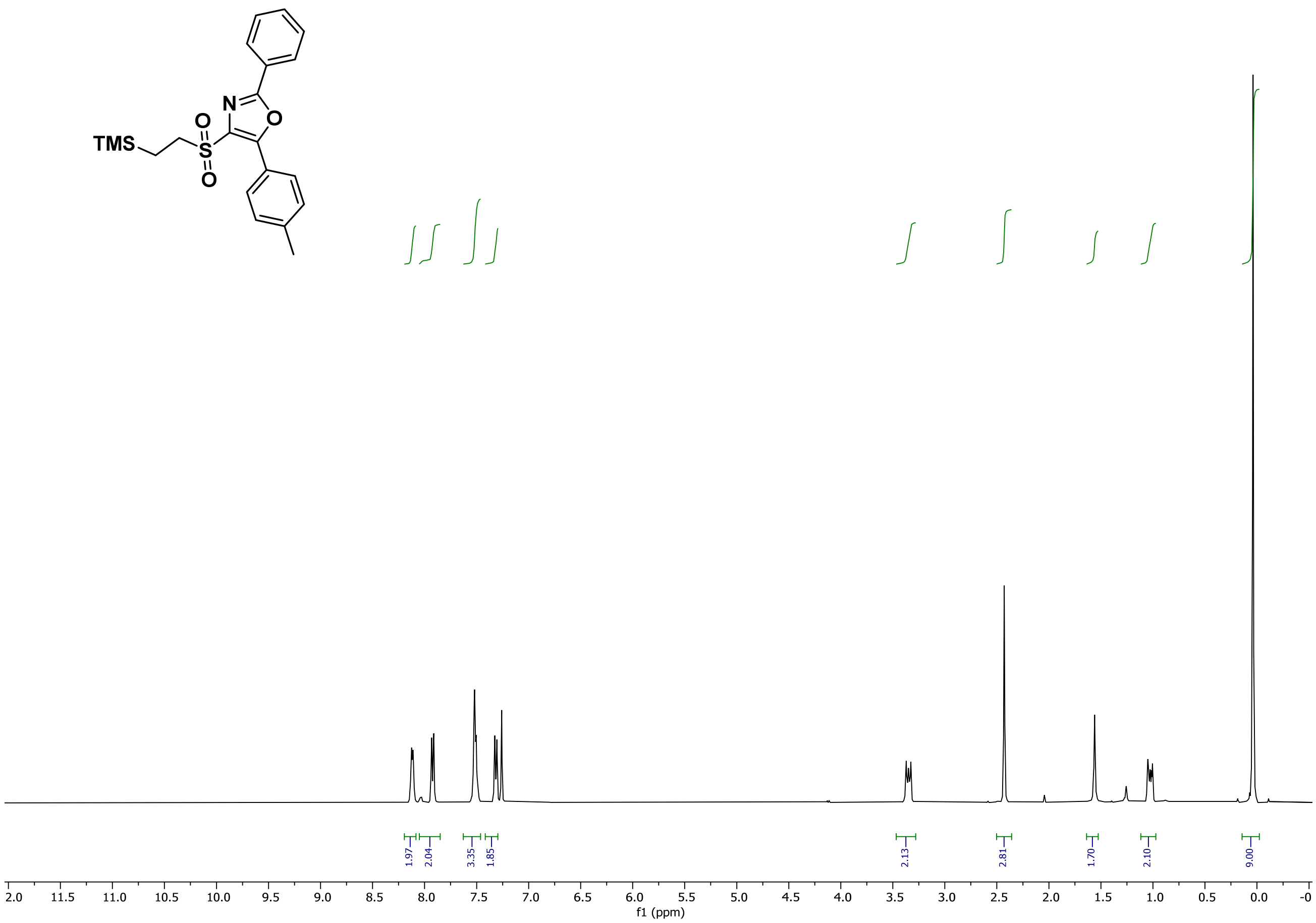

^{13}C{^{1}H} ($CDCl_3$; 126 MHz) **7**

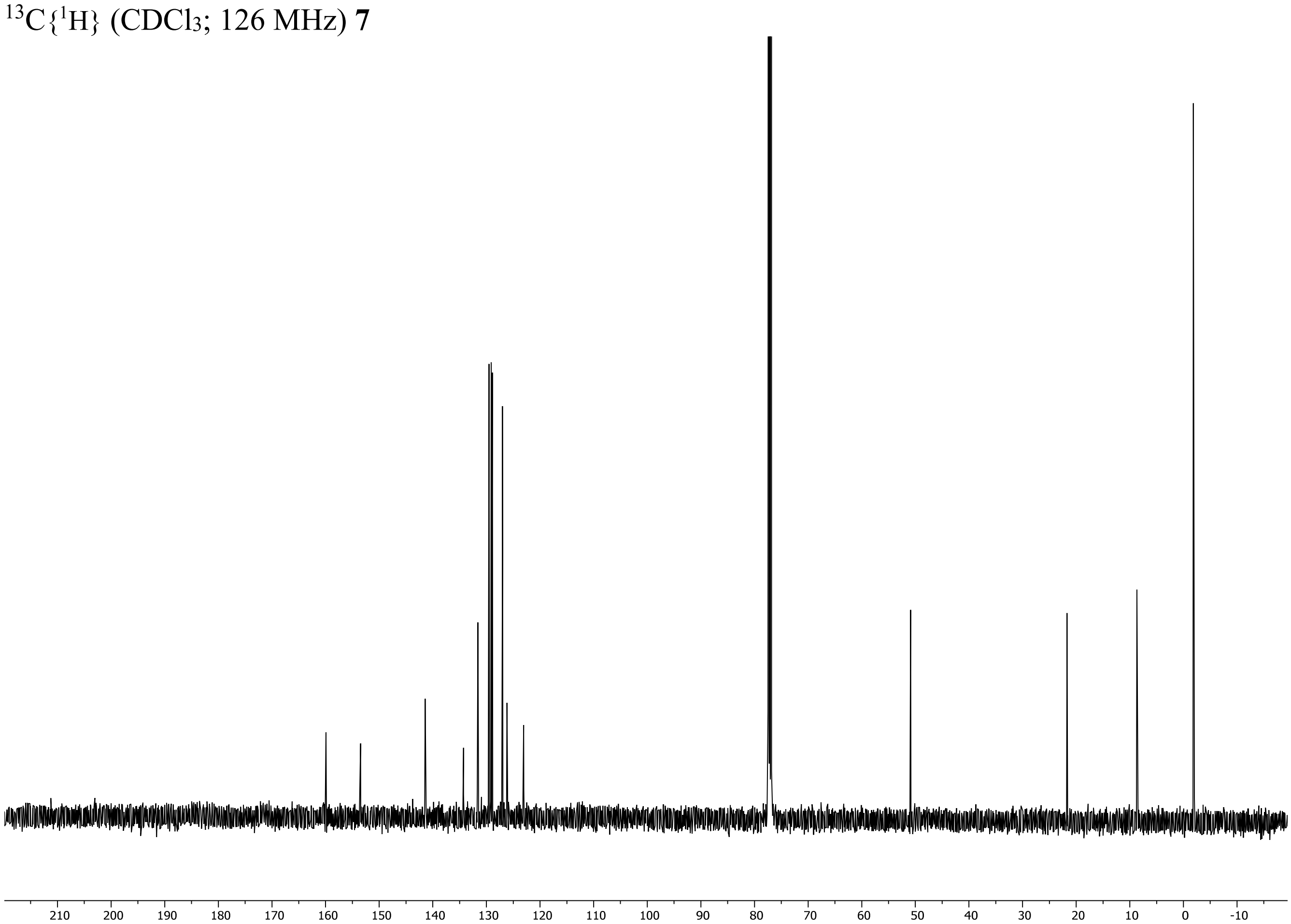

^{1}H (CDCl₃, 400 MHz) **9**

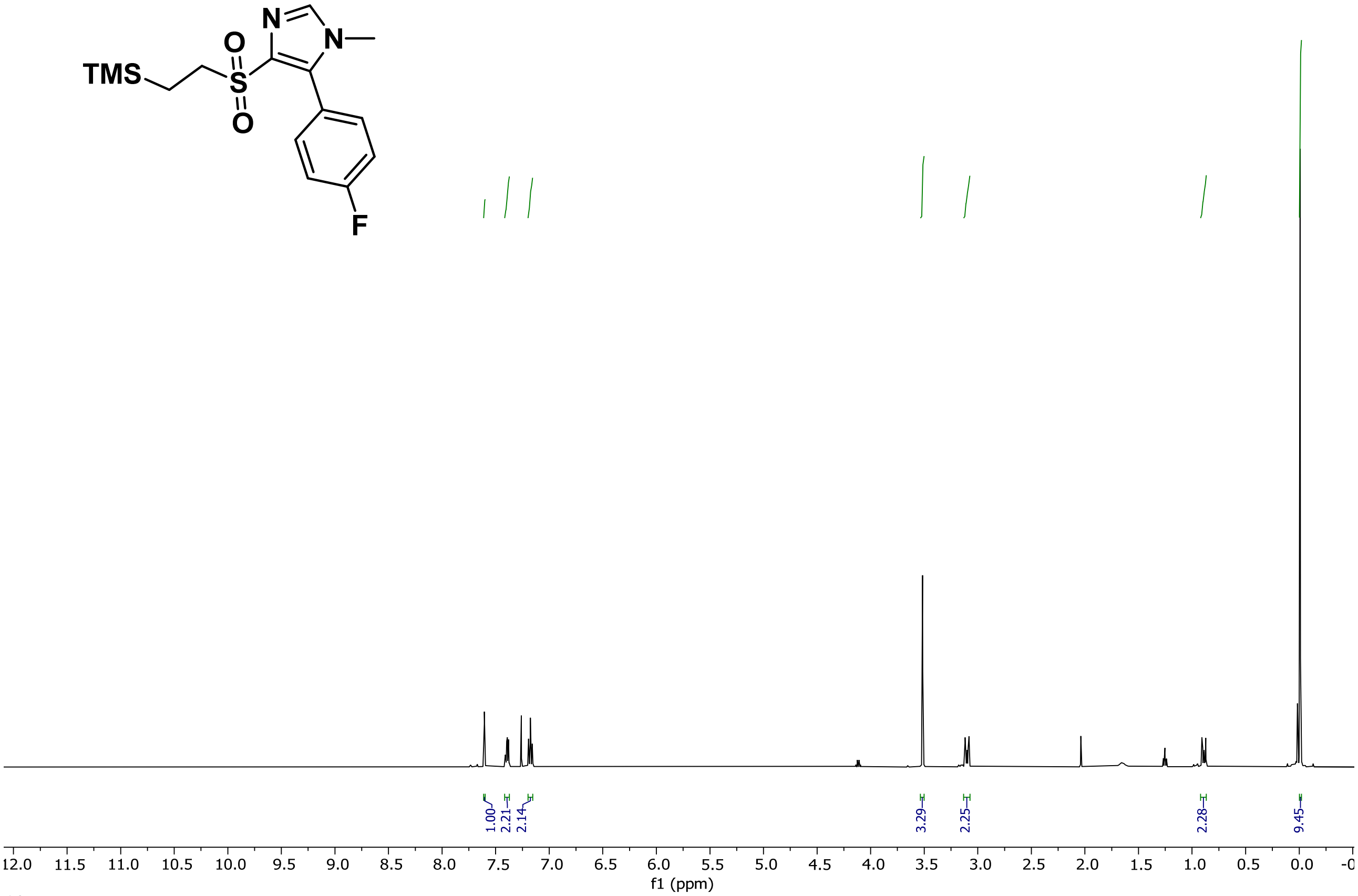

$^{13}C\{^{1}H\}$ (CDCl₃; 126 MHz) **9**

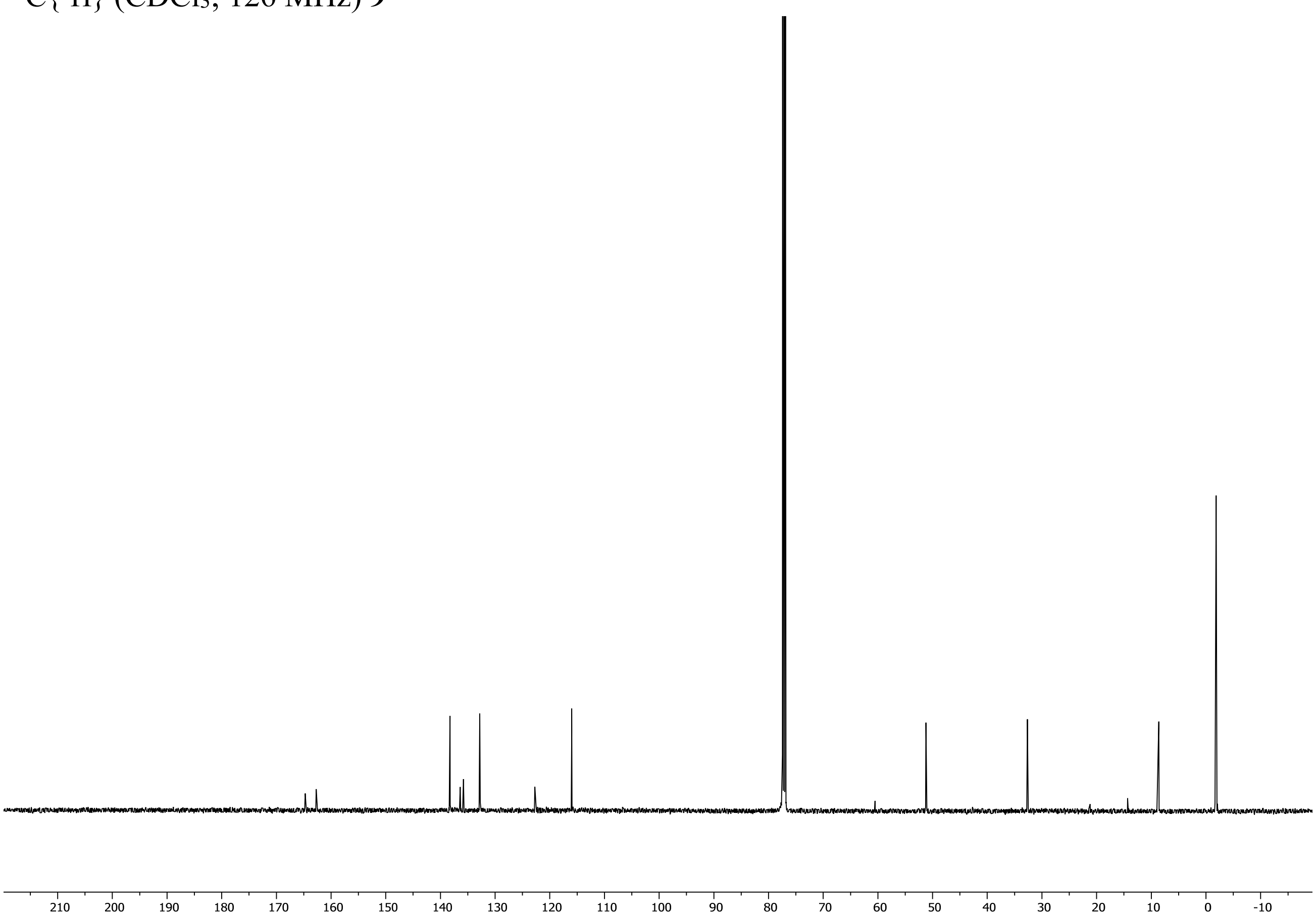

*Note: 9 is unstable and will deuterate at the 2-position if concentrated from $CDCl_3$ or left for long periods of time.

$^{19}F\{^{1}H, ^{13}C\}$ ($CDCl_3$, 376 MHz) **9**

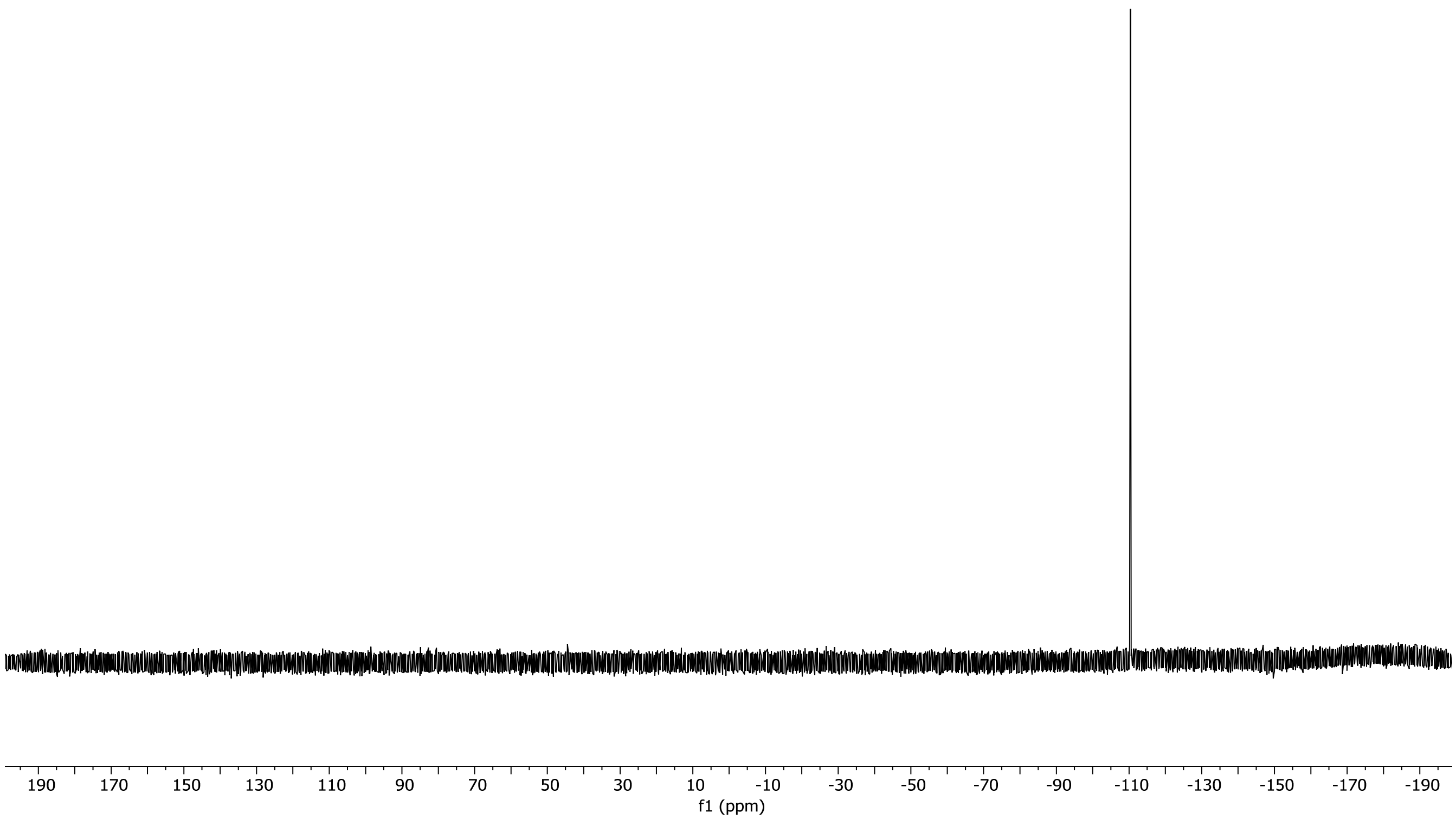

^{1}H ($CDCl_3$, 400 MHz) **11**

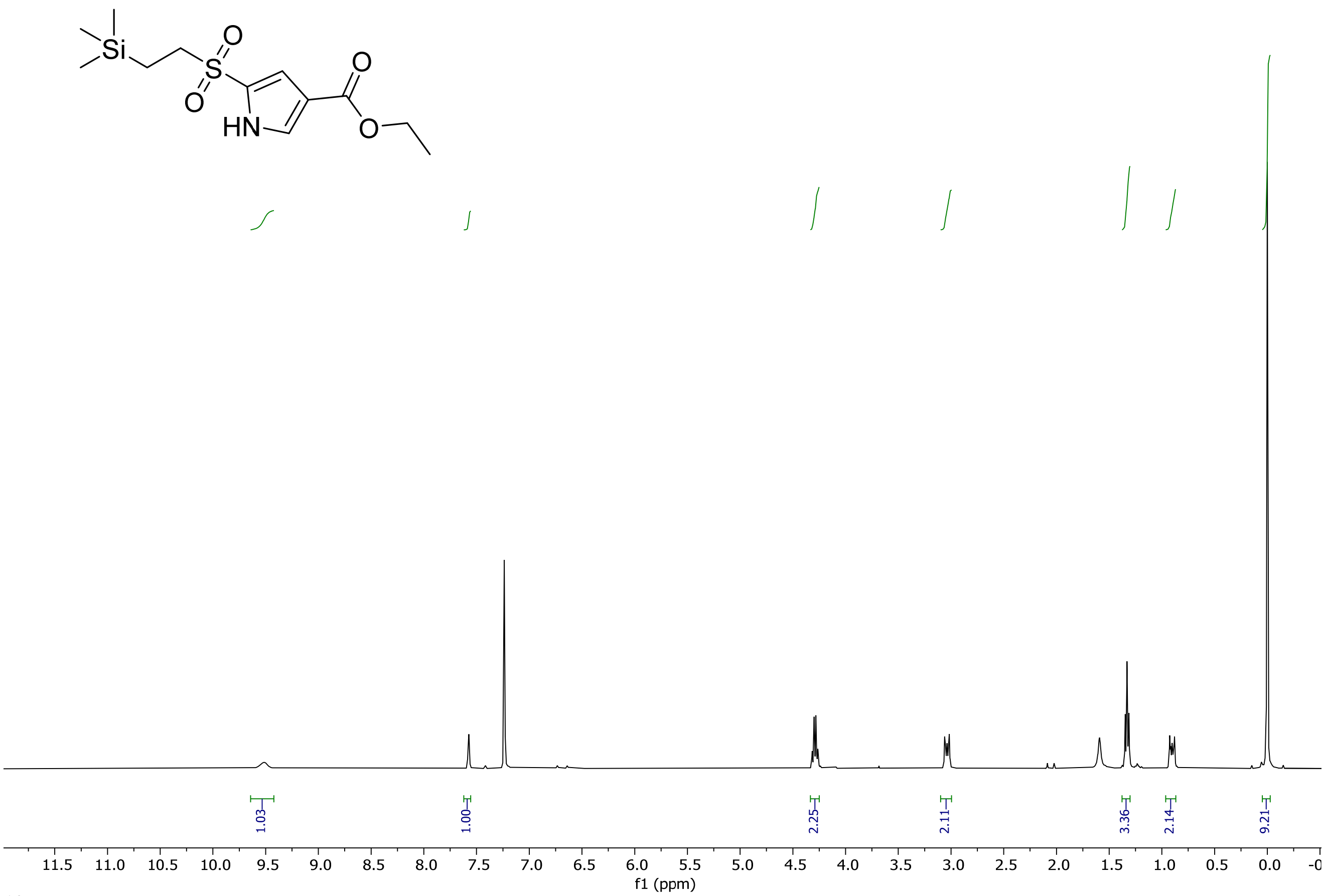

$^{13}C\{^{1}H\}$ ($CDCl_3$; 101 MHz) **11**

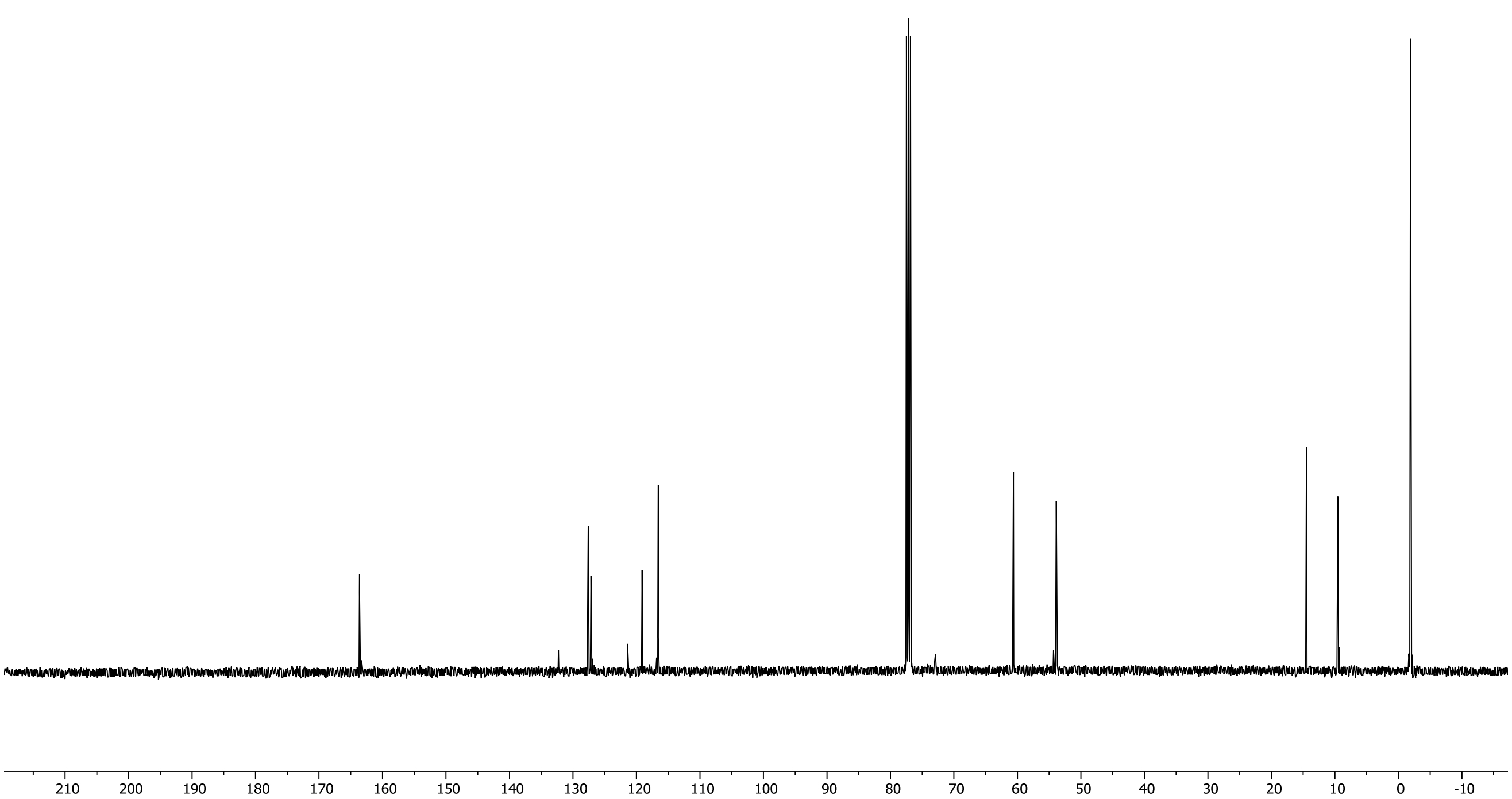

^{1}H ($CDCl_3$, 400 MHz) **13**

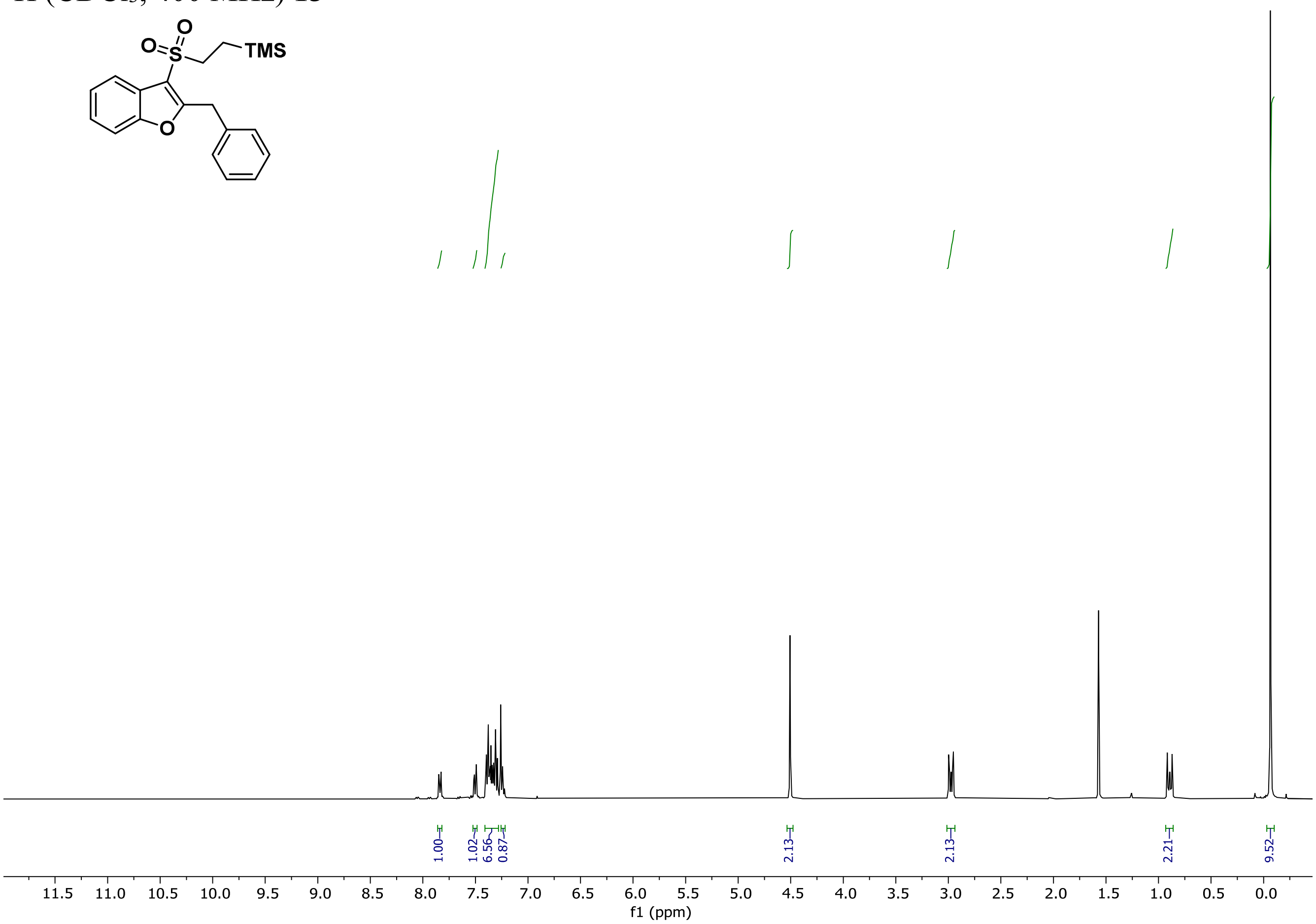

^{13}C{^{1}H} ($CDCl_3$; 101 MHz) **13**

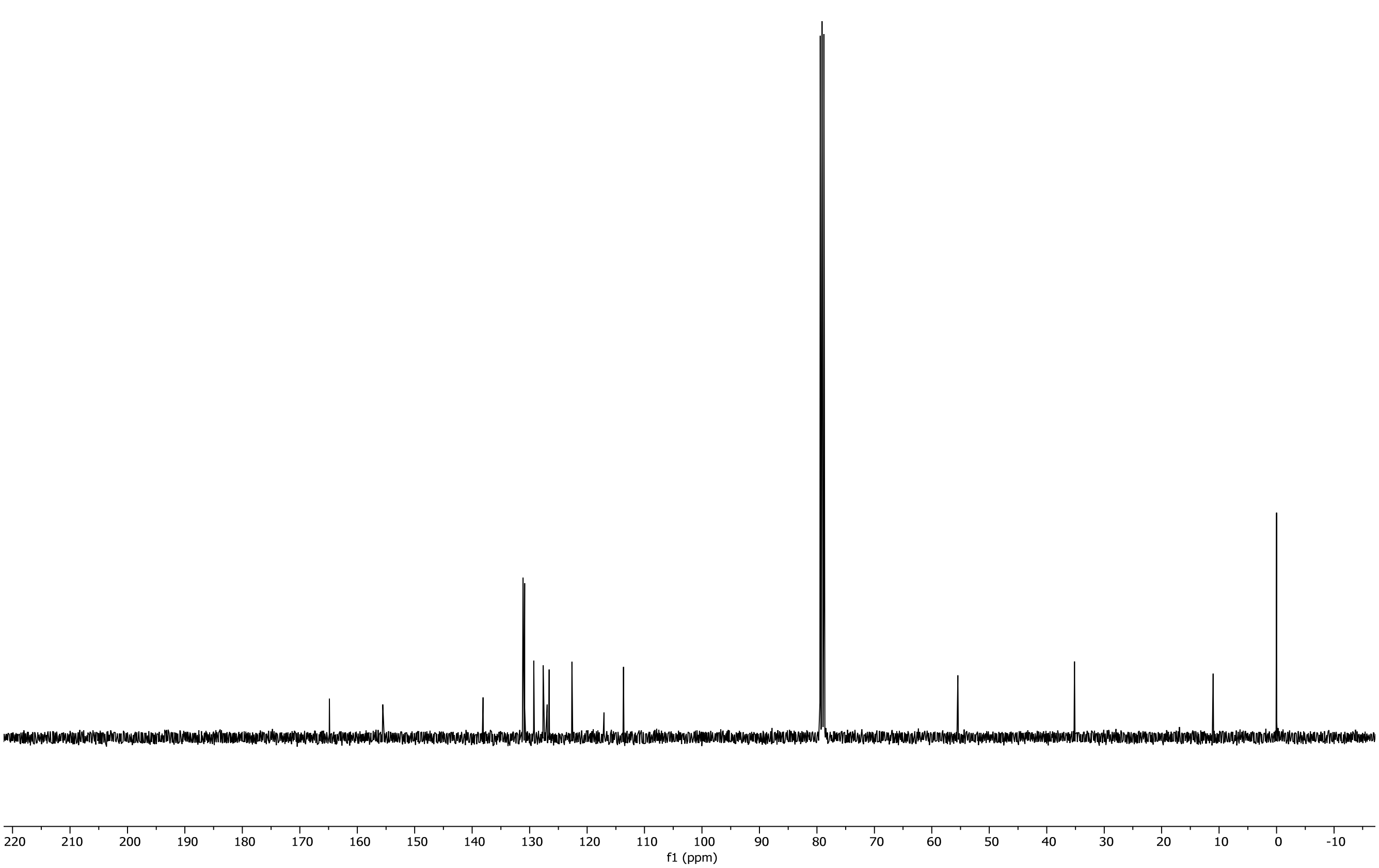

^{1}H ($CDCl_3$, 400 MHz) **15a**

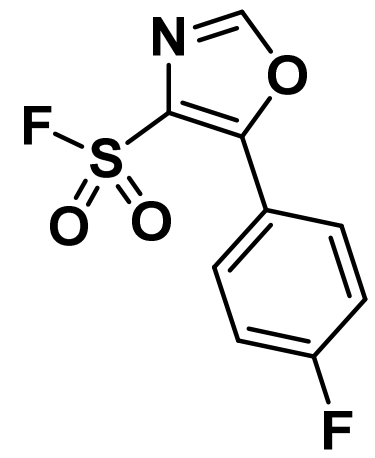

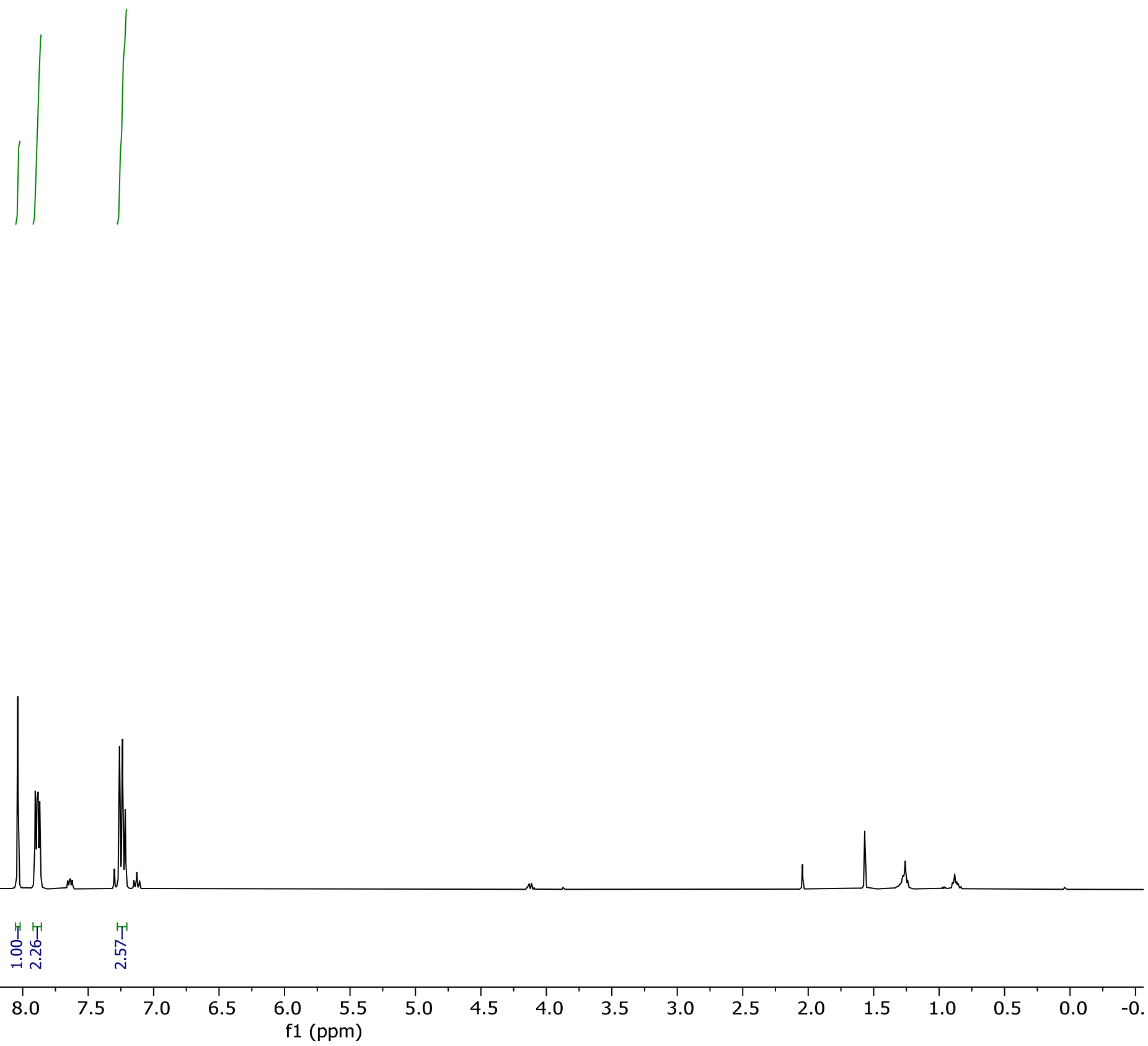

$^{13}C\{^{1}H\}$ (126 MHz, C_6D_6) **15a**

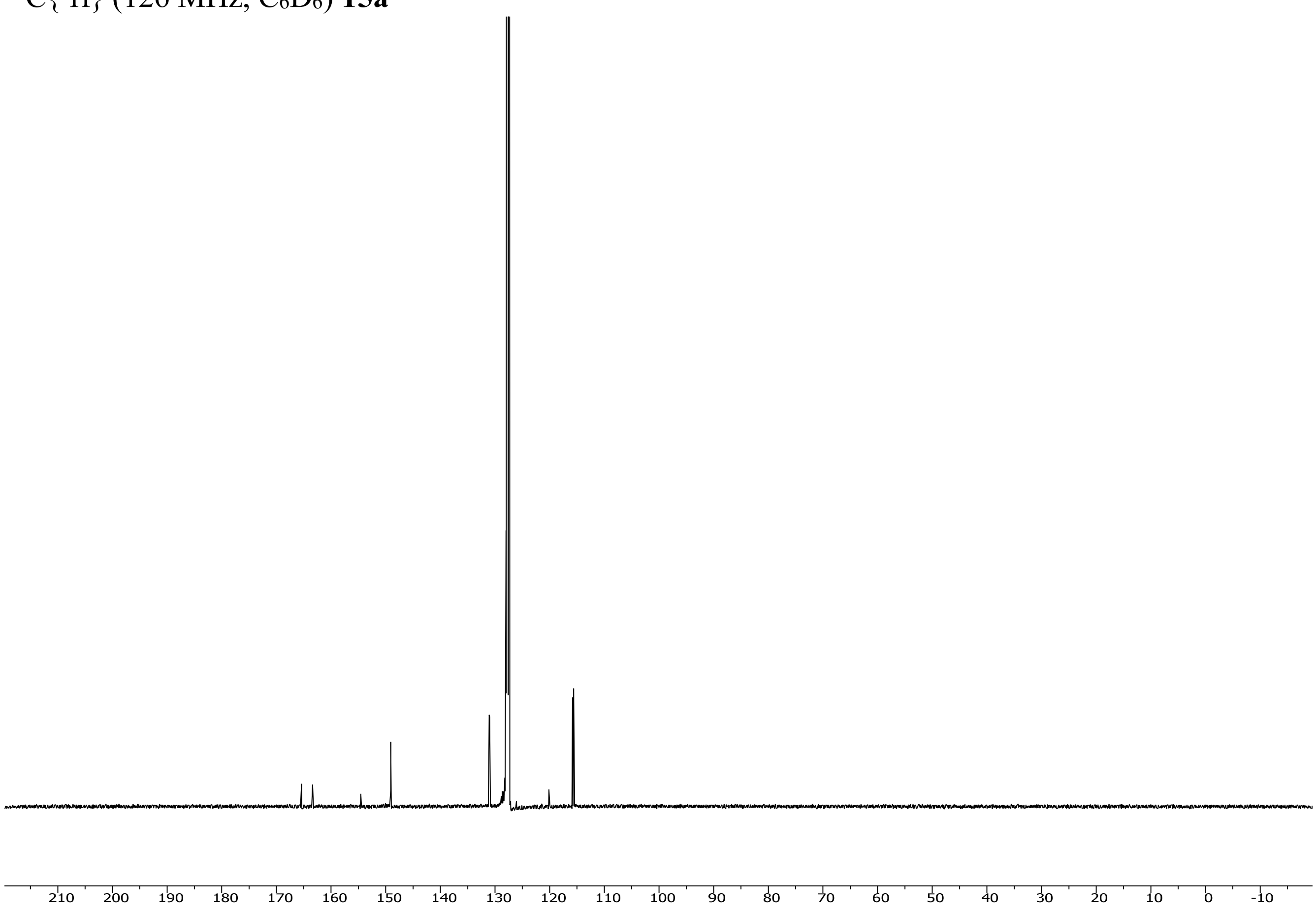

$^{19}F\{^{1}H, ^{13}C\}$ ($CDCl_3$, 376 MHz) **15a**

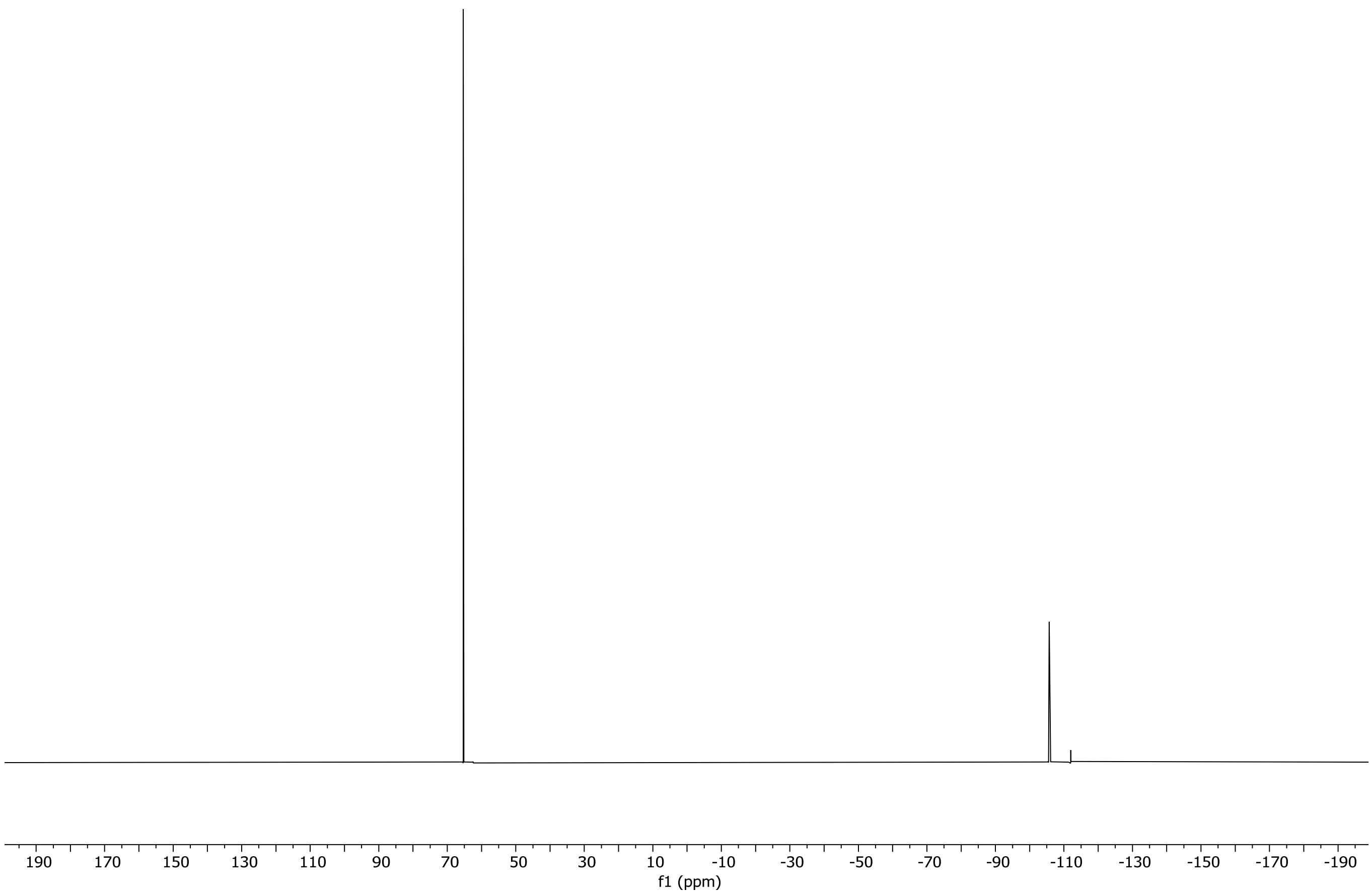

^{1}H (CDCl3, 400 MHz) **15c**

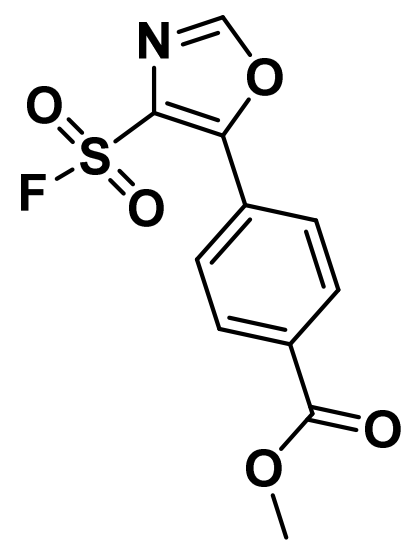

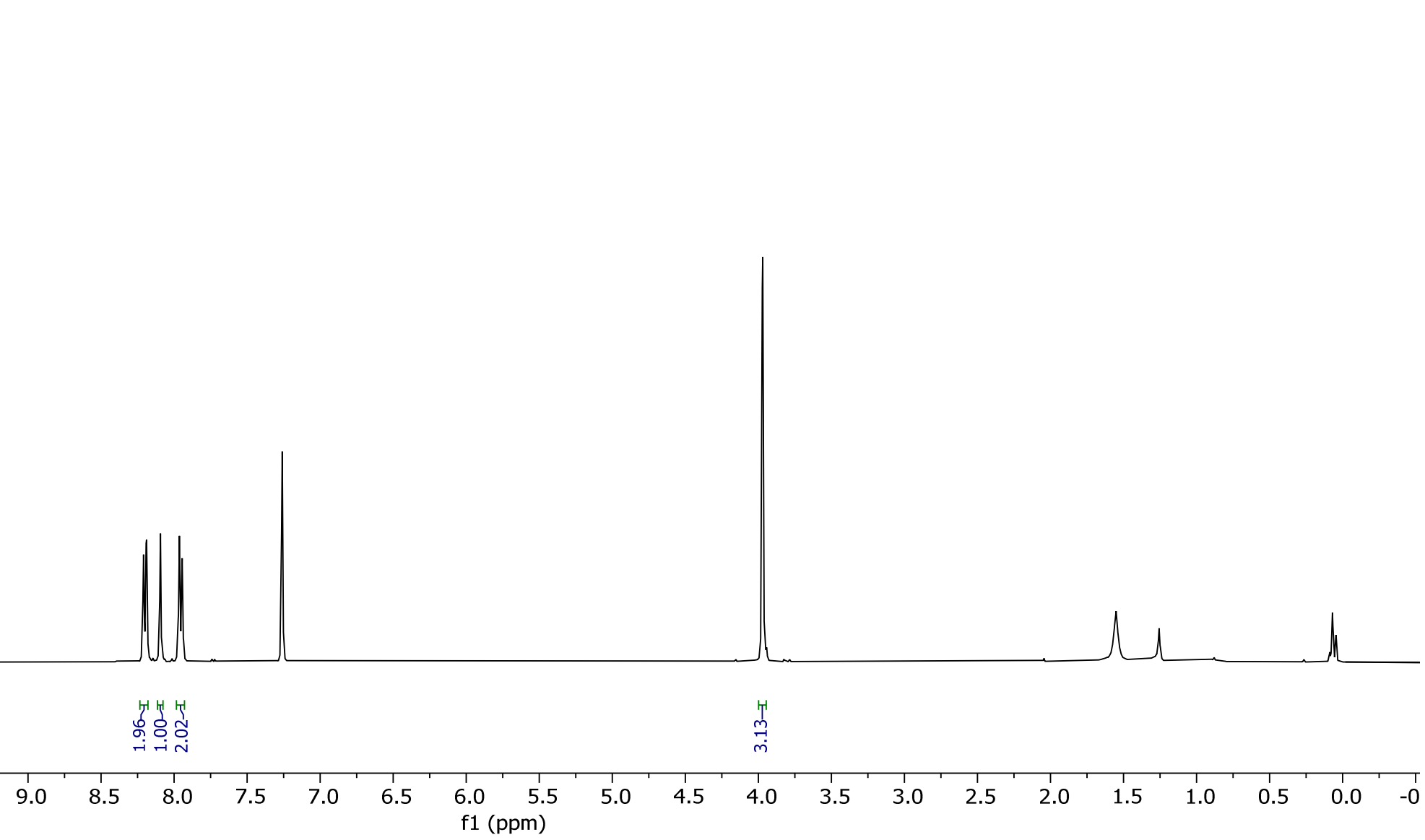

^{13}C{^{1}H} (CDCl3, 101 MHz) **15c**

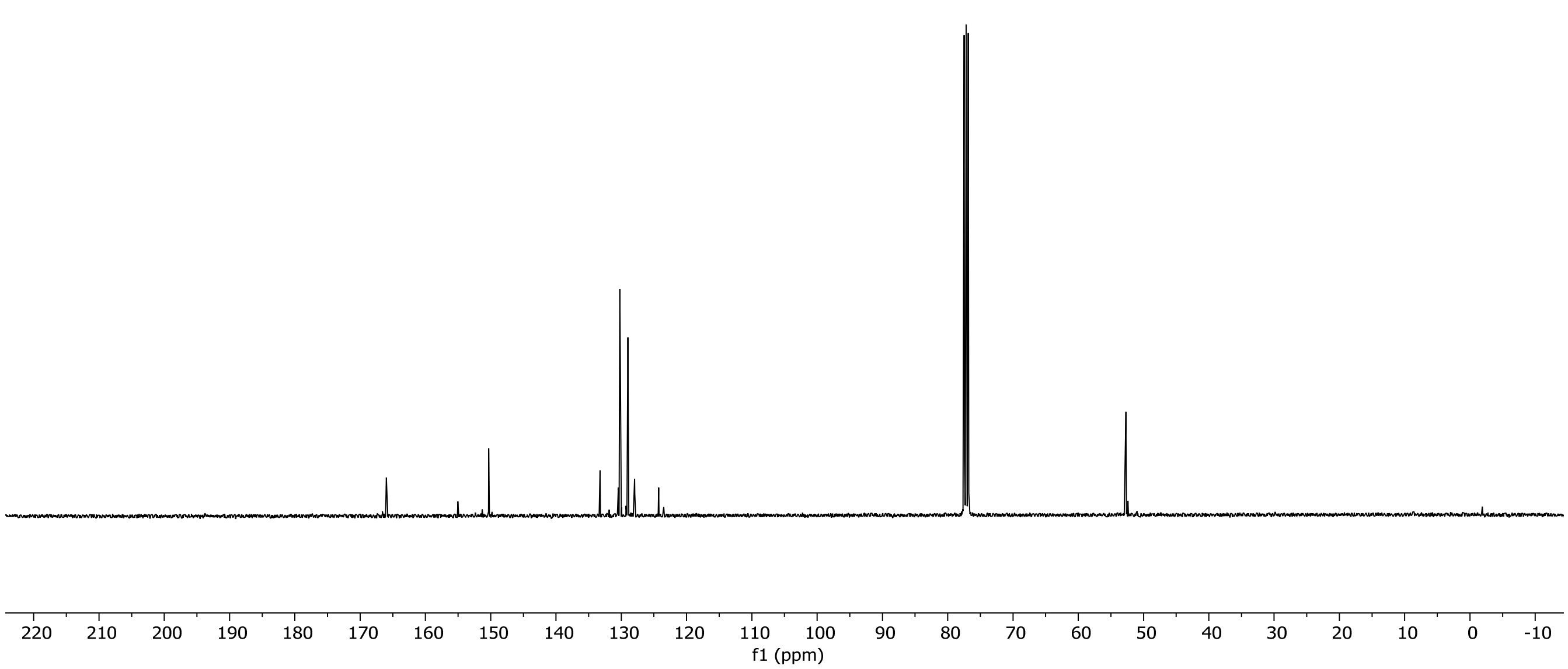

$^{19}F\{^1H, ^{13}C\}$ (CDCl₃, 376 MHz) **15c**

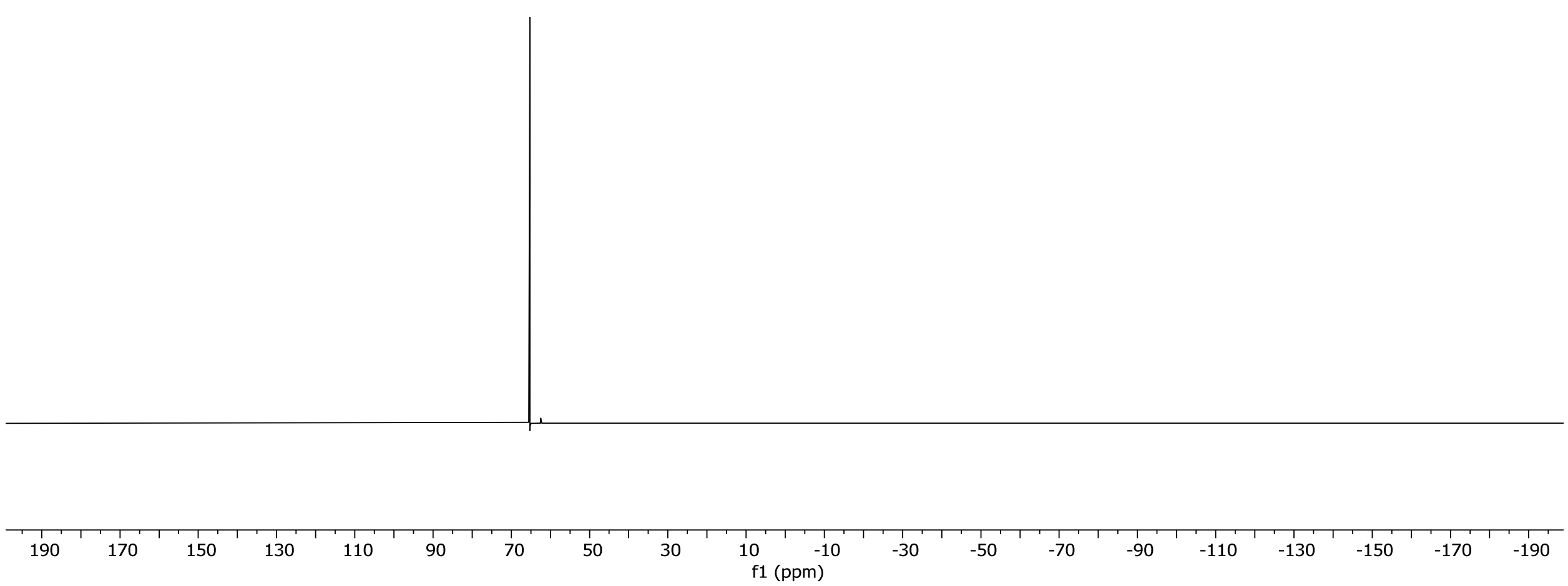

^{1}H ($CDCl_3$, 400 MHz) **15l**

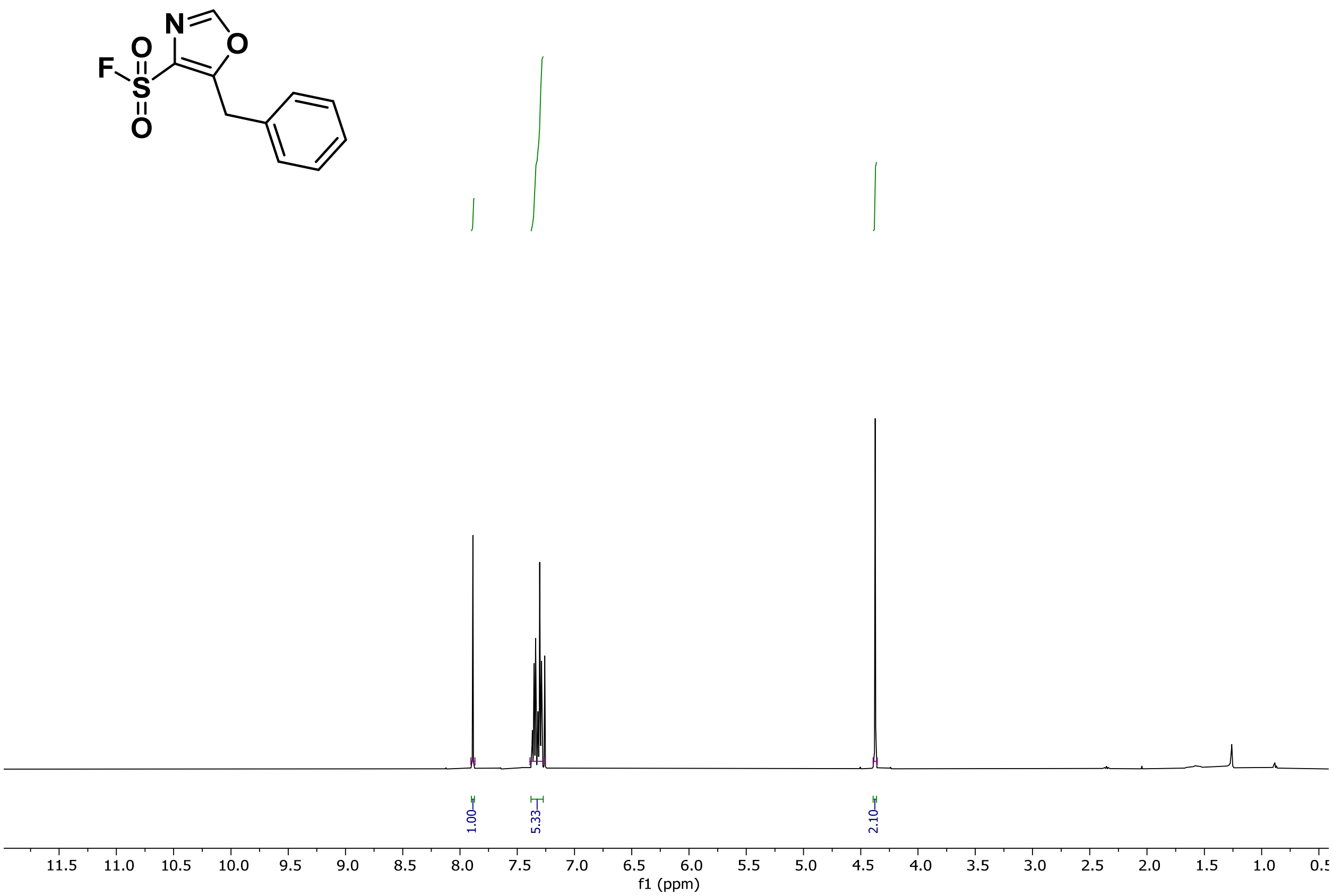

$^{13}C\{^{1}H\}$ ($CDCl_3$, 126 MHz) **15l**

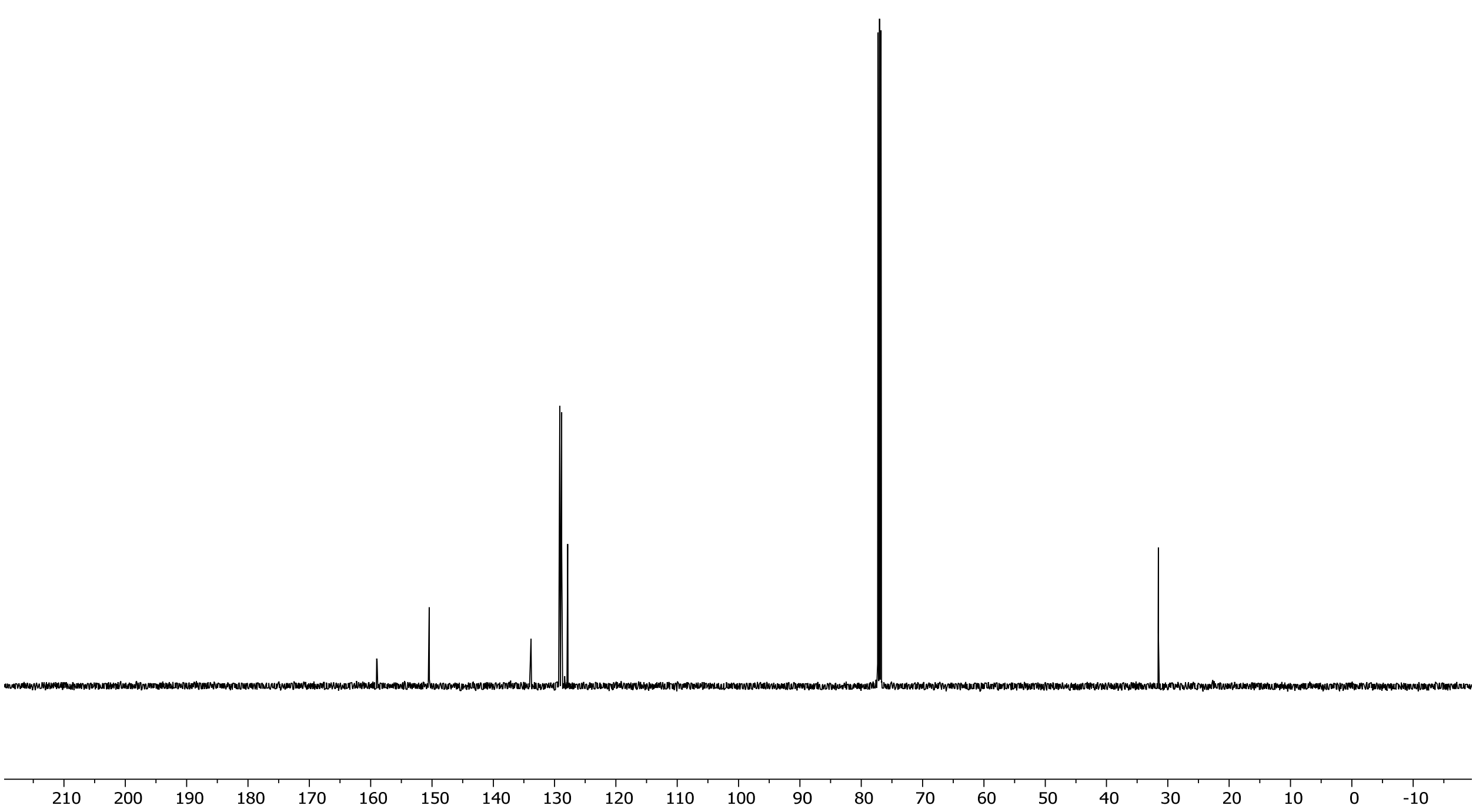

^{19}F {^{1}H, ^{13}C} NMR (376 MHz, $CDCl_3$) **15l**

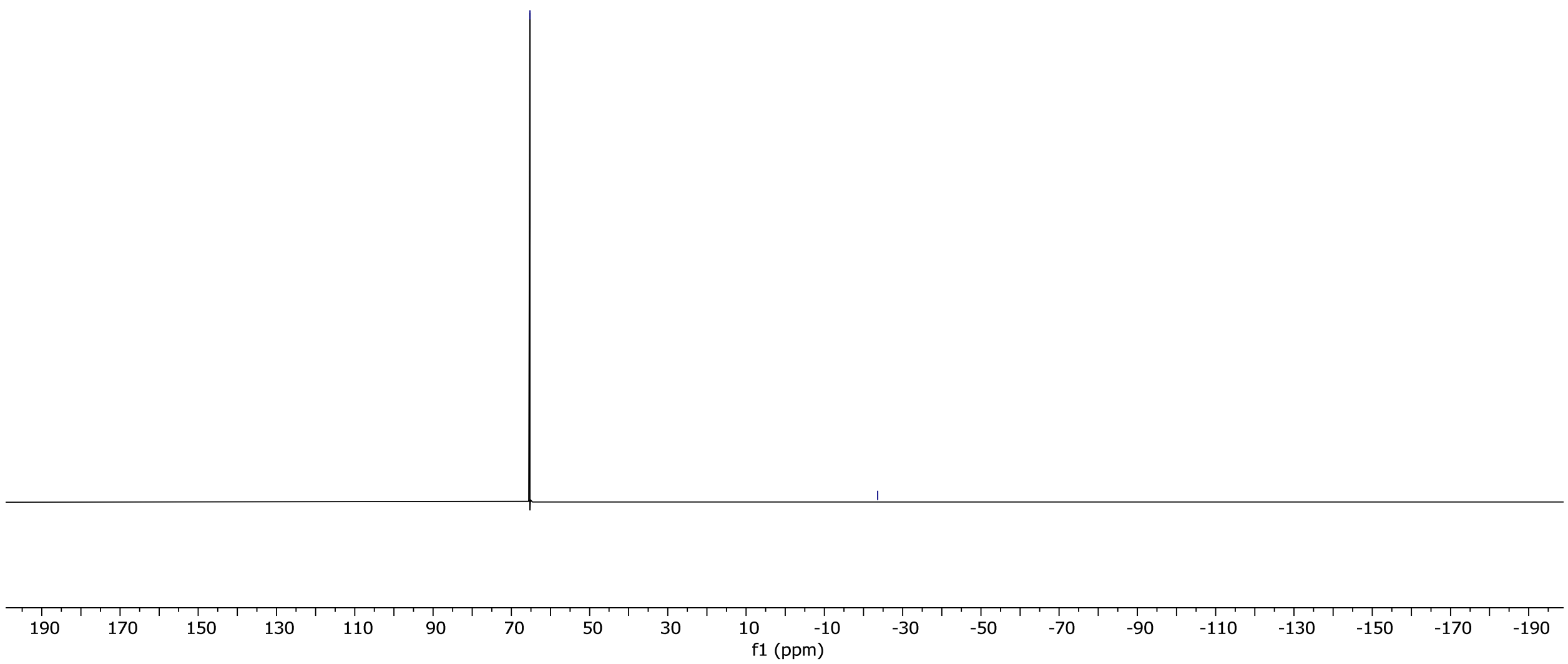

^{1}H ($CDCl_3$, 400 MHz) **16**

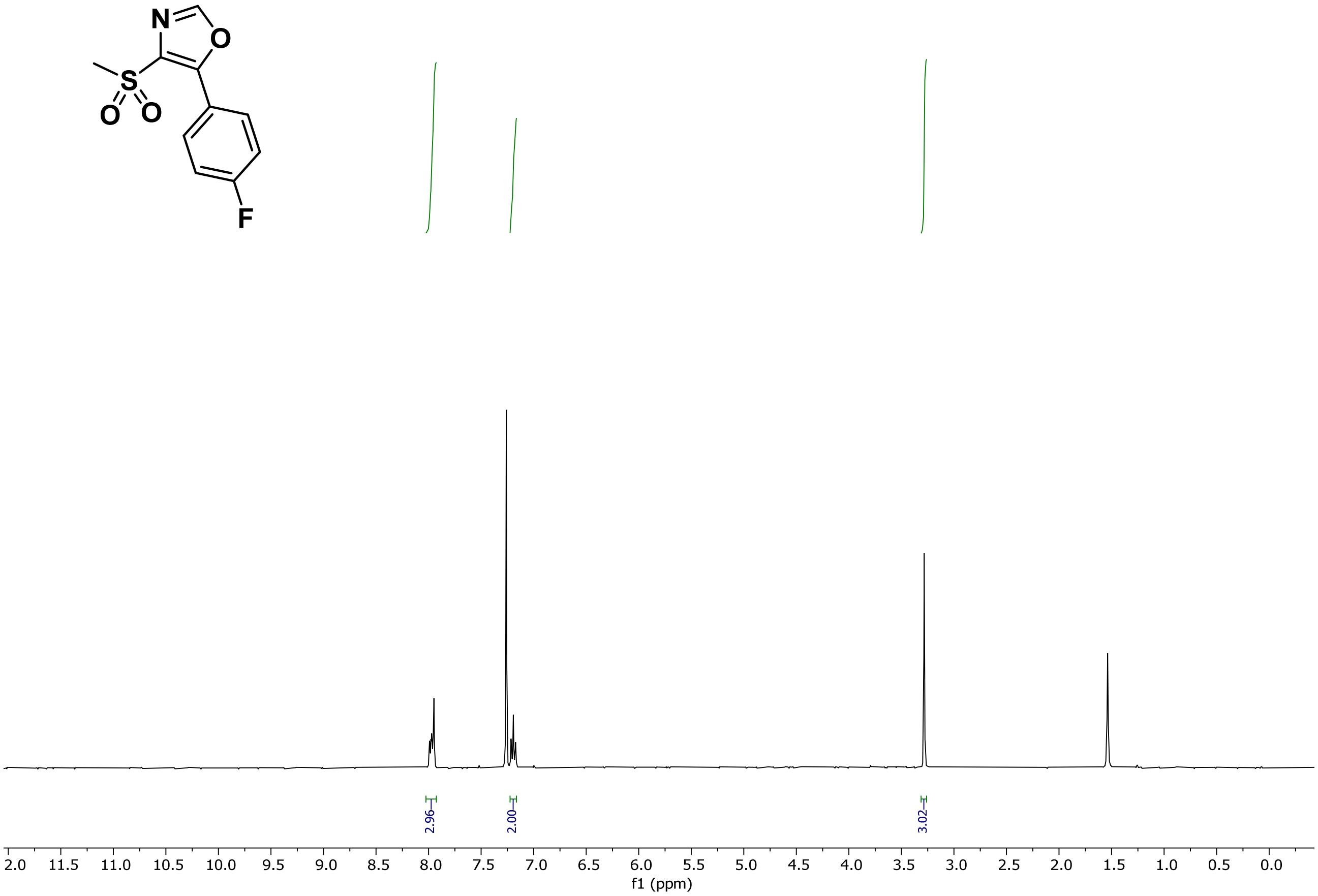

^{13}C{^{1}H} ($CDCl_3$; 101 MHz) **16**

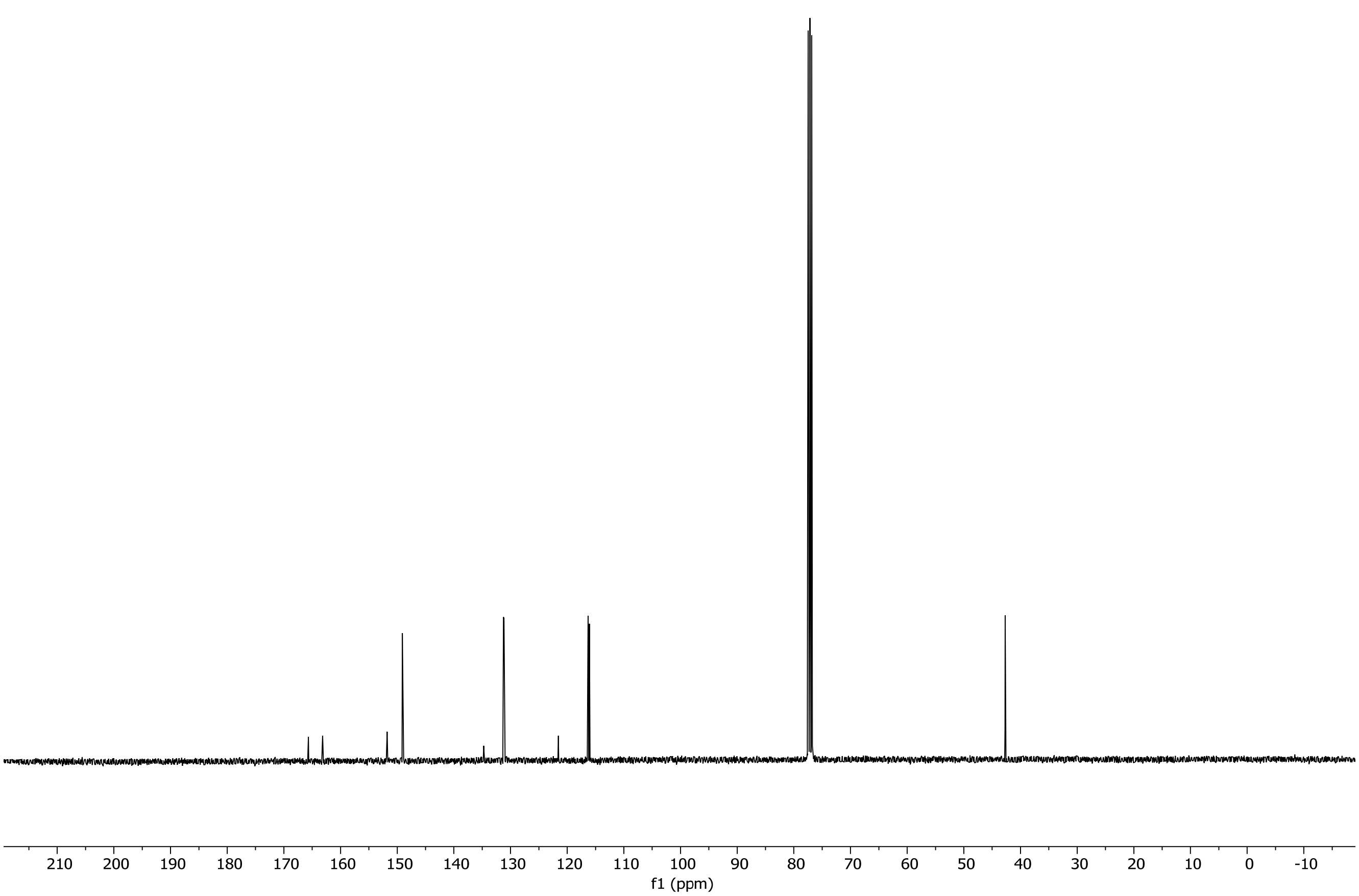

$^{19}F\{^{1}H, ^{13}C\}$ ($CDCl_3$, 376 MHz) **16**

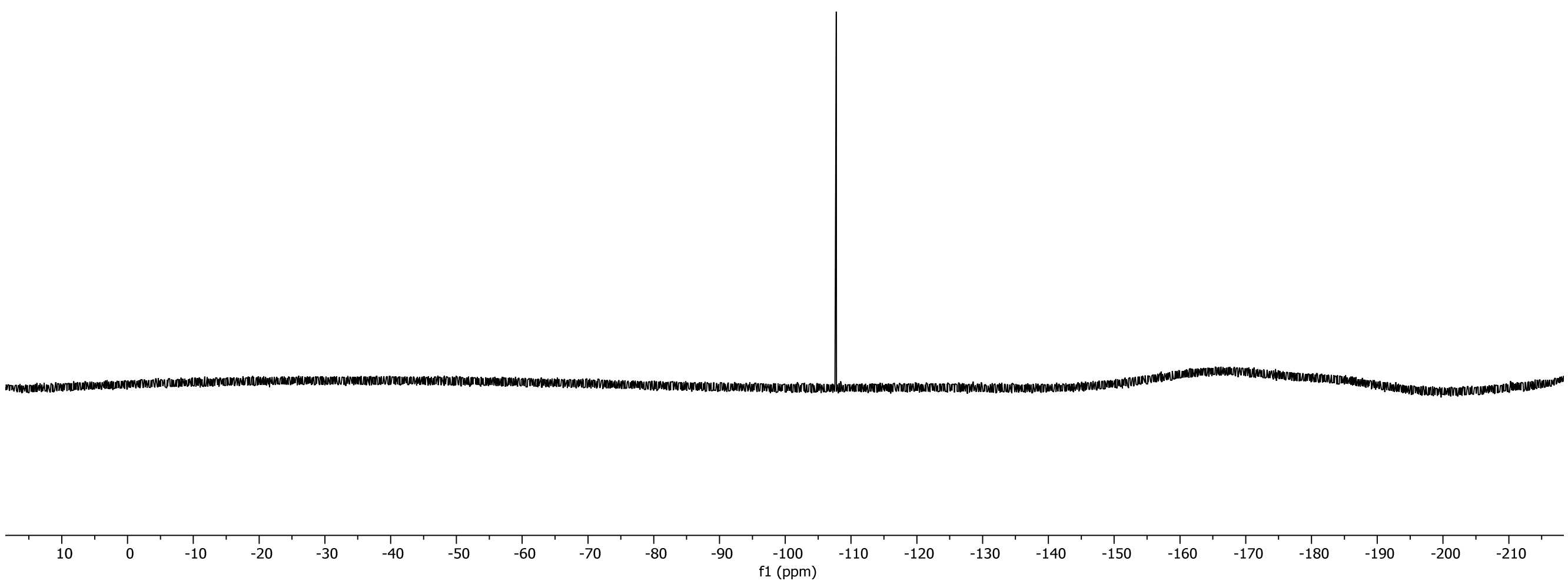

^{1}H ($CDCl_3$, 400 MHz) **17**

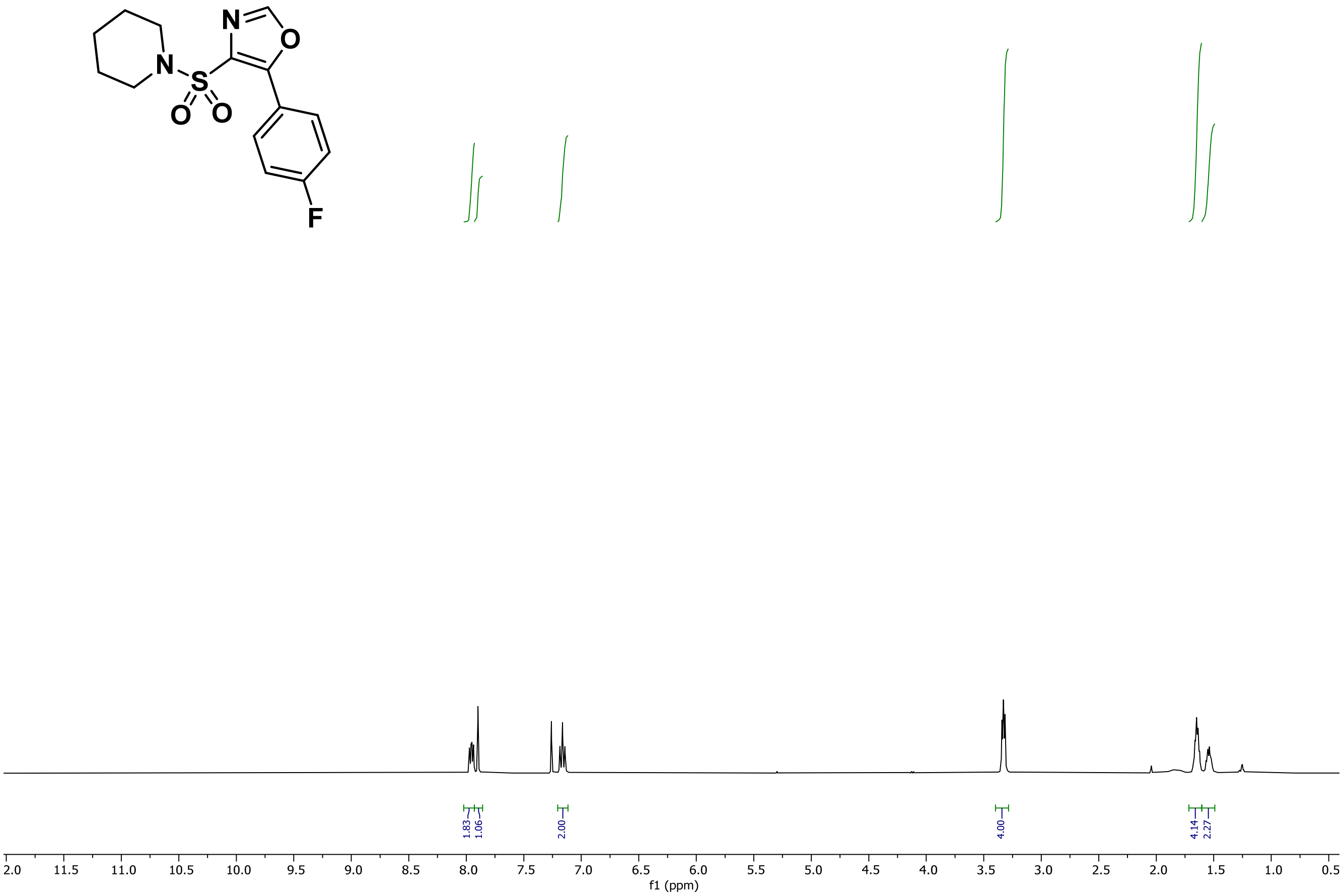

$^{13}C\{^{1}H\}$ ($CDCl_3$; 126 MHz) **17**

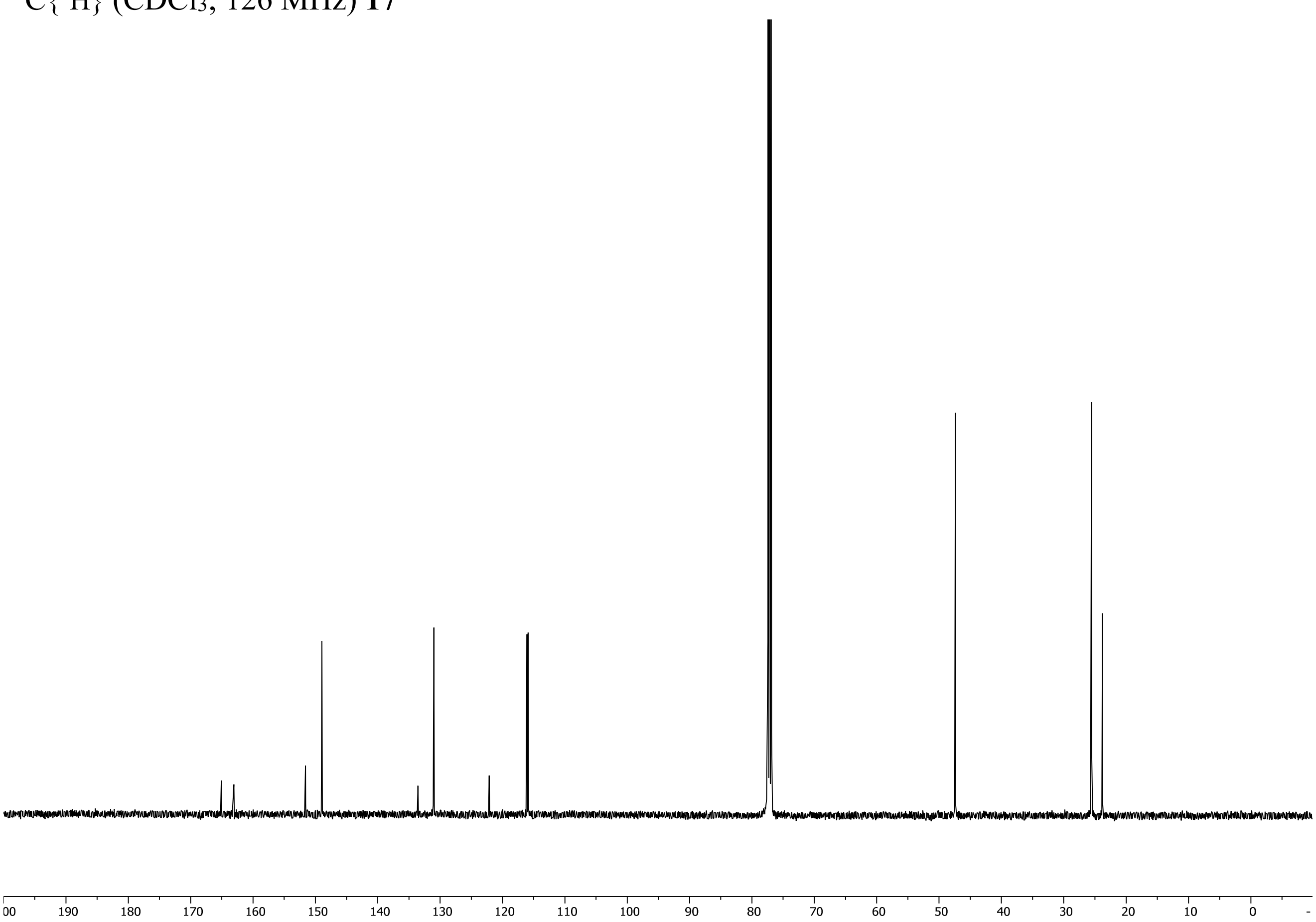

$^{19}F\{^{1}H, ^{13}C\}$ ($CDCl_3$, 376 MHz) **17**

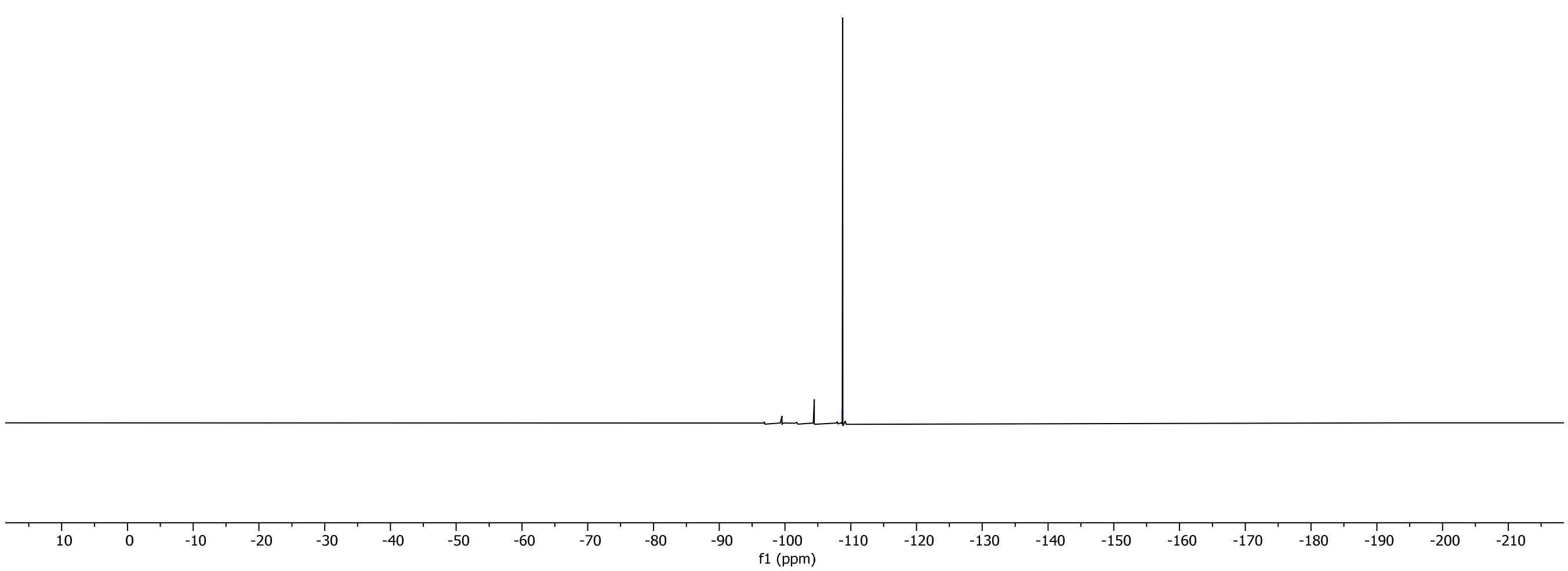

Supplement: Supplementary file 1 [file ol6c02962_si_001.pdf]
